# Supplementary material for: Effects of antihypertensives, lipid-modifying drugs, glycaemic control drugs and sodium bicarbonate on the progression of stages 3 and 4 chronic kidney disease in adults: a systematic review and meta-analysis
Source: BMJ Open. 2019 Sep 20;9(9):e030596. doi: 10.1136/bmjopen-2019-030596 (PMC6756484; doi:10.1136/bmjopen-2019-030596)
Supplement: Supplementary data [file bmjopen-2019-030596supp001.pdf]

SUPPLEMENTARY MATERIAL

Effects of antihypertensives, lipid-modifying drugs, glycaemic control drugs and sodium bicarbonate on the progression of stages 3 and 4 chronic kidney disease in adults: a systematic review and meta-analysis

Authors: Kathryn S Taylor, Julie McLellan, Jan Y Verbakel, Jeffrey Aronson, Daniel S Lasserson, Nicola Pidduck, Nia Roberts, Susannah Fleming, Christopher A O'Callaghan, Clare Bankhead, Amitava Banerjee, F.D. Richard Hobbs, Rafael Perera.

Corresponding author: Julie McLellan, Nuffield Department of Primary Care Health Sciences, University of Oxford, OX2 6GG, United Kingdom, julie.mclellan@phc.ox.ac.uk, 01865 289667

Contents

Search Strategy - MEDLINE Search Terms. 3

Supplementary Tables 7

Table 1. Kidney Disease Outcomes Quality Initiative classification (Levy 2003) 7

Table 2. Included drug list 8

Table 3. Characteristics of studies 12

Table 4. Interventions of studies 16

Table 5. Baseline characteristics 19

Table 6. Results of analyses 21

Table 7. GRADE assessments of primary outcome for each drug group 25

Supplementary Figures 27

Figure 1. Kidney Disease Improving Global outcomes (KDIGO) 2012 27

Figure 2. Cochrane Collaboration's Risk of bias 28

Figure 3. Funnel plots and contour enhanced funnel plots, for estimated glomerular filtration rate data, split by drug group 29

Figure 4. Ratio of means of estimated glomerular filtration rate at the end of the trials for anti-hypertensives vs comparator (boxes) and pooled estimates across studies (diamonds) calculated by the random effects DerSimonian and Laird method, for chronic kidney disease stages 3 and 4 and split by drug class 30

Figure 5. Ratio of means of proteinuria at the end of the trials for anti-hypertensives vs comparator (boxes) and pooled estimates across studies (diamonds) calculated by the random effects DerSimonian and Laird method, split by chronic kidney disease stage 31

Taylor KS, *et al. BMJ Open* 2019; 9:e030596. doi: 10.1136/bmjopen-2019-030596

Search Strategy - MEDLINE Search Terms.

# ▲	Searches
1	renal insufficiency/ or renal insufficiency, chronic/ or exp kidney failure, chronic/
2	(chronic adj (renal or kidney*) adj2 (fail* or insufficien* or disease? or disorder? or condition?)).ti,ab.
3	(late stage adj5 (kidney* or renal)).ti,ab.
4	((stage 3 or stage 3a or stage 3b or stage iii or stage iiia or stage iiib or stage 4 or stage iv) adj5 (kidney or renal)).ti,ab.
5	1 or 2 or 3 or 4
6	exp Angiotensin-Converting Enzyme Inhibitors/
7	((angiotensin adj2 converting enzyme inhibitor?) or ace inhibitor?).ti,ab.
8	(captopril or cilazapril or enalapril or fosinopril or imidapril or lisinopril or moexipril or perindopril or quinapril or ramipril or trandolapril).ti,ab.
9	exp Angiotensin Receptor Antagonists/
10	((angiotensin adj2 receptor antagonist?) or (angiotensin adj2 receptor blocker?) or sartans).ti,ab.
11	(ace or ace-i or ace-ii or acei or aceii or arb).ti,ab.
12	(azilsartan or candesartan or eprosartan or irbesartan or losartan or olmesartan or telmisartan or valsartan).ti,ab.
13	exp Thiazides/
14	thiazide*.ti,ab.
15	(Bendroflumethiazide or Chlortalidone or chlorthalidone or Cyclopenthiazide or Indapamide or Metolazone or Xipamide or Chlorothiazide or Hydrochlorothiazide).ti,ab.
16	exp Mineralocorticoid Receptor Antagonists/
17	aldosterone antagonist?.ti,ab.
18	(eplerenone or spironolactone).ti,ab.
19	exp Adrenergic beta-Antagonists/
20	(beta* blocker? or b-blocker? or beta* adrenoceptor antagonist? or b-adrenoceptor antagonist? or beta* adrenoceptor block* or b-adrenoceptor block* or beta* adrenergic block* or b adrenergic block*).ti,ab.
21	(propranolol or acebutolol or atenolol or bisoprolol or carvedilol or celiprolol or esmolol or labetalol or metropolol or nadolol or nebivolol or oxprenolol or pindolol or sotalol or timolol).ti,ab.
22	exp Calcium Channel Blockers/
23	(calcium channel block* or calcium antagonist?).ti,ab.
24	(amlodipine or diltiazem or felodipine or isradipine or lacidipine or lercanidipine or nicardipine or nifedipine or verapamil or nimodipine or nisoldipine).ti,ab.
25	exp Adrenergic alpha-Antagonists/
26	(alpha* blocker? or a-blocker? or alpha* adrenoceptor antagonist? or a-adrenoceptor antagonist? or alpha* adrenoceptor block* or a-adrenoceptor block* or postsynaptic alpha* block* or postsynaptic a-block*).ti,ab.
27	(doxazosin or indoramin or prazosin or terazosin).ti,ab.
28	Imidazoline Receptors/ag [Agonists]
29	(imidazoline receptor agonist? or centrally acting hypertensive*).ti,ab.

Taylor KS, *et al. BMJ Open* 2019; 9:e030596. doi: 10.1136/bmjopen-2019-030596

30	moxonidine.ti,ab.
31	(clonidine or methyldopa).ti,ab.
32	exp Vasodilator Agents/
33	vasodilator?.ti,ab.
34	(hydralazine or minoxidil).ti,ab.
35	Antihypertensive Agents/
36	(antihypertensive? or anti-hypertensive? or ((antihypertensive or anti-hypertensive) adj3 (drug? or agent? or therap* or treatment))).ti.
37	((blood pressure or bp) adj3 (drug? or agent? or therap* or treatment)).ti.
38	exp Insulins/
39	insulin?.ti,ab.
40	exp Sulfonylurea Compounds/
41	sulfonylurea?.ti,ab.
42	(Glibenclamide or gliclazide or glimepiride or glipizide or tolbutamide or chlorpropamide or glibornuride).ti,ab.
43	biguanides/ or metformin/
44	(biguanide? or metformin).ti,ab.
45	exp Glycoside Hydrolase Inhibitors/
46	(alpha glucosidase inhibitor? or acarbose).ti,ab.
47	Dipeptidyl-Peptidase IV Inhibitors/
48	(Dipeptidyl-Peptidase IV Inhibitor? or Dipeptidyl-Peptidase 4 Inhibitor? or DipeptidylPeptidase IV Inhibitor? or DipeptidylPeptidase 4 Inhibitor? or Dipeptidyl-Peptidase type IV Inhibitor? or Dipeptidyl-Peptidase type 4 Inhibitor? or DipeptidylPeptidase type IV Inhibitor? or DipeptidylPeptidase type 4 Inhibitor? or gliptin?).ti,ab.
49	(alogliptin or linagliptin or saxagliptin or sitagliptin or vildagliptin).ti,ab.
50	Thiazolidinediones/
51	(Thiazolidinedione? or glitazone?).ti,ab.
52	(pioglitazone or rosiglitazone).ti,ab.
53	(meglitinides? or nateglinide or repaglinide).ti,ab.
54	(Incretin mimetic? or glucagon-like peptide-1 activator? or GLP-1 activator?).ti,ab.
55	(exenatide or liraglutide or lixisenatide).ti,ab.
56	Sodium-Glucose Transporter 2/ai [Antagonists & Inhibitors]
57	(Sodium-glucose co-transporter 2 inhibitor? or SGLT2 inhibitor?).ti,ab.
58	(canagliflozin or dapagliflozin).ti,ab.
59	Hypoglycemic Agents/
60	(glyc?emic control adj3 (drug? or agent? or therap* or treatment)).ti.
61	exp Hydroxymethylglutaryl-CoA Reductase Inhibitors/
62	((hmg coa adj2 inhibitor?) or (Hydroxymethylglutaryl-CoA adj2 Inhibitors) or statin?).ti,ab.
63	(atorvastatin or fluvastatin or pravastatin or rosuvastatin or simvastatin or cerivastatin).ti,ab.
64	bile acid sequestran?.ti,ab.
65	(colesevelam or colestyramine or colestipol).ti,ab.
66	exp Fibric Acids/
67	(fibric acid? or fibrate?).ti,ab.

68	(bezafibrate or ciprofibrate or fenofibrate or gemfibrozil or clofibrate).ti,ab.
69	Nicotinic Acids/
70	(nicotinic acid? or acipimox).ti,ab.
71	exp *Fatty Acids, Omega-3/
72	(omega 3 fatty acid* or omega 3 marine triglyceride*).ti,ab.
73	(ezetimibe or lomitapide).ti,ab.
74	Hypolipidemic Agents/
75	((lipid lower* or hypolipidem* or cholesterol lower* or hypocholesterol*) adj2 (drug? or agent? or therap* or treatment)).ti,ab.
76	sodium bicarbonate/
77	sodium bicarbonate.ti,ab.
78	(Chortalidone or chorthalidone or chorpropamide or canaglifozin or dapaglifozin or canaglifloxin or dapaglifloxin or canaglifoxin or dapaglifoxin or giptin? or alogiptin or linagiptin or saxagiptin or sitagiptin or vildagiptin or Eposartan or Metopolol or popranolol or popranolole or propanalol or propanalole or propanolol or propranolole).ti,ab.
79	6 or 7 or 8 or 9 or 10 or 11 or 12 or 13 or 14 or 15 or 16 or 17 or 18 or 19 or 20 or 21 or 22 or 23 or 24 or 25 or 26 or 27 or 28 or 29 or 30 or 31 or 32 or 33 or 34 or 35 or 36 or 37 or 38 or 39 or 40 or 41 or 42 or 43 or 44 or 45 or 46 or 47 or 48 or 49 or 50 or 51 or 52 or 53 or 54 or 55 or 56 or 57 or 58 or 59 or 60 or 61 or 62 or 63 or 64 or 65 or 66 or 67 or 68 or 69 or 70 or 71 or 72 or 73 or 74 or 75 or 76 or 77 or 78
80	5 and 79
81	limit 80 to "reviews (maximizes specificity)"
82	randomized controlled trial.pt.
83	controlled clinical trial.pt.
84	randomized.ab.
85	placebo.ab.
86	drug therapy.fs.
87	randomly.ab.
88	trial.ab.
89	groups.ab.
90	82 or 83 or 84 or 85 or 86 or 87 or 88 or 89
91	exp animals/ not humans.sh.
92	90 not 91
93	80 and 92
94	limit 93 to yr="1999 -Current"
95	randomized controlled trial.pt.
96	controlled clinical trial.pt.
97	randomized.ab.
98	placebo.ab.
99	clinical trials as topic.sh.
100	randomly.ab.
101	trial.ti.
102	95 or 96 or 97 or 98 or 99 or 100 or 101
103	exp animals/ not humans.sh.
104	102 not 103

Taylor KS, *et al. BMJ Open* 2019; 9:e030596. doi: 10.1136/bmjopen-2019-030596

105	80 and 104
106	limit 105 to yr="1999 -Current"

Supplementary Tables

Table 1. Kidney Disease Outcomes Quality Initiative classification (Levy 2003)

Stages of CKD	eGFR ($ml/min/1.73m^2$)	Description
1	≥ 90	Normal or increased GFR, with other evidence of kidney damage
2	60–89	Slight decrease in GFR, with other evidence of kidney damage
3A	45–59	Moderate decrease in GFR, with or without other evidence of kidney damage
3B	30–44	
4	15–29	Severe decrease in GFR, with or without other evidence of kidney damage
5	< 15	Established renal failure

CKD – Chronic kidney disease; GFR – Glomerular filtration rate.

Taylor KS, *et al. BMJ Open* 2019; 9:e030596. doi: 10.1136/bmjopen-2019-030596

Table 2. Included drug list

Blood pressure lowering and/or Antihypertensive drugs	Angiotension-converting enzyme inhibitors and/or ACE inhibitors and/or ACEi	Benazepril
		Captopril
		Cilazapril
		Enalapril maleate
		Fosinopril sodium
		Imidapril hydrochlorine
		Lisinopril
		Moexipril hydrochoride
		Perindopril erbumine
		Perindopril arginine
		Quinapril
		Ramipril
		Trandolapril
	Angiotension-II receptor antagonists and/or ARBs and/or angiotension receptor blockers and/or sartans	Azilsartan medoxomil
		Candesartan
		Eprosartan
		Irbesartan
		Losartan potassium
		Olmesartan medoxomil
		Telmisartan
		Valsartan
	Thiazides and/or thiazide diuretics and/or thiazide-like diuretics	Bendroflumethiazide
		Chlortalidone or chlorthalidone
		Cyclopenthiazide
		Indapamide
		Metolazone
		Xipamide
		Chlorothiazide
		Hydrochlorothiazide
	Aldosterone antagonists	Eplerenone
		Spironolactone
	Beta-adrenoceptor antagonists and /or beta-blockers and/or beta-adrenoceptor blocking drugs and/or beta-adrenergic blockers	Propranolol hydrochloride
		Acebutolol
		Atenolol
		Bisoprolol flumarate
		Carvedilol
		Celiprolol hydrochloride
		Esmolol hydrochloride
		Labetalol hydrochloride
		Metoprolol tartrate
		Nadolol

Taylor KS, *et al. BMJ Open* 2019; 9:e030596. doi: 10.1136/bmjopen-2019-030596

		Nebivolol
		Oxprenolol hydrochloride
		Pindolol
		Sotalol hydrochloride
		Timolol Maleate
	Calcium channel blockers and/or calcium channel blocking drugs and/or calcium antagonists	Amlodipine
		Diltiazem hydrochloride
		Felodipine
		Isradipine
		Lacidipine
		Lercanidipine hydrochloride
		Nicardipine
		Nifedipine
		Verapamil hydrochloride
		Nimodipine
		Nisoldipine
	Alpha adrenoceptor antagonists and/or alpha-adrenoceptor blocking drugs and/or alpha blockers and/or alpha1 blockers and/or postsynaptic alpha blockers	Doxazosin
		Indoramin
		Prazosin
		Terazosin
	Alpha-2-adrenoceptor agonists and/or centrally acting antihypertensive drugs	Clonidine
		Methyldopa
	Imidazoline receptor antagonists and/or centrally acting antihypertensive drugs	Moxonidine
	Vasodilators	Hydralazine hydrochloride
		Minoxidil
Glycaemic control	Insulin	Insulin
		Insulin aspart
		Insulin lispro
		Insulin degludec
		Insulin detemir
		Insulin glargine
		Insulin zinc suspension
		Isophane insulin
		Protamine zinc insulin
		Biphasic insulin aspart
		Biphasic insulin lispro
		Biphasic isophane insulin
	Sulfonylureas	Glibenclamide
		Gliclazide
		Glimepiride

Taylor KS, *et al. BMJ Open* 2019; 9:e030596. doi: 10.1136/bmjopen-2019-030596

		Glipizide
		Tolbutamide
		Chlorpropamide
		Glibornuride
	Biguanides	Metformin hydrochloride
	Alpha glucosidase inhitors	Acarbose
	Dipeptidylpeptidase type 4 inhibitors and/or gliptins	Alogliptin
		Linagliptin
		Saxagliptin
		Sitagliptin
		Vildagliptin
	Thiazolidinediones and/or glitazones	Pioglitazone
		Rosiglitazone
	Meglitinides	Nateglinide
		Repaglinide
	Incretin mimetics and/or glucagon-like peptide-1 activators and/or GLP-1 activators	Exenatide
		Liraglutide
		Lixisenatide
	Sodium-glucose co-transporter 2 inhibitors and/or SGLT2 inhibitors	Canagliflozin
		Dapagliflozin
Lipid regulating drugs	Statins	Atorvastatin
		Fluvastatin
		Pravastatin
		Rosuvastatin
		Simvastatin
		Cerivastatin
	Bile acid sequestrants	Colesevelam hydrochloride
		Colestyramine
		Colestipol hydrochloride
	Fibrates	Bezafibrate
		Ciprofibrate
		Fenofibrate
		Gemfibrozil
		Clofibrate
	Nicotinic acid derivatives	Acipimox
		Nicotinic acid
	Omega 3 fatty acid compounds	Omega-3-acid ethyl esters
		Omega-3-marine triglycerides
	Miscellanous	Ezetimibe
		Lomitapide
Bicarbonate(s)	Sodium bicarbonate	

Reduction/Lowering of proteinuria	No specific drugs - usually additional response to blood pressure and glycaemic control drugs	

Table 3. Characteristics of studies

Study (trial name/author)	Year	Drug class	Trial N*	Analysis N*	Follow up (mths)**	CKD stages†	Population that we studied [method of obtaining data on subgroup for analysis]	Country
ADVANCE[79 80] §	2010 2012	A	10640	2184	60	3 and 4	Type II diabetes and, most likely, CKD stages 1 to 5. [Authors gave data for stages 3 and 4]	Multinational
Bianchi 2010[66]	2010	A	128	128	36	1 to 3	Idiopathic chronic glomerulonephritis and CKD stages 1 to 3	Italy
CHARM[38-40] (3 trials) §	2003 2006 2016	A	7599	463/845	26	3 and 4	Symptomatic chronic heart failure and, most likely, CKD stages 1 to 5. [Authors gave data for stages 3 and 4]	Multinational
EMPHASIS-HF[91 92]	2001 2013	A	2737	2737	21	1 to 3	Systolic heart failure and CKD stages 1, 2 and 3a.	Multinational
ESBARI[44] §	2010	A	224	116	41	4	Advanced chronic renal insufficiency without diabetes and CKD stages 4 and 5. [Authors gave data for stage 4]	China
HALT-PKD[52] §	2001	A	486	468	24	3 and 4	Moderately advanced autosomal dominant polycystic kidney disease and CKD stages 3 and 4. [Authors gave further data]	USA
HYVET[81-83] §	2001 2008 2012	A	3845	686	25	3	Very elderly hypertensive population and, most likely, CKD stages 1 to 5. [Authors gave data for stage 3]	Multinational
IDNT[53-56]	1992 2003 2011 2014	A	1715	995	36	3	Hypertensive and with diabetic nephropathy due to type II diabetes and CKD stages 1 to 5 [Stage 3 data was published]	USA
PREVEND IT[67]	2009	A/L‡	864	864	46	1 to 3	Microalbuminuria and CKD stages 1 to 3.	Netherlands
RENAAL[93-97]	2002 2004 2005 2006	A	1513	1513	41	2 to 4	Type II diabetic nephropathy and CKD stages 2 to 4.	Multinational

Taylor KS, *et al. BMJ Open* 2019; 9:e030596. doi: 10.1136/bmjopen-2019-030596

	2014							
SAVE[57] §	1998	A	2231	521	36	3a	Left ventricular dysfunction after M. CKD stages 1 to 5. [Authors gave data for stage 3a]	USA/Canada
SUPPORT[46 47]	2013 2016	A	1147	1147	53	1 to 4	Hypertensive with heart failure. CKD stages 1 to 4.	Japan
TOPCAT[98]	2007	A	3445	3445	40	1 to 3	Symptomatic heart failure and preserved ejection fraction.	Multinational
4S[68-70]	1993 2000 2003	L	4444	409	66	3	Coronary heart disease and elevated LDL and, most likely, CKD stages 1 to 5. [Stage 3 data was published]	Europe
AFCAPS/ TEXCAPS[58-60]	1997 2001 2010	L	6605	304	61	2 to 4	With CKD without significant diabetes or cardiovascular disease [Stage 3 and 4 data was published]	USA
ASCOT-LLA[71 72] §	2011 2007	L‡	10305	10305	40	1 to 3	Hypertension and, most likely, CKD stages 1 to 5. [Stage 1 to 3 data was published and authors gave further data]	Europe
ASUCA[48] §	2004	L	349	279/334	24	3 and 4	Dyslipidemia. [Stage 3 and 4 data was published and authors gave further data]	Japan
ATIC[73]	2008	L	93	93	24	2 to 4	Mild/moderate CKD.	Netherlands
FIELD[84-86]	2004 2005 2015	L	9795	519	60	3	Type II diabetes and renal impairment. CKD stages 1 to 3 [Stage 3 data was published]	Multinational
GISSI-HF[74 75] §	2004 2007	L	4631	1043	36	3 and 4	Chronic heart failure. [Obtained data on stages 3 and 4 from the company that owns the data]	Italy
GREACE[76 77]	2002 2009	L	1600	1600	36	1 to 3	Coronary heart disease, and CKD stages 1 to 3. [Authors reported a post-hoc analysis on those with and without metabolic syndrome]	Greece
LORD[45]	2015	L	123	123	29	2 to 4	CKD stages 2 to 4.	Tasmania

MEGA[49-51]	2006<br>2009<br>2014	L	7832	2978	64	3	Hypercholesterolemia and normal kidney function. CKD stages 1 to 3. [Stage 3 data was published]	Japan
METEOR[99]	1999	L	984	984	24	1 to 3	Asymptomatic individuals with moderately elevated cholesterol and low risk of cardiovascular disease.	Multinational
PROSPER[78 100 101]	2002<br>2007<br>2011	L	5796	3094	24	3 and 4	With pre-existing risk of vascular disease or risk of developing this condition. Most likely with CKD stages 1 to 5. [Stage 3 and 4 data was published]	Europe
SHARP[102-104]	2010<br>2014	L	9270	4808/4720	58	1 to 4	CKD stages 1 to 5. [Stage 1 to 4 data was published with graphical data separating Stages 1 to 3 with Stage 4 data]	Multinational
VA-HIT[61-63]	1993<br>2004<br>2017	L	399	399	24	3	Coronary disease and low HDL. Most likely CKD stages 1 to 5.<br>[Stage 3 data was published]	USA
Bode 2015[105 106]	2013	G	714	503	24	1 to 3	Type II diabetes and CKD.	Multinational
CANVAS Program[19 20] (2 trials) §	2017<br>2018	G	10142	496¶	29	3	Two trials of populations with type II diabetes and high cardiovascular risk with CKD stages 1 to 4. [Stage 3a and 3b data was published]	Multinational
EMPA-REG OUTCOME[87 88]	2016	G	7020††	1819/7020	36	3 §§	Type II diabetes and CKD stages 1 to 3.<br>[Stage 3 data was published]	Multinational
Kohan 2014[107]	2009	G	252	252	24	2 to 4	Type II diabetes and moderate renal impairment with CKD stages 2 to 4.	Multinational
Leiter 2016[41-43] (2 trials)	2006<br>2014<br>2015	G	1887	474	24	1 to 3	Two trials of populations with type II diabetes and cardiovascular disease with CKD stages 1 to 3. One of the trials also included participants with hypertension.	Multinational
TECOS[89 90]	2010<br>2015	G	14671	567/3321	36	3	Type II diabetes and cardiovascular disease and CKD stages 1 to 3.<br>[Stage 3 data was published]	Multinational

Brito-Ashurst 2009[13]	2009	B	134	134	24	4	Stage 4 chronic CKD (creatinine clearance 15 to 30 ml/min per 1.73 m^2 and metabolic acidosis (serum bicarbonate 16 to 20 mmol/l)	UK
Goraya 2017[64 65]	2010 2014	B	108	108	60	3	Stage 3 CKD and metabolic acidosis (plasma TCO_2 22 to 24 mmol/l)	USA

* Trial population size (single or combined trials) and analysis population (if two numbers are given, the first refers to the primary outcome); ** mean or median; † CKD stages are given for the analysis population; †† 7028 patients were randomised; ‡ 2X2 factorial trial; §Unpublished data was provided by the authors; §§ - Used data for CKD stage 3 in the main analysis and data for CKD stages 1 to 3 as a sensitivity analysis; ¶- Included eGFR data from time before longest follow-up. CKD – chronic kidney disease. Drug class: A - antihypertensive; L – lipid-modifying; G – glycaemic control; B- sodium bicarbonate.

Taylor KS, *et al. BMJ Open* 2019; 9:e030596. doi: 10.1136/bmjopen-2019-030596

Table 4. Interventions of studies

Study	Year	Drug class	Drug type(s)	Interventions (dose and frequency, if stated)
ADVANCE[79 80]	2010 2012	A	ACE + thiazide	Perindopril (2 to 4 mg daily) + indapamide (0.625 to 1.25 mg daily) vs placebo
Bianchi 2010[66]	2010	A	ACE+ ARB+ aldost. antag. vs Thiazide+BB+ARB+CCA	Ramipril (initially 10 mg daily) + irbesartan (300 mg daily) + atorvastatin (20 mg daily) + spironolactone (25 mg daily) vs ramipril (10 mg daily) + atorvastatin (10 mg daily), possibly + hydrochlorothiazide or furosemide, calcium antagonists, beta-blockers or alpha-receptor antagonists.
CHARM[38-40]	2003 2006 2016	A	ARB	Candesartan (4 or 8 mg to 32 mg daily) vs placebo
EMPHASIS-HF[91 92]	2001 2013	A	Aldost. antag.	Eplerenone (25 to 50 mg daily) vs placebo
ESBARI[44]	2010	A	ACE	Benazepril (20 mg twice a day) vs placebo
HALT-PKD[52]	2001	A	ARB	Telmisartan (40 to 80 mg daily) vs placebo
HYVET[81-83]	2001 2008 2012	A	ACE + thiazide	Indapamide initially (1.5 to 2 or 4 mg daily) and possibly + perindopril later to achieve target blood pressure vs placebo
IDNT[53-56]	1992 2003 2011 2014	A	ARB CCA	Irbesartan (75 to 100 mg daily) vs amlodipine (2.5 to 10 mg daily) vs placebo
PREVEND IT[67]	2009	A/L	Anti-hypertensive: ACE Lipid modifying: Statin	2x2 factorial design: fosinopril (20 mg daily) or matching placebo and pravastatin (40 mg daily) or matching placebo Data for antihypertensive analysis: (fosinopril+pravastatin or fosinopril+placebo) vs (placebo only or placebo + pravastatin) Data for lipid-modifying analysis: (fosinopril+pravastatin or pravastatin+placebo) vs (placebo only or placebo + fosinopril)
RENAAL[93-97]	2002 2004 2005	A	ARB	Losartan (5 to 100 mg daily) vs placebo

Taylor KS, *et al. BMJ Open* 2019; 9:e030596. doi: 10.1136/bmjopen-2019-030596

	2006<br>2014			
SAVE[57]	1998	A	ACE	Captopril (6.5 to 50 mg 3 times a day) vs placebo
SUPPORT[46 47]	2013<br>2016	A	ARB	Olmesartan (5-10mg and increased to 40mg daily) vs placebo
TOPCAT[98]	2007	A	Aldost. antag.	Spironolactone (15-45 mg daily) vs placebo
4S[68-70]	1993<br>2000<br>2003	L	Statin	Simvastatin (20 to 40 mg daily) vs placebo
AFCAPS/TEXCAPS[58-60]	1997<br>2001<br>2010	L	Statin	Lovastatin (20 mg daily) vs placebo
ASCOT-LLA[71 72]	2011<br>2007	L	Statin	2x2 factorial design: atorvastatin (10 mg daily) + amlodipine (5 to 10 mg daily) vs atorvastatin (10 mg daily) + atenolol (50 to 100mg daily) vs placebo + amlodipine (5 to 10 mg daily) vs placebo + atenolol (50 to 100 mg daily)<br>Data for atenolol analysis: (atorvastatin + atenolol) vs (placebo + atenolol)<br>Data for amlodipine analysis: (atorvastatin + amlodipine) vs (placebo + amlodipine)
ASUCA[48]	2004	L	Statin	Atorvastatin (10 to 5-20 mg daily) vs diet
ATIC[73]	2008	L	Statin+ other	Pravastatin (40 mg daily) + vitamin E (300 mg daily) + folic acid (15 mg daily) + pyridoxine hydrochloride (100 mg daily) + cyanacobalamin (1 mg daily) vs placebo
FIELD[84-86]	2004<br>2005<br>2015	L	Fibrate	Fenofibrate (200 mg daily) vs placebo
GISSI-HF[74 75]	2004<br>2007	L	Statin	Rosuvastatin (10mg) daily vs placebo
GREACE[76 77]	2002<br>2009	L	Statin	Atorvastatin (10 to 80 mg daily) vs usual care
LORD[45]	2015	L	Statin	Atorvastatin (10 mg daily) vs placebo
MEGA[49-51]	2006<br>2009	L	Statin	Pravastatin (10 mg initially) + diet vs diet

	2014			
METEOR[99]	1999	L	Statin	Rosuvastatin (40 mg daily) vs placebo
PROSPER[78 100 101]	2002<br>2007<br>2011	L	Statin	Pravastatin (40 mg daily) vs placebo
SHARP[102-104]	2010<br>2014	L	Statin	Simvastatin (20 mg daily) + ezetimibe (10 mg daily) vs placebo
VA-HIT[61-63]	1993<br>2004<br>2017	L	Fibrate	Gemfibrozil (1200 mg daily) vs placebo
Bode 2015[105 106]	2013	G	Flozin	Canagliflozin (100 mg daily) vs canagliflozin (300 mg daily) vs placebo
CANVAS Program[19 20]	2017<br>2018	G	Flozin	Canagliflozin (100mg-300mg) vs placebo
EMPA-REG OUTCOME[87 88]	2016	G	Flozin	Empagliflozin (25mg daily) vs empagliflozin (10mg daily) vs placebo
Kohan 2014[107]	2009	G	Flozin	Dapagliflozin (10 mg daily) vs dapagliflozin (50 mg daily) vs placebo
Leiter 2016[41-43]*	2006<br>2014<br>2015	G	Flozin	Dapagliflozin (10 mg daily) vs placebo
TECOS[89 90]	2010<br>2015	G	Gliptin	Sitagliptin (100 mg daily) vs placebo
Brito-Ashurst 2009[13]	2009	B	Sodium bicarbonate	Oral sodium bicarbonate (600 mg 3 times a day) vs routine standard care
Goraya 2017[64 65]	2010<br>2014	B	Sodium bicarbonate	Oral sodium bicarbonate (> 22 mq/L) vs routine care

* Reported in Leiter 2014 and Cefalu 2015. Drug class: A – antihypertensive; L – lipid-modifying; G – glycaemic control; B – sodium bicarbonate. Drug subclass: ACE – angiotension converting enzyme inhibitor; ARB - angiotensin II receptor blockers; Aldost. antag. - aldosterone antagonists; BB – beta-blocker; CCA – calcium channel antagonist.

Taylor KS, *et al. BMJ Open* 2019; 9:e030596. doi: 10.1136/bmjopen-2019-030596

Table 5. Baseline characteristics

Trial	Trial N	Baseline N	Analysis N	CKD stages*	Drug class	Age Mean (SD)	Male (%)	Non-white ethnicity (%)	Current Smoker (%)	Body mass index Mean (SD)	Systolic blood pressure Mean (SD)	Diastolic blood pressure Mean (SD)
Trials for which baseline data was provided for all patients included in the analysis												
ADVANCE[79 80]	10640	2184	2184/ 2033	3 to 4	A	68.2 (6.4)	43.1	NS	10.4	28.7 (5.2)	147.0 (23.3)	79.8 (11.3)
Bianchi 2010[66]	128	128	128	1 to 3	A	53.2 (0.9)	64.1	NS	28.9	25.1 (0.3)	156.1 (1.3)	93.7 (0.7)
EMPHASIS-HF[91 92]	2737	2737	2737	1 to 3	A	68.6 (7.6)	77.5	17.1	NS	27.5 (4.9)	124.0 (17.0)	75.0 (10.0)
PREVEND IT[67] (A)	864	864	864	1 to 3	A	51.3 (11.8)	64.9	3.9	39.9	26.0 (4.5)	130.0 (17.5)	76.0 (10.0)
PREVEND IT[67] (L)	864	864	864	1 to 3	L	51.3 (11.8)	65.0	3.9	38.2	26.0 (4.0)	130.5 (17.5)	76.5 (10.0)
RENAAL[93-97]	1513	1513	1513	2 to 4	A	60.0 (7.0)	63.2	51.3	18.3	29.5 (6.0)	152.5 (19.5)	82.0 (10.5)
TOPCAT[98]	3445	3445	3445	1 to 3	A	68.7 (11.2)	48.5	11.1	10.5	31 (6.7)	130 (14.5)	80.0 (7.4)
4S[68-70]	4444	409	409	3	L	62.2 (5.0)	54.5	NS	16.1	25.9 (3.4)	143.1 (21.4)	83.7 (9.6)
AFCAPS/ TEXCAPS[58-60]	6605	304	304	2 to 4	L	62.0 (7.5)	78.3	1.0	7.9	26.5 (3.0)	141.6 (18.0)	79.0 (10.0)
ATIC[73]	93	93	93	2 to 4	L	53.0 (12.0)	56.9	NS	35.5	26.5 (4.5)	135.0 (20.9)	78.5 (12.0)
FIELD[84-86]	9795	519	519	3	L	66.5 (5.9)	41.2	NS	6.9	NS	NS	NS
GREACE[76 77]	1600	1600	1600	1 to 3	L	58.5 (7.5)	79.4	NS	NS	25.8 (4.4)	129.0 (16.9)	77.5 (7.9)
LORD[45]	123	123	123	2 to 4	L	60.2 (15.1)	65.0	0	NS	28.5 (6.0)	143.4 (19.8)	80.7 (12.6)
MEGA[49-51]	7832	2978	2978	3	L	60.0	24.3	NS	12.5	NS	132.9	NS
PROSPER[78 100 101]	5796	3094	3094	3	L	75.5 (3.4)	42.0	NS	22.9	27.0 (4.3)	154.7 (21.6)	83.7 (11.3)
VA-HIT[61-63]	2531	399	399	3	L	67.3 (5.2)	100	9.5	14.0	NS	134.0 (18.3)	77.2 (9.8)
Kohan 2014[107]	252	252	252	2 to 4	G	67.0 (8.1)	65.1	16.3	NS	NS	132.1 (16.4)	73.3 (9.3)
EMPA-REG OUTCOME[87 88]	7020†	1819	1819	3	G	67.1 (7.8)	67.8	NS	NS	31.0 (5.5)	136.2 (18.2)	74.5 (10.0)
Brito-Ashurst 2009[13]	134	134	134	4	B	54.8 (2.4)	51.5	48.0	NS	NS	123.9 (1.3)	75.8 (1.7)
Goraya 2017[64 65]	108	108	108	3	B	53.7 (5.1)	44.0	NS	NS	NS	162.3 (11.1)	NS

Taylor KS, *et al. BMJ Open* 2019; 9:e030596. doi: 10.1136/bmjopen-2019-030596

Trials for which baseline data was not provided for all patients included in the analysis but for a different subgroup of the trial population or the whole trial population(s)												
CHARM[38-40] (3 trials)	7599	2680	463/845	3 to 4	A	65.3 (11.6)	66.6	NS	14.2	NS	128.2 (18.7)	73.6 (10.7)
ESBARI[44]	224	224	116/224	4	A	44.7 (15.5)	49.5	NS	NS	22.8 (5.0)	152.4 (23.1)	85.9 (10.4)
HYVET[81-83]	3845	1194	686	3	A	83.8 (3.3)	31.0	NS	NS	25.0 (3.8)	173.7 (8.9)	NS
HALT-PKD[52]	486	486	468	3 to 4	A	48.7 (8.3)	48.3	6.5	NS	28.0 (5.2)	121.5 (15.6)	75.9 (10.9)
IDNT[53-56]	1715	1715	995	3	A	58.9 (7.8)	66.3	27.5	NS	30.8 (5.8)	159.0 (19.7)	87.0 (11.0)
SAVE[57]	2231	2231	521	3a	A	59.4	82.5	NS	NS	NS	112.5	70.0
SUPPORT[46 47]	1147	1147	1139	1 to 4	A	65.7 (10.2)	74.7	NS	NS	24.4 (4.1)	127.9 (18.1)	74.4 (12.0)
ASCOT-LLA[71 72]	10305	10305	10305	1 to 3	L	63.1 (8.5)	81.2	NS	32.7	23.6 (6.9)	164.2 (17.8)	95.0 (10.3)
ASUCA[48]	349	334	279/334	3 to 4	L	63.2 (8.1)	63.8	NS	14.1	25.6 (3.6)	133.2 (16.3)	76.8 (10.6)
GISSI-HF[74 75]	4631	4574	1043	3 to 4	L	68.0 (11.0)	77.4	NS	14.0	27.1 (4.5)	127.0 (18.0)	77.0 (10.0)
METEOR[99]	984	984	900	1 to 3	L	57.0 (6.0)	59.7	15.7	4.0	27.2 (4.0)	139.0 (21.0)	80.0 (12.0)
SHARP[102-104]	9270	6245	4808/ 4720	1 to 4	L	63.0 (12.0)	62.5	28.5	12.0	27.4 (5.5)	139.0 (21.0)	80.0 (12.0)
Bode 2015[105 106]	714	714	503	1 to 3	G	63.6 (6.2)	55.5	23.1	NS	31.6 (4.6)	NS	NS
CANVAS Program[19 20] (2 trials)	10142	10142	496	3	G	63.3 (8.3)	64.2	21.7	17.8	32.0 (5.9)	136.6 (15.8)	77.7 (9.7)
Leiter 2016[41-43] (2 trials)	1887	568	474/568	1 to 3	G	62.9 (7.3)	93.9	NS	NS	32.7 (5.5)	133.8 (13.5)	77.6 (8.5)
TECOS[89 90]	14671	3321	567/332 1	3	G	68.3 (7.8)	63.6	68.7	7.4	30.5 (5.8)	136.0 (18.0)	76.7 (10.7)

NS – not stated; * CKD stages are given for the analysis population; Drug class: A – anti-hypertensive; L – lipid modifying; G – glycaemic control; B- sodium bicarbonate.

Table 6. Results of analyses

	Effect measure (95% CI)	No of studies or populations*	I^2
**1. Estimated Glomerular Filtration Rate**			
Antihypertensive drugs			
Random effects, DerSimonian and Laird method (Base Case)	1.03 (0.96 to 1.11)	9	95.3%
Fixed effects, inverse-variance method	1.01 (0.99 to 1.02)	9	95.3%
Hartung-Knapp-Sidik-Jonkman method	1.03 (0.93 to 1.14)	9	95.3%
Excluding studies with estimates	0.98 (0.94 to 1.03)	7	81.0%
Excluding studies with high risk of attrition or allocation bias	1.03 (0.95 to 1.13)	8	95.8%
Using CKD EPI data for CHARM trial	1.03 (0.96 to 1.11)	9	95.1%
More conservative imputation, using a larger SE	1.03 (0.97 to 1.10)	9	92.5%
Adding studies with CKD stage 1/2 patients	1.05 (0.99 to 1.11)	12	97.0%
Adding studies with CKD stage 1/2 patients and with placebo controls only	1.04 (0.98 to 1.10)	11	96.0%
Hartung-Knapp-Sidik-Jonkman method and studies with CKD stage 1/2 patients	1.05 (0.97 to 1.13)	12	97.0%
Lipid modifying drugs			
Random effects, DerSimonian and Laird method (Base Case)	1.04 (1.0001 to 1.08)	5	88.3%
Fixed effects, inverse-variance method	1.02 (1.01 to 1.03)	5	88.3%
Hartung-Knapp-Sidik-Jonkman method	1.04 (0.98 to 1.10)	5	88.3%
Excluding studies with non-placebo controls	1.05 (0.97 to 1.14)	3	93.8%
Excluding studies with estimates (SHARPb)	1.01 (0.99 to 1.03)	4	45.3%
Excluding studies with high risk of attrition or allocation bias	1.01 (1.003 to 1.02)	2	0%
More conservative imputation, using a larger SE	1.03 (0.999 to 1.06)	5	73.9%
Adding studies with CKD stage 1/2 patients	1.09 (1.03 to 1.15)	13	98.0%
Adding studies with CKD stage 1/2 patients and excluding studies with non-placebo controls	1.09 (1.004 to 1.18)	9	98.1%
Hartung-Knapp-Sidik-Jonkman method and adding studies with CKD stage 1/2 patients	1.09 (1.02 to 1.16)	13	98.0%
Glycaemic control drugs			
Random effects, DerSimonian and Laird method (Base Case)	1.06 (1.02 to 1.10)	6	0%
Fixed effects, inverse-variance method	No change		

Hartung-Knapp-Sidik-Jonkman method	1.06 (1.01 to 1.11)	6	0%
Excluding studies with estimates	0.96 (0.69 to 1.34)	2	0%
Excluding studies with high risk of attrition or allocation bias	No change		
More conservative imputation, using a larger SE	No change		
Adding studies with CKD stage 1/2 patients	0.94 (0.85 to 1.05)	11	85.1%
Hartung-Knapp-Sidik-Jonkman method and studies with CKD stage 1/2 patients	0.94 (0.80 to 1.12)	11	85.1%
CKD stage 1 to 3 data for EMPA-REG and adding studies with stage 1/2 patients	0.93 (0.82 to 1.04)	11	81.4%
Excluding Leiter 2016 (outlier) and Adding studies with CKD stage 1/2 patients	1.04 (1.01 to 1.07)	10	0%
Sodium bicarbonate			
Random effects, DerSimonian and Laird method	1.08 (0.84 to 1.38)	2	91.1%
**2. Proteinuria**			
Antihypertensive drugs			
Random effects, DerSimonian and Laird method (Base Case)	0.91 (0.82 to 1.01)	3	41.3%
Fixed effects to inverse-variance method	No change		
Excluding studies with estimates	0.80 (0.67 to 0.95)	1	-
Excluding studies of PER and PCR	0.98 (0.86 to 1.12)	2	0%
Excluding studies with high risk of attrition or allocation bias	No change		
More conservative imputation to using a larger SE	No change		
Adding studies with CKD stage 1/2 patients	0.74 (0.53 to 1.03)	5	94.5%
**3. Adverse events**			
Anti-hypertensive drugs			
HALT-PKD, CKD stages 3-4	0.89 (0.74 to 1.07)	1	-
Adding studies with CKD stage 1/2 patients	0.94 (0.83 to 1.05)	4	58.0%
Lipid modifying drugs			
Random effects, DerSimonian and Laird method (Base Case)	0.91 (0.78 to 1.06)	3	17.8%
Fixed effects to Mantel–Haenszel method	0.92 (0.86 to 0.98)	3	17.8%
Excluding studies with high risk of attrition or allocation bias	0.96 (0.52 to 1.79)	2	55.8%
Adding studies with CKD stage 1/2 patients	1.02 (0.79 to 1.32	5	55.3%
Glycaemic control drugs			

EMPA-REG	0.96 (0.94 to 0.99)	1	-
Adding studies with CKD stage 1/2 patients	1.01 (0.96 to 1.06)	6	62.7%
**4. Combined maintenance dialysis and kidney transplantation**			
Anti-hypertensive drugs			
HALT-PKD	0.69 (0.50 to 0.96)	1	-
Adding RENAAL (CKD stages 2 to 4)	0.75 (0.64 to 0.88)	2	0%
Lipid modifying drugs			
Random effects, DerSimonian and Laird method (Base Case)	0.97 (0.88 to 1.08)	3	0%
Fixed effects to Mantel–Haenszel method	No change		
Excluding studies with high risk of attrition or allocation bias	1.20 (0.34 to 4.25)	2	0%
Adding studies with stages 1-4 CKD	0.97 (0.88 to 1.07)	5	7.8%
Glycaemic control drugs			
EMPA-REG	0.78 (0.61 to 1.01)	1	-
Adding studies with CKD stage 1/2 patients	0.64 (0.27 to 1.53)	4	38.2%
**5. Cardiovascular events**			
Antihypertensive drugs			
Random effects, DerSimonian and Laird method (Base Case)	0.66 (0.33 to 1.34)	2 (with 1 excluded)	-
Fixed effects to Mantel–Haenszel method	No change		
Excluding studies with high risk of attrition or allocation bias	No change		
Adding studies with CKD stage 1/2 patients	0.92 (0.80 to 1.06)	4 (1 excluded)	0%
Lipid modifying drugs			
Random effects, DerSimonian and Laird method (Base Case)	0.64 (0.52 to 0.80)	5	29.6%
Fixed effects to Mantel–Haenszel method	0.65 (0.56 to 0.76)	5	29.6%
Excluding studies with high risk of attrition or allocation bias	0.66 (0.52 to 0.83)	4	35.1%
Excluding non-ITT study (AFCAPS/TEXCAPS)	0.66 (0.52 to 0.83)	4	35.1%
Adding studies with CKD stage 1/2 patients	0.60 (0.50 to 0.72)	11	59.0%
Glycaemic control drugs			
TECOS (CKD stage 3)	0.92 (0.79 to 1.08)	1	-
**6. Cardiovascular mortality**			
Antihypertensive drugs			
Random effects, DerSimonian and Laird method (Base Case)	0.79 (0.58 to 1.07)	2	0%
Fixed effects to Mantel–Haenszel method	0.78 (0.58 to 1.07)	2	0%

Taylor KS, *et al. BMJ Open* 2019; 9:e030596. doi: 10.1136/bmjopen-2019-030596

Excluding studies with high risk of attrition or allocation bias	No change		
Adding studies with CKD stage 1/2 patients	0.93 (0.80 to 1.08)	7	30.0%
Lipid modifying drugs			
Random effects, DerSimonian and Laird method (Base Case)	0.52 (0.30 to 0.92)	2	0%
Fixed effects to Mantel–Haenszel method	0.52 (0.29 to 0.91)	2	0%
Excluding studies with high risk of attrition or allocation bias	0.53 (0.30 to 0.93)	1	-
Excluding non-ITT study (AFCAPS/TEXCAPS)	0.53 (0.30 to 0.93)	1	-
Adding studies with CKD stage 1/2 patients	0.58 (0.35 to 0.92)	5	0%
Glycaemic control drugs			
Random effects, DerSimonian and Laird method (Base Case)	0.74 (0.53 to 1.02)	2	77.8%
Fixed effects to Mantel–Haenszel method	0.74 (0.63 to 0.86)	2	77.8%
Excluding studies with high risk of attrition or allocation bias	No change		
Adding studies with CKD stage 1/2 patients	0.73 (0.57 to 0.93)	4	37.2%
**7. All-cause mortality**			
Antihypertensive drugs			
Random effects, DerSimonian and Laird method (Base Case)	0.93 (0.81 to 1.08)	3	0%
Fixed effects to Mantel–Haenszel method	0.92 (0.80 to 1.07)	3	0%
Excluding studies with high risk of attrition or allocation bias	No change		
Adding studies with CKD stage 1/2 patients	0.94 (0.87 to 1.02)	8	31.0%
Lipid modifying drugs			
Random effects, DerSimonian and Laird method (Base Case)	0.74 (0.56 to 0.98)	4	0%
Fixed effects to Mantel–Haenszel method	0.72 (0.54 to 0.95)	4	0%
Excluding studies with high risk of attrition or allocation bias	No change		
Adding studies with CKD stage 1/2 patients	0.74 (0.59 to 0.92)	9	24.6%
Glycaemic control drugs			
Random effects, DerSimonian and Laird method (Base Case)	0.88 (0.74 to 1.04)	2	32.2%
Fixed effects to Mantel–Haenszel method	No change		
Excluding studies with high risk of attrition or allocation bias	No change		
Adding studies with CKD stage 1/2 patients	0.86 (0.73 to 1.02)	7	0%

Table 7. GRADE assessments of primary outcome for each drug group

Effects of antihypertensives. lipid-modifying drugs, glacaemic control drugs and sodium bicarbonate on the progression of stages 3 and 4 chronic kidney disease in adults

Patient or population: patients with chronic kidney disease (CKD)
Settings: primary care and shared care with specialist nephrology services
Intervention: drug
Comparison: placebo drug, no drug intervention, or a comparator drug from the three other classes

Outcomes	Illustrative comparative risks* (95% CI)		Relative effect (95% CI)	No of Participants (studies)	Quality of the evidence (GRADE)	Comments
	Assumed risk	Corresponding risk				
	**Placebo drug, no drug intervention, or a comparator drug from the three other classes**	**Drug**				
**eGFR (antihypertensives)** Follow-up: 24 to 62 months	See comment	See comment		5433 (7 studies[1])	⊕⊖⊖⊖ **very low**[2,3,4,5,6]	Ratio of means 1.03, 95% CI 0.96 to 1.11[7]
**eGFR (lipid-modifying drugs)** Follow-up: 24 to 64 months	See comment	See comment		6865 (4 studies[8])	⊕⊖⊖⊖ **very low**[4,6,9,10]	Ratio of means 1.04, 95% CI 1.00 to 1.08[7,11]
**eGFR (glycaemic control drugs)** Follow-up: 29 to 36 months	See comment	See comment		1438 (3 studies[12])	⊕⊕⊖⊖ **low**[4,13,14]	Ratio of means 1.06, 95% CI 1.02 to 1.10 [7]
**eGFR (sodium bicarbonate)** Follow-up: 24 to 36 months	See comment	See comment		180 (2 studies)	⊕⊖⊖⊖ **very low**[4,14,15,16,17]	Ratio of means 1.0, 95% CI 0.84 to 1.38 [7,18]

*The basis for the **assumed risk** (e.g. the median control group risk across studies) is provided in footnotes. The **corresponding risk** (and its 95% confidence interval) is based on the assumed risk in the comparison group and the **relative effect** of the intervention (and its 95% CI).

CI: Confidence interval;

GRADE Working Group grades of evidence
High quality: Further research is very unlikely to change our confidence in the estimate of effect.
Moderate quality: Further research is likely to have an important impact on our confidence in the estimate of effect and may change the estimate.
Low quality: Further research is very likely to have an important impact on our confidence in the estimate of effect and is likely to change the estimate.
Very low quality: We are very uncertain about the estimate.

[1] 9 datasets
[2] Risk of bias: Only one study was high risk both for incomplete outcome data and selective reporting. For all other risk of bias categories studies were assessed as low or unclear risk of bias.
[3] Inconsistency: there is a wide variation in study and participant characteristics. Point estimates for studies both indicated drug increasing and decreasing progression of CKD. Minimal overlap of confidence intervals. Substantial heterogeneity (I-squared 95.3%, p-value 0.000).
[4] Indirectness: Effect of drug on CKD was secondary to specified study outcomes.
[5] Imprecision: 95% confidence interval includes no effect
[6] Publication bias: few studies in the area of non-significance (contour enhanced funnel plots). Note: funnel plots also included studies which had populations mixed with stage 1 and 2 CKD.
[7] Weighted average of the mean eGFR in the intervention group divided by the mean eGFR in the control group. Ratio of means >1 favoured the intervention group for eGFR.

[8] 5 datasets
[9] Risk of bias: one study was high risk for blinding of participants, personnel and outcome assessment, One study (two datasets) was high risk of bias for incomplete outcome data. For all other risk of bias categories studies were assessed as low or unclear.
[10] Inconsistency: there a wide variation in study and participant characteristics. Point estimates, bar one study, show drug decreasing the progression of CKD. Substantial heterogeneity (I-squared 88.3%, p-value 0.000).
[11] STATA's metaninf command produced output that indicated that the lower confidence bound was 1.0001 to 4 d.p.
[12] 6 datasets
[13] Risk of bias: one study was high risk for selective reporting. For all other risk of bias categories studies were assessed predominantly as low.
[14] Publication bias: insufficient data to assess.
[15] Risk of bias: both studies were high risk for blinding, plus one study for allocation concealment. For all other risk of bias categories studies were assessed as low or unclear.
[16] Inconsistency: there is wide variation in study and participant characteristics. The point estimates of the two studies were either side of the line of no effect. No overlap of confidence intervals. Substantial heterogeneity (I-squared 91.1%, p-value 0.000).
[17] Imprecision: the total sample size is less than 300. 95% confidence interval includes no effect.
[18] Data pooled, though sparse, to provide an overall indication of treatment effect and allow comparison between drug classes

Supplementary Figures

Figure 1. Kidney Disease Improving Global outcomes (KDIGO) 2012

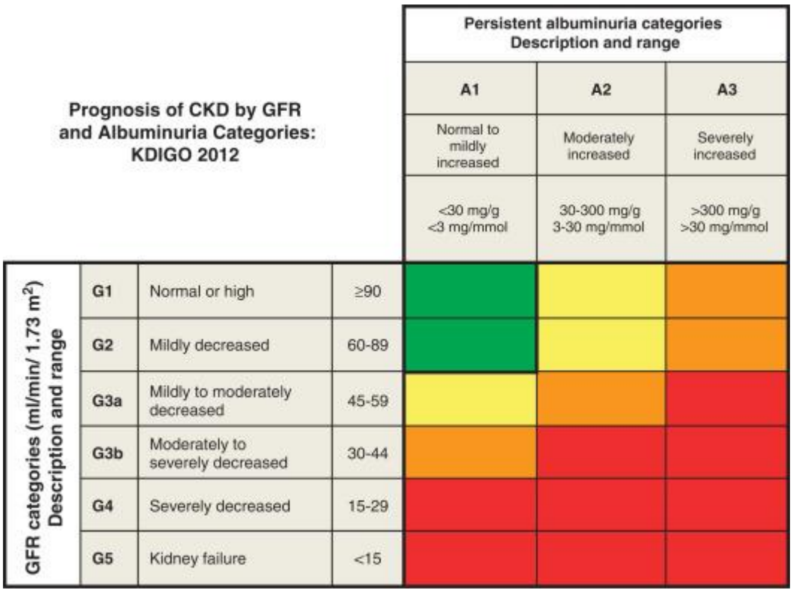

Albuminuria = proteinuria. Green: low risk (if no other markers of disease, no CKD); yellow moderately increased risk; orange: high risk; red: very high risk

(Reproduced with permission)

Figure 2. Cochrane Collaboration's Risk of bias

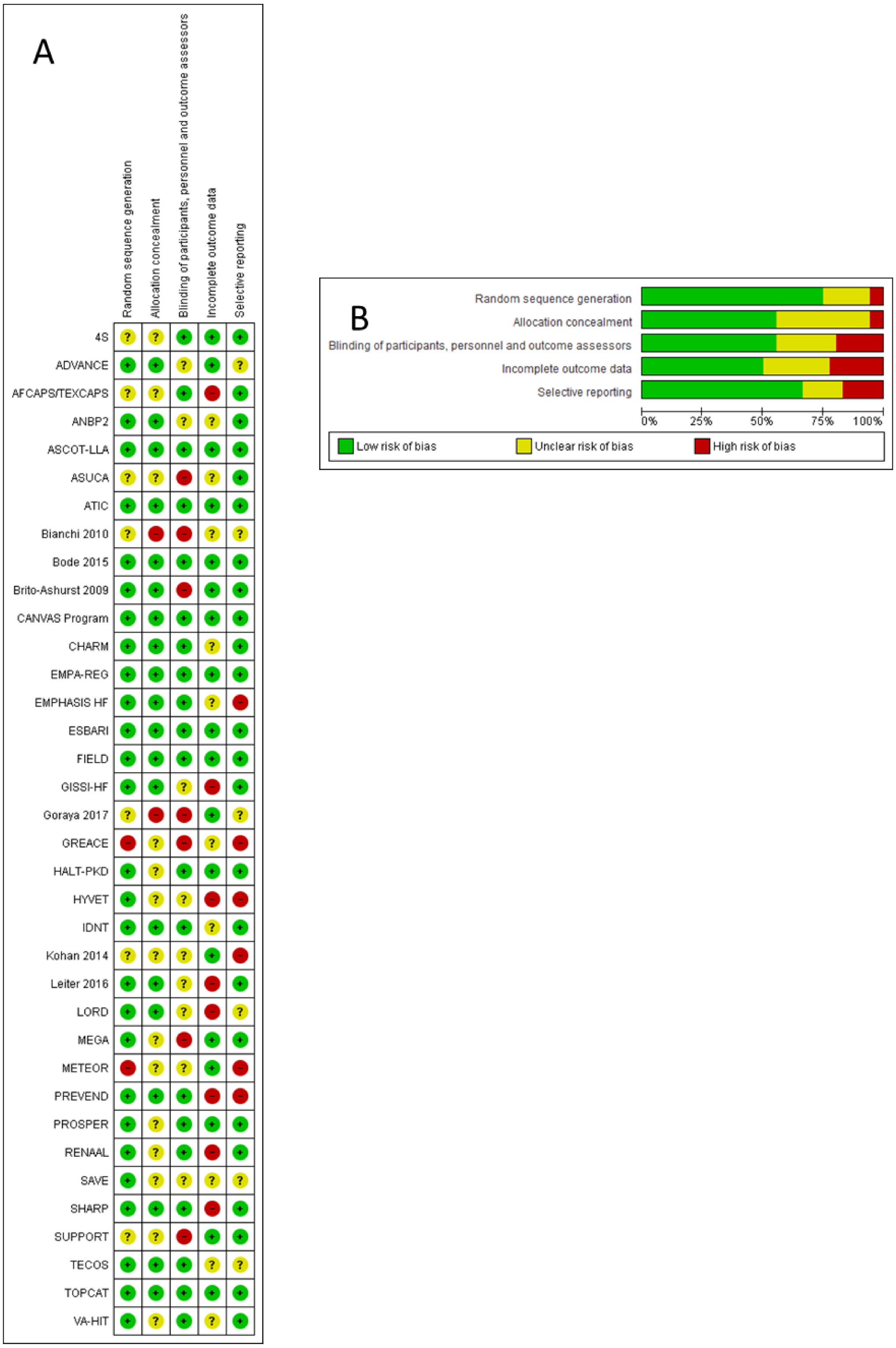

Taylor KS, *et al. BMJ Open* 2019; 9:e030596. doi: 10.1136/bmjopen-2019-030596

Figure 3. Funnel plots and contour enhanced funnel plots, for estimated glomerular filtration rate data, split by drug group

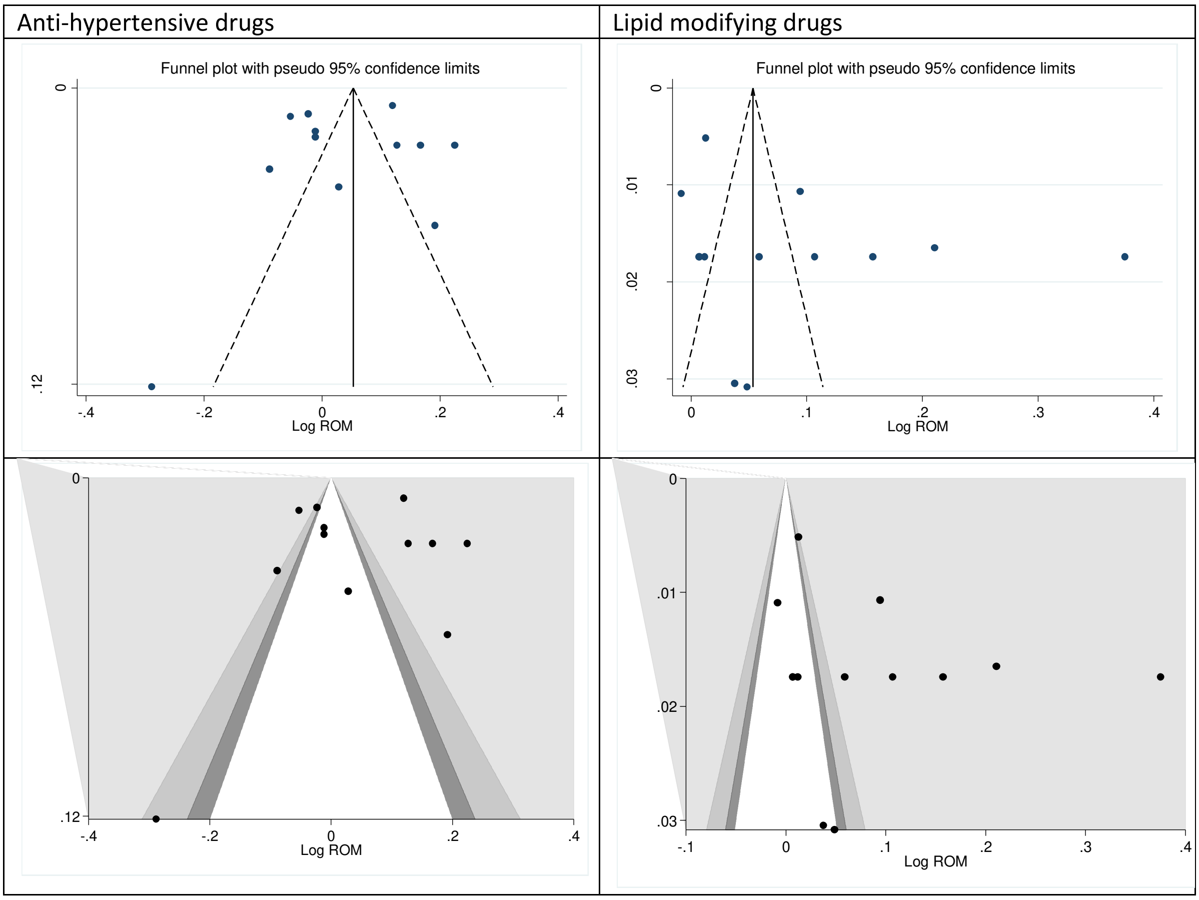

A positive Log ROM favours the intervention drug. Log refers to the natural logarithm.

Contour plots shading: light grey (p<1%); medium grey (1%<p<5%); dark grey (5%<p<10%); white (p>10%)

Figure 4. Ratio of means of estimated glomerular filtration rate at the end of the trials for anti-hypertensives vs comparator (boxes) and pooled estimates across studies (diamonds) calculated by the random effects DerSimonian and Laird method, for chronic kidney disease stages 3 and 4 and split by drug class

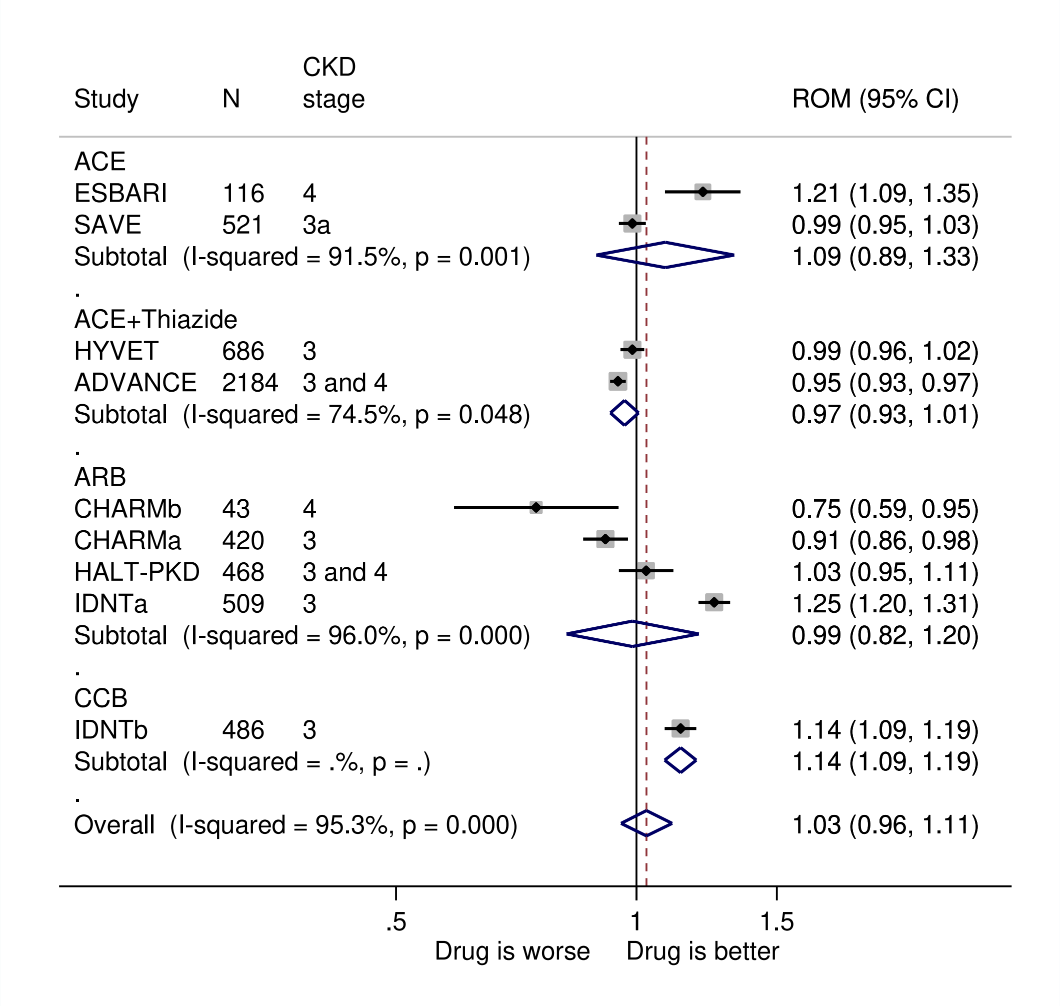

CKD – chronic kidney disease; CHARMa and CHARMb – CKD stage 3 and 4 respectively with eGFR measured by MDRD equation; IDNTa - intervention is Irbesartan; IDNTb - intervention is Amlodipine.

Taylor KS, *et al. BMJ Open* 2019; 9:e030596. doi: 10.1136/bmjopen-2019-030596

Figure 5. Ratio of means of proteinuria at the end of the trials for anti-hypertensives vs comparator (boxes) and pooled estimates across studies (diamonds) calculated by the random effects DerSimonian and Laird method, split by chronic kidney disease stage

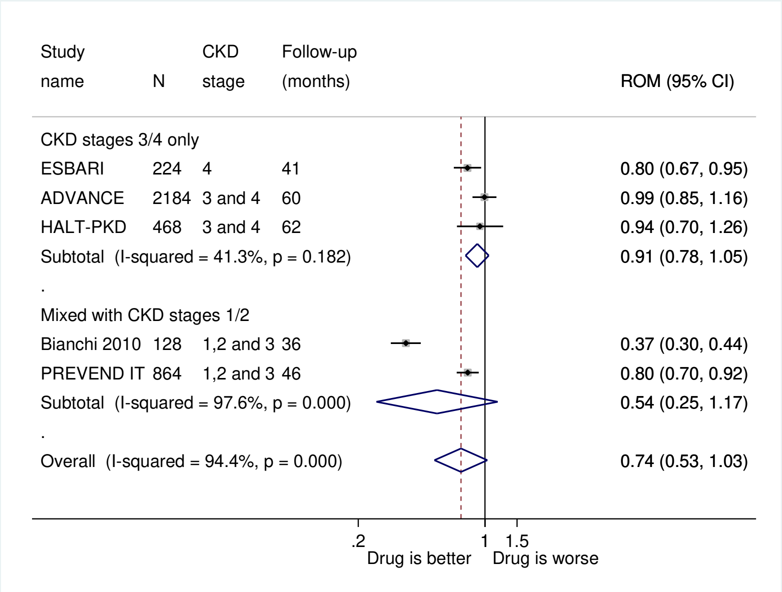

CKD – chronic kidney disease. Prediction interval for overall (0.20 to 2.70).

Taylor KS, *et al. BMJ Open* 2019; 9:e030596. doi: 10.1136/bmjopen-2019-030596
