# Supplementary material for: Effects of antihypertensives, lipid-modifying drugs, glycaemic control drugs and sodium bicarbonate on the progression of stages 3 and 4 chronic kidney disease in adults: a systematic review and meta-analysis
Source: BMJ Open. 2019 Sep 20;9(9):e030596. doi: 10.1136/bmjopen-2019-030596 (PMC6756484; doi:10.1136/bmjopen-2019-030596)
Supplement: Author's manuscript [file bmjopen-2019-030596.draft_revisions.pdf]

BMJ Open

BMJ Open is committed to open peer review. As part of this commitment we make the peer review history of every article we publish publicly available.

When an article is published we post the peer reviewers' comments and the authors' responses online. We also post the versions of the paper that were used during peer review. These are the versions that the peer review comments apply to.

The versions of the paper that follow are the versions that were submitted during the peer review process. They are not the versions of record or the final published versions. They should not be cited or distributed as the published version of this manuscript.

BMJ Open is an open access journal and the full, final, typeset and author-corrected version of record of the manuscript is available on our site with no access controls, subscription charges or pay-per-view fees (http://bmjopen.bmj.com).

If you have any questions on BMJ Open's open peer review process please email info.bmjopen@bmj.com

BMJ Open

Effects of antihypertensives, lipid-modifying drugs, glycaemic control drugs and sodium bicarbonate on the progression of stages 3 and 4 chronic kidney disease in adults: a systematic review and meta-analysis

Journal:	*BMJ Open*
Manuscript ID	bmjopen-2019-030596
Article Type:	Research
Date Submitted by the Author:	21-Mar-2019

Complete List of Authors:	Taylor, Kathryn; University of Oxford, Nuffield Department of Primary Care Health Sciences<br>Mclellan, Julie; University of Oxford, Nuffield Department of Primary Care Health Sciences<br>Verbakel, Jan; University of Oxford, Nuffield Department of Primary Care Health Sciences; KU Leuven, Department of Public Health and Primary Care<br>Aronson, Jeffrey; University of Oxford, Nuffield Department of Primary Care Health Sciences<br>Lasserson, Daniel; University of Birmingham, Institute of Applied Health Research; University of Oxford, Nuffield Department of Medicine<br>Pidduck, Nicola; University of Oxford, Nuffield Department of Primary Care Health Sciences<br>Roberts, Nia; University of Oxford, Bodleian Health Care Libraries<br>Fleming, Susannah; University of Oxford, Nuffield Department of Primary Care Health Sciences<br>O'Callaghan, Christopher; University of Oxford, Nuffield Department of Clinical Medicine; Oxford Radcliffe Hospitals Trust, John Radcliffe Hospital<br>Bankhead, Clare; University of Oxford, Nuffield Department of Primary Care Health Sciences<br>Banerjee, Amitava; University College London, Farr Institute of Health Informatics Research<br>Hobbs, Richard; University of Oxford, Nuffield Department of Primary Care Health Sciences<br>Perera, Rafael; University of Oxford, Nuffield Department of Primary Care Health Sciences

Keywords:	Chronic renal failure < NEPHROLOGY, Cardiovascular events, Meta-analysis, Renal function, Drug treatment

SCHOLARONE™
Manuscripts

Effects of medicines on the progression of stages 3 and 4 chronic kidney disease in adults: a systematic review and meta-analysis

Kathryn S Taylor[1], Julie McLellan[1], Jan Y Verbakel[1,2], Jeffrey Aronson[1], Daniel S Lasserson[3], Nicola Pidduck[1], Nia Roberts, Susannah Fleming[1], Christopher A O'Callaghan[4], Clare Bankhead[1], Amitava Banerjee[5], F.D. Richard Hobbs[1], Rafael Perera[1].

[1] Nuffield Department of Primary Care Health Sciences, University of Oxford, OX2 6GG, United Kingdom

[2]Department of Public Health and Primary Care, KU Leuven, Belgium

[3] Institute of Applied Health Research, College of Medical and Dental Sciences, Murray Learning Centre, University of Birmingham, Edgbaston, Birmingham, B15 2TT, United Kingdom.

[4]Nuffield Department of Medicine, University of Oxford, Henry Wellcome Building, Roosevelt Drive, Oxford OX3 7BN, United Kingdom

[5] Health Data Research UK-London, Institute of Health Informatics, University College London, 222 Euston Road London, NW1 2DA, United Kingdom

Kathryn S Taylor, Medical Statistician

Julie McLellan, Senior Systematic Reviewer

Jan Verbakel, Senior Clinical Researcher and Tenure Track Professor

Jeffrey Aronson, Consultant Physician and Clinical Pharmacologist

Daniel Lasserson, Professor of Ambulatory Care

Nicola Pidduck, Project Manager and Systematic Reviewer

For peer review only

Nia Roberts, Librarian

Susannah Fleming, Quantitative Researcher

Christopher A O'Callaghan, Professor of Medicine and Honorary Consultant Physician and Nephrologist

Amitava Banerjee, Senior Clinical Lecturer in Clinical Data Science and Honorary Consultant Cardiologist

F.D. Richard Hobbs, Professor of Primary Care Health Sciences

Rafael Perera, Professor of Medical Statistics

Correspondence to: Julie McLellan, Nuffield Department of Primary Care Health Sciences, University of Oxford, OX2 6GG, United Kingdom, julie.mclellan@phc.ox.ac.uk, 01865 289667

Word Count: 4713

For peer review only - http://bmjopen.bmj.com/site/about/guidelines.xhtml

ABSTRACT

Objectives: To evaluate the effects of drug interventions that may modify the progression of chronic kidney disease (CKD) in adults with CKD stages 3 and 4.

Design: Systematic review and meta-analysis.

Data sources: MEDLINE and 10 other databases, to 6 July 2018.

Eligibility criteria: Randomised controlled trials of drugs for hypertension, lipid modification, glycaemic control, and sodium bicarbonate, compared with placebo, no drug intervention, or a drug from a different drug class. Adults with CKD stage 3 and/or stage 4, with/without patients with CKD stage 1 and/or stage 2. Studies of ≥40 people with ≥two years of follow-up reporting renal function (primary outcome), proteinuria, adverse events, maintenance dialysis, transplantation, cardiovascular events, cardiovascular mortality or all-cause mortality.

Data extraction and synthesis: Two reviewers independently screened citations and extracted data. For continuous outcomes, we used random-effects meta-analyses to estimate the effect measure based on a ratio of means (ROM) at the end of the trial.

Results: We included 35 studies and over 51,000 patients. Data were limited. Heterogeneity varied. In 19 studies about patients with CKD stages 3 and 4 only, renal function at the end of the study, measured by the estimated glomerular filtration rate (eGFR), was 6% higher in those taking glycaemic control drugs (ROM 1.06, 95% CI 1.02 to 1.10, I^2=0%) and 4% higher in those taking lipid-modifying drugs (ROM 1.04, 1.00 to 1.08, I^2=88%). For the studies of antihypertensive drugs, there were no significant differences in renal function. Treatment with lipid-modifying drugs led to a 36% reduction in cardiovascular disease and 26% reduction in all-cause mortality.

For peer review only - http://bmjopen.bmj.com/site/about/guidelines.xhtml

Conclusions: Glycaemic control and lipid-modifying drugs may slow the progression of CKD, but we found no evidence of benefit nor harm from antihypertensive drugs. However, given the data limitations, further research is needed to confirm these findings.

PORSPERO registration: CRD42015017501

ARTICLE SUMMARY

Strengths and limitations of this study

- By focusing on patients with CKD stage 3 and 4, we provide a primary care perspective on the management of CKD.
- We completed an extensive and comprehensive search including common drug misspellings, with no restriction on language or publication type.
- Generalisability of our findings is limited by the paucity of trials, high statistical heterogeneity, high clinical heterogeneity, in terms of co-morbidities, and by the effect of drugs on CKD being secondary in all the studies.
- Lack of data restricted our ability to explore the baseline characteristics of studies, the effects of drugs, the link between treatment effect and drug dose, and assess the potential for publication bias.

For peer review only

For peer review only - http://bmjopen.bmj.com/site/about/guidelines.xhtml

INTRODUCTION

Chronic kidney disease (CKD) is long-term loss of renal function and is a global health problem, with an estimated prevalence of 11-13%.[1] CKD is an independent risk factor for cardiovascular disease,[2] and people with mild-to-moderate CKD are at increased risk of mortality, and progression to renal failure.[3]

Historically, CKD was categorised by its cause, which was predominantly diabetes, hypertension, glomerulonephritis, or polycystic kidney disease. Treatment could be recommended, but often opportunities were missed for early detection of the disease or prevention of clinical complications and further deterioration. The National Kidney Foundation, addressed this challenge by developing a five-stage classification system based on the glomerular filtration rate (GFR) (Table S1), [4] which was subsequently modified to take into account degrees of proteinuria (Figure S1).[5] A UK audit in 2012 estimated that 90% of cases of CKD are at stage 3, and of these, 84% are stage 3a (eGFR 45-59 ml/min/1.73^2).[6]

Patients with CKD who are managed in primary care include those in stages 1 and 2, with an emphasis on lifestyle and diet changes. Patients in stages 3 and 4 may be managed either in primary care or shared care with specialist nephrology services, with a greater use of medication, primarily for associated long-term conditions, such as diabetes and hypertension. Both published data for Oxfordshire[7] and unpublished national data from our group show that some patients with CKD stage 4 have their renal function monitored in primary care.

Four different classes of drugs may modify CKD progression: antihypertensives,[8] lipid-modifying drugs,[9] glycaemic control medications in patients with diabetes,[10] and sodium bicarbonate.[11-13] Sodium bicarbonate addresses metabolic acidosis, a prevalent complication in moderate and late-stage CKD, and may offer a simple and low-cost treatment to slow CKD progression. The effects of

For peer review only - http://bmjopen.bmj.com/site/about/guidelines.xhtml

drugs on CKD are secondary, as these drugs are primarily used to treat hypertension, hypercholesterolemia, or diabetes.

Systematic reviews have been compiled to summarise the effects of drug interventions on the rate of CKD progression.[9 10 14-16] However, while two have reported results based on estimated GFR (eGFR), none have reported results with reference to CKD staging. Furthermore, no systematic review has compared the effects of different drug classes.

Our objective was to carry out a systematic review and meta-analysis of randomised controlled trials, to examine and compare the effects on the progression of CKD of the four classes of drugs, focusing on patients who can be managed in primary care or shared care with specialist nephrology services.

METHODS

Our systematic review was registered and conducted in line with recommendations for systematic reviews and meta-analyses (PRISMA[17]).

Search strategy

The search strategy was designed with advice from an information specialist (NR) and clinicians including nephrologists. MeSH terms included terms for population, study design, and intervention drugs (randomised control trials, chronic kidney, renal, drug classes, and specific drug names, including common misspellings).

Searches were conducted in Ovid MEDLINE (File S1), the Cochrane Register of Controlled Trials, the Cochrane Database of Systematic Reviews, Database of Abstracts of Reviews of Effects, Embase,

For peer review only - http://bmjopen.bmj.com/site/about/guidelines.xhtml

For peer review only

Health Technology Assessment, Science Citation Index, Social Science Citation Index, Conference Proceedings Citation Index, Clinical Trials Register, and the World Health Organization International Clinical Trials Platform to 6 July 2018 to identify relevant studies, regardless of their publication type. No language restriction was applied. All publications from March 1999 onwards were considered, to align with the publication of the first equation to calculate eGFR, which had led to the introduction of CKD staging.[18] Reference lists were also examined.

Study selection

Two reviewers (KST, JM, NP and SF; in pairs) reviewed the title and abstract of each reference and identified potentially relevant references. Considering the full texts of these studies, two reviewers independently selected studies to be included in the review using predetermined inclusion criteria. Disagreements about study inclusion were resolved by a third reviewer.

Our study inclusion criteria were:

(1) Randomised controlled trials of drugs that may modify the progression of CKD, antihypertensives, lipid-modifying drugs, glycaemic control drugs, and sodium bicarbonate
(2) Control arms were either given a placebo drug, no drug intervention, or a comparator drug from the three other classes
(3) Adult participants
(4) Patients with stage 3 and/or stage 4 CKD. Studies additionally including patients with stage 1 and/or stage 2 CKD were also included to increase the number of potential studies, but we would expect the treatment effect to be overestimated in these studies, as in very advanced disease, the response to treatment may be attenuated by the severity of associated physiological changes.
(5) Populations with at least 40 participants

For peer review only - http://bmjopen.bmj.com/site/about/guidelines.xhtml

(6) Follow-up of at least two years

(7) Publications reporting a quantitative summary of effect as a change from baseline, or an endpoint measure of at least one of seven pre-specified outcomes

i. Renal function, which could be measured by GFR, eGFR, creatinine clearance (CrCl) or estimated CrCl (eCrCl). This was our primary outcome of interest.

ii. Proteinuria, which could be measured by protein excretion rate (PER), protein creatinine ratio (PCR), albumin excretion rate (AER) or albumin creatinine ratio (ACR).

iii. Adverse events

iv. Commencement of maintenance dialysis or kidney transplantation

v. Cardiovascular events

vi. Cardiovascular mortality

vii. All-cause mortality

We excluded trials that reported only data about populations that included people with CKD stage 5, to avoid underestimating the treatment effect, as we would expect stage 5 patients to have more co-morbidities, which might lead to worse outcomes.

Data extraction and quality assessment

Data were extracted independently by two reviewers (KST, JM, NP and JV; in pairs) using a data extraction form, which was piloted on a sample of five studies.

Extracted data included study details (setting, intervention dose and frequency, follow-up period) information on study participants (age, gender, ethnicity, smoking status, blood pressure, CKD stage, existing co-morbidities) and outcome data. For one study[19 20] we extracted endpoint eGFR data from an earlier point than the longest follow-up as the patient numbers at the longest follow-up were so

For peer review only - http://bmjopen.bmj.com/site/about/guidelines.xhtml

small (<7% of the population). We contacted study authors to seek clarification about data and to request data that could be included in our study.

The methodological quality of studies was assessed independently by two reviewers (KST, JM, NP and JV; in pairs) using the Cochrane Risk of Bias tool.[21] This tool considers the method of random sequence generation and allocation concealment (selection bias), blinding of participants, personnel and outcomes (performance and detection biases), the levels and balance of missing outcome data (attrition bias), and completeness of reporting (selective reporting bias).

Disagreements about extracted data or methodological assessments were resolved by a third reviewer.

Data synthesis and analysis

We expected high statistical heterogeneity,[22] but pooled data to give an indication of the average effect. We also pooled data when there were only 2 or 3 studies, although this could be potentially misleading, in order to give an indication of potential trends had there been more study data. We carried out random-effects analyses based on the DerSimonian and Laird method[23] and assessed statistical heterogeneity using the I-squared statistic and calculated approximate 95% prediction intervals to estimate the likely effects in a clinical setting[24] using the methods of Higgins et al.[25]

For the continuous outcomes (renal function and proteinuria), we used a ratio of means (ROM)[26 27] effect measure, which allowed us to combine data from trials that reported different measures of renal function or proteinuria in a single analysis. We calculated the ROM as the ratio of the final eGFR in the intervention group to the final eGFR in the comparator group, so ROM>1 favoured the intervention group for eGFR, where higher values are better than lower values. If a study of renal

For peer review only - http://bmjopen.bmj.com/site/about/guidelines.xhtml

function reported reductions from baseline or rates of decline rather than endpoint data, we estimated the ROM of endpoints by the ratio of the reduction from baseline or rate of decline in the comparator group to that in the intervention group (the reciprocal) to ensure the direction of effect was consistent across all studies, as higher values of declining renal function are worse. ROM<1 favoured the intervention group for the proteinuria outcomes (ACR, AER, PER, PCR), where higher values are worse.

For a few studies it was necessary to make further estimates by: approximating a mean using the median; estimating a standard deviation from the interquartile range;[21] or estimating the standard error of the ROM by imputation using the mean value of the known standard errors of the other studies.

Meta-analysis was carried out on the log-transformed ROM and its standard error. For the primary analysis, eGFR measured by the Modification of Diet in Renal Disease (MDRD) equation was selected over Chronic Kidney Disease Epidemiology Collaboration (CKD-EPI) data, as MDRD is the main equation advocated in clinical guidelines and CKD-EPI was not introduced until 2009;[5 28] eGFR was also selected over CrCl. When analysing the effects of treatments on proteinuria we pooled data for PER, PCR, ACR, and AER by using a ROM effect measure. For the dichotomous outcomes, we specified the relative risk as the effect measure.

We performed separate sensitivity analyses to ensure that our estimates of the effect of treatment in studies of patients with CKD stages 3 and 4 only (our main focus) were not sensitive to our choice of model, assumptions, the quality of the studies, the estimations made, or our choices of data:

1) Fixed-effect analyses, based on the inverse-variance method were carried out for the continuous outcomes, and based on the Mantel–Haenszel method for the dichotomous outcomes.[29 30]

For peer review only - http://bmjopen.bmj.com/site/about/guidelines.xhtml

2) As a post-hoc analysis of our primary outcome, we used the Hartung-Knapp-Sidik-Jonkman variance correction to calculate 95% confidence intervals reflecting the uncertainty in heterogeneity estimates.[31-33]
3) Excluding studies that did not report intention-to-treat analysis.
4) Excluding studies with estimates.
5) Excluding studies rated as low quality, which we defined as having a high risk of attrition bias or allocation concealment bias; studies rated as 'unclear' were not excluded.
6) Using more conservative estimates of the standard error of the ROM by imputing with the 75% percentile value of the known standard errors rather than the mean value, reflecting greater uncertainty.
7) Using CKD-EPI data instead of MDRD data, for studies that measured eGFR using both equations.
8) Excluding studies that measured PCR and PER in the analysis of proteinuria, as urine albumin is a more accurate measure of glomerular damage.
9) Excluding each individual study, one-by-one, to see if the level of heterogeneity depended on a particular study.

We pre-specified subgroup analyses by age (<65 or ≥65 years), ethnicity, smoking status, intensity of intervention, blood pressure (high or low), method of eGFR measurement, degree of proteinuria in the renal function analysis, drug type within each drug class, and by CKD stages 3 and 4 separately. Heterogeneity was assessed using the I^2 statistic. Publication bias was explored using funnel plot analysis with contour plots.[21 34]

When describing the included trials, we refer to "studies" to encompass single trials and publications that report more than one trial. When reporting analyses of the effects of the drugs, we refer to populations as some studies reported data for CKD subgroups or dose-dependent subgroups. All

For peer review only - http://bmjopen.bmj.com/site/about/guidelines.xhtml

For peer review only

results of meta-analysis are displayed in forest plots and/or tabulated. Results are divided into those for studies of patients with CKD stages 3 and 4 only and studies of patients with CKD stages 3 and 4 mixed with patients with stages 1 and 2.

All analyses were carried out using STATA v14.2[35] except the risk of bias figures which were created in RevMan v 5.3.[36]

Patient involvement

Members of a patient and public involvement group were part of the Stakeholder Group and Steering Committee of the National Institute for Health Research (NIHR) Programme Grant that inspired this study. No patients or patient representatives were involved in setting the outcome measures, nor were they directly involved in developing plans for the design or implementation of the study. Three members of this group commented on our manuscript and we thank them for their help in our acknowledgement section. A one-day dissemination event is planned, to report the results of all the studies funded by the NIHR Programme Grant, including this study. Members of the patient and public involvement group will be invited to this event.

RESULTS

Search results

Through database registry and hand searches we identified 13,133 records (Figure 1), of which 35 studies (51,155 patients) satisfied our eligibility criteria.

Study characteristics

For peer review only - http://bmjopen.bmj.com/site/about/guidelines.xhtml

Most of the 35 studies reported data about a single trial while three studies reported data about a pooled analysis of more than one trial.[19 20 37-42] Of the 35 studies, one from China,[43] one from Tasmania,[44] three from Japan,[45-50] six from the USA/Canada,[51-64] nine from Europe,[13 65-77] either in single or multiple countries, and the other 15 studies were multinational (Table S2). Nineteen studies[13 19 20 37-39 43 47-56 60-64 67-69 73 74 77-89] provided data for populations with CKD stages 3 and/or 4 only. These studies had between 108 and 3094 patients and the mean or median follow-up was between 24 and 66 months.

One study[66] had a 2x2 factorial design involving an antihypertensive drug and a lipid-modifying drug and this study is included in our analysis of both antihypertensive drugs and lipid-modifying drugs. Another study had a 2x2[70 71] factorial design involving two antihypertensive drugs and a lipid-modifying drug, and this study is only included in our analysis of lipid-modifying drugs because all patients received antihypertensives. The other 33 studies were 12 studies of antihypertensives,[16 37-39 43 45 46 51-56 65 66 78-82 90-97] 13 of lipid-modifying drugs,[44 47-50 57-62 67-69 72-77 83-85 98-103] six of glycaemic control agents[19 20 40-42 86-89 104-106] and two of sodium bicarbonate[13 63 64] (Table S3). Four studies[44 57-59 72 101-103] had populations with CKD and no comorbidities highlighted, 27 studies had a single co-morbidity and four studies had more than one comorbidity. Co-morbidities were cardiovascular in 19 studies;[19 20 37-42 45-50 52 53 56 60-62 67-71 73-77 80-82 88-91 97-100] type II diabetes in nine studies[19 20 40-42 52-55 78 79 86-89 92-96 104-106] (including all six studies of glycaemic control drugs); and a renal disorder or damage (idiopathic chronic glomerulonephritis, advanced chronic renal insufficiency without diabetes, advanced autosomal dominant polycystic kidney disease, renal impairment and metabolic acidosis) in seven studies[43 51 63-66 83-85 107] (Table S3). In 15 studies,[13 44-46 60-66 70-72 75 76 90-98 106] the population analysed for our primary outcome was the whole trial population and in the other 20 the population analysed was a subgroup of the trial population, reported as either a subgroup or post-hoc analysis. (Table S2).

For peer review only

For peer review only - http://bmjopen.bmj.com/site/about/guidelines.xhtml

Baseline data were provided for the same population as that included in our analyses for 19 of the 35 studies;[13 44 48-50 57-69 72 75-79 83-87 90-97 106] for the other 16 studies, baseline data were only provided for either a different subset of the trial population or the whole trial population (Table S4).

Risk of bias

We considered the methodological quality of all 35 eligible studies (Figure S2), based on the design of the trial(s) from which data were obtained. Eight studies[19 20 43 70-72 83-87 97 104 105] were ranked with high quality as they had 'Low' risk of bias across all domains and we rated one study[75 76] as 'High' risk of bias for three domains and 'Unclear' across two domains. Most studies described their method of randomisation, but fewer studies were clear about how they concealed the group allocation and whether participants, personnel and outcome assessors were blinded. The risk of attrition bias was high in nearly a quarter of the studies.

We had sufficient data to consider the possibility of publication bias based on the primary outcome of renal function in those taking antihypertensive and lipid-modifying drugs. The contour-enhanced funnel plots suggested possible publication bias, as few studies were in the area of non-significance (Figure S3).

Renal function

Most of the available data were on eGFR.

The ratio of means of eGFR for antihypertensive drugs versus comparators at the end of the studies is shown in Figure 2. There was no significant effect in studies of populations with CKD stages 3 and 4, and heterogeneity between studies was substantial at 94% (yielding a prediction interval of 0.79 to

For peer review only - http://bmjopen.bmj.com/site/about/guidelines.xhtml

1.34) and this was not attributable to a single study. Adding data from the studies of patients that included those with CKD stages 1 and/or 2 increased the benefit to the intervention group but not significantly. All the sensitivity analyses produced similar results of no significant treatment effect and heterogeneity remained substantial (Table S5).

The only subgroup analysis that was possible, of those pre-specified, was by drug type within the antihypertensive drug class. Among studies of CKD stages 3 and 4 only, there were no significant treatment effects within the different drugs sub-classes of antihypertensive drugs (Figure S4), except in the case of calcium channel blockers, where the endpoint eGFR was 14% higher in the intervention group (ROM 1.14, 95% confidence interval 1.01 to 1.27), but this was based on a single study.[52 53]

In studies of patients with CKD stages 3 and 4 there was a marginally higher benefit to those taking the lipid-modifying drugs (Figure 3), as the endpoint eGFR was 4% higher (ROM 1.04, 1.00 to 1.08, statistically significant, I^2=88, STATA's metaninf command produced output that indicated that the lower confidence bound was 1.0001 to 4 d.p.) with a prediction interval of 0.91 to 1.18. This result was not robust as the results of sensitivity analysis were variable (Table S5). The high heterogeneity was attributed to the SHARP study[101-103] of the CKD stage 4 subgroup. Adding data from the studies of patients including those with CKD stages 1 and/or 2 increased the benefit to the intervention group but not significantly and there was substantial heterogeneity between studies (I^2=98%).

There was a significant benefit to those taking glycaemic controlling drugs (Figure 4) as the mean eGFR at the end of the study was 6% higher in the intervention group (ROM 1.06, 1.02 to 1.10). Heterogeneity between studies was low (I^2=0%) yielding a prediction interval of 1.01 to 1.11. Sensitivity analyses showed that this result was robust (Table S5). Adding the CKD stage 1 to 4 studies, there was no significant difference between the intervention and comparator groups and the heterogeneity between studies was high (I^2=85%). We assessed the outlier[40-42] as having high attrition

For peer review only - http://bmjopen.bmj.com/site/about/guidelines.xhtml

bias as of the 1887 who were randomised, only 487 completed the 104 day study period. After excluding this study, there was a significant benefit to the intervention group, which was similar to that for the studies of CKD stage 3 and 4 patients.

A study[63 64] of patients with CKD stage 3 reported a 22% higher endpoint eGFR from taking sodium bicarbonate (ROM 1.22, 1.10 to 1.36). Another study[13] of patients with CKD stage 4, reported that taking sodium bicarbonate produced no significant effect on renal function in terms of creatinine clearance (ROM 0.94, 0.85 to 1.05). Pooling these data, there was no significant treatment effect, based on renal function (Table S5).

Figure 5 summarises the effects of treatment on renal function by drug class for the four drug classes.

Proteinuria

Data pooling for proteinuria was possible only for antihypertensive drugs in five studies. For studies of CKD stages 3 and 4 and overall, there was no significant treatment effect (Figure S5), with ROM 0.91 (0.78 to 1.05) and low heterogeneity at 41.3%. All sensitivity analyses produced the same result of no treatment effect with low heterogeneity (Table S5).

Two studies of lipid-modifying drugs reported proteinuria. The ATIC study[72] of patients with CKD stages 2 to 4 reported that treatment reduced urinary albumin excretion and provided medians and ranges of the AER (77, 3 to 2509 mg/24 hours for the intervention group, and 107, 5 to 3545 mg/24 hours for the comparator group). The PREVEND IT lipid study[66] of patients with CKD stages 1 to 3 reported that treatment slightly increased urinary albumin excretion by quoting medians and interquartile ranges (21.8 and 11.6 to 41.9 mg/24 hours for the intervention group, and 20.3 and 12.5 to 40.5 mg/24 hours for the control group).

For peer review only - http://bmjopen.bmj.com/site/about/guidelines.xhtml

One glycaemic control study reported proteinuria as an outcome. The CANVAS program[19 20] reported that treatment significantly reduced albuminuria compared with the placebo group. ACR was expressed on the geometric mean scale, as 13% lower in the intervention group compared with the controls (95% CI 1% to 24%) in patients with CKD stage 3a, and 26% lower (20% to 31%) in patients with CKD stages 3b and 4.

One sodium bicarbonate study[63 64] reported that treatment lowered the ACR by quoting median and interquartile range 257.1 (205.5 to 305.0 mg/g Cr) for the intervention group and 300.3 (241.8 to 347.5 mg/g Cr) for the control group.

Adverse events

There were no significant differences in the risk of adverse events in the studies of antihypertensives, lipid-modifying drugs and glycaemic control agents (Table S5). Neither of the bicarbonate studies reported adverse events.

Maintenance dialysis and kidney transplantation

Two studies of antihypertensive drugs reported the number of patients who deteriorated and required maintenance dialysis or kidney transplantation. The risk in the intervention group was 31% lower than in the control group in the HALT PKD study[51] of patients with stages 3 and 4 CKD (relative risk 0.69, 95% CI 0.50 to 0.96). In the RENAAL study[92-96], which was based on patients with CKD stages 2 to 4, treatment with antihypertensives led to a 23% reduction in risk of deterioration to kidney failure (RR 0.77, 0.64 to 0.93). There were no significant differences in this outcome in the studies of lipid-modifying drugs or glycaemic control drugs (Table S5).

For peer review only - http://bmjopen.bmj.com/site/about/guidelines.xhtml

For peer review only

Cardiovascular events

There were no significant differences between the intervention and control groups in the risk of cardiovascular events in the studies of antihypertensives whereas treatment with lipid-modifying drugs led to a reduction in risk of 36% (RR 0.64, 0.52 to 0.80, I^2= 30%) (Figure 6). All the sensitivity analyses yielded similar results and heterogeneity generally remained low (Table S5). The reduction in risk was slightly higher when data were added from the studies of patients that included those with CKD stages 1 and/or 2, but this reduction in risk was not significant (Table S5).

The TECOS glycaemic control study[88 89] reported the number of patients who experienced cardiovascular events in patients with CKD stage 3 patients. They reported no significant differences between the intervention and control groups (Table S5).

Mortality

In both our analyses of the studies of antihypertensive drugs and glycaemic control drugs we found no significant differences, between the intervention and comparator groups, in the risk of cardiovascular mortality and all-cause mortality (Table S5).

Two studies reported the effect of lipid-modifying drugs on the risk of cardiovascular mortality in patients with CKD stages 3 and 4. In the FIELD study[83-85], the risk was 47% lower in the intervention group (RR 0.53, 0.30 to 0.92), and in the AFCAPS/TEXCAPS study[57-59], there was no significant difference. The latter study was not based on an intention-to-treat analysis.

For peer review only - http://bmjopen.bmj.com/site/about/guidelines.xhtml

In the studies of patients with CKD stage 3 and 4 only, treatment with lipid-modifying drugs reduced the risk of all-cause mortality by 26% (RR 0.74, 0.56 to 0.98) (Figure 7, Table S5).

DISCUSSION

Main findings

We found limited data on the effects of antihypertensive, lipid-modifying, and glycaemic control drugs and sodium bicarbonate on the progression of renal function in patients with CKD stages 3 and 4. There was heterogeneity of patient populations in the included studies. Pooling data provided no evidence of benefit or impairment of renal function in these patients attributable to antihypertensive drugs. There was some suggestion of benefit to renal function from taking glycaemic control drugs and lipid-modifying drugs, and a clear reduction in cardiovascular disease and all-cause mortality by taking lipid-modifying drugs. There was insufficient evidence to draw any conclusions about sodium bicarbonates. There was only sufficient data to study the effects of antihypertensive drugs on proteinuria and we found no significant treatment effect.

Comparison with other reviews

Our review is comparable to two reviews of drug interventions in patients with CKD, as they report changes in eGFR as outcome measures. Our findings on the protective effect of lipid-modifying drugs is consistent with the findings of Sandhu et al,[9] who concluded that statins produce a small reduction in the progression of CKD in patients with cardiovascular disease. Our results are also consistent with those of Lewis et al[16] who reported the preliminary results of a review that considered the effect on kidney function of lowering low density lipoprotein cholesterol with statins. Their preliminary findings suggested that for patients taking statins there was a highly significant reduction in the annual rate of

For peer review only - http://bmjopen.bmj.com/site/about/guidelines.xhtml

CKD progression compared with control groups (based on the annual rate of change in eGFR). Both of these studies considered general populations with CKD. Our finding that lipid-modifying drugs reduce the risk of cardiovascular events and all-cause mortality also concurs with other reviews of patients with CKD.[108-110]

Strengths and limitations

We have identified, evaluated, and compared the effects of different drug interventions in reducing the rate of CKD progression in patients with CKD stages 3 and 4. By focusing on this patient group, we took a primary care perspective on the management of CKD. Our search was extensive and comprehensive, with no restrictions on language or publication type. However, the generalisability of our findings is limited by the paucity of trials, the high statistical heterogeneity and high clinical heterogeneity in terms of co-morbidities. Paucity of data restricted our ability to explore the baseline characteristics of studies and the effects of lipid-modifying drugs, glycaemic control drugs and bicarbonates. We were also restricted in our ability to investigate the link between treatment effect and drug dose and assess publication bias fully. We pooled data in cases where there were few studies or high statistical heterogeneity to give an overall indication of treatment effect. There was a wide variation of study characteristics (in terms of population size and follow up period) and baseline characteristics (in terms of male/female split, smoking status, ethnicity, and hypertensive status). The generalisability of the findings is also limited by the effect of drugs on CKD being secondary in all the studies. For example, antihypertensives target hypertension and glycaemic control drugs target diabetes. Our ability to assess the quality of the included studies was restricted, because for nearly half the studies, we could not assess allocation concealment bias, and for around a quarter of the studies we could not ascertain the risk of attrition bias or whether the participants, personnel and outcomes assessors had been blinded. Further bias may have arisen as most of the reports in our included studies were subgroup or post-hoc analyses of trials. The balance achieved by randomising

For peer review only - http://bmjopen.bmj.com/site/about/guidelines.xhtml

the whole trial population may not apply to subgroups of patients. However, we were unable to check this as few of these studies reported the necessary baseline characteristics, split by intervention group. Limitations may also arise from the possible impact on patient management decisions following the introduction of CKD staging in 2002. Before that, patients were treated on the basis of the perceived cause of CKD rather than the CKD itself. A number of the included studies had recruitment periods that began before 2002, so their populations would be a mixture of patients managed under the old and new guidance. However, in spite of all these limitations, the results of our analyses are consistent with those of other studies.

Implications for clinical practice

In populations with CKD stages 3 and 4, lipid-modifying drugs and glycaemic control drugs may improve renal function, and lipid-modifying drugs are associated with a reduction in cardiovascular events. Although antihypertensive drugs and sodium bicarbonate are presumed to reduce CKD progression,[8 11-13] we did not find a significant treatment effect. However, the paucity of data and high heterogeneity does not allow strong recommendations for clinical practice.

Our finding of limited data is important given that CKD is a common progressive disease. The drugs we considered are commonly used, but studies have concentrated on cardiovascular outcomes and not CKD outcomes.

CONCLUSIONS

This review suggests that glycaemic control and lipid regulation may improve renal function in patients with moderate CKD. There was no evidence of either benefit or harm from antihypertensive drugs.

For peer review only

However, the data were limited and heterogeneity was high for many of the analyses. Therefore, the results of pooling the data should be interpreted with caution.

ACKNOWLEDGEMENTS

We would like to thank all those who clarified our queries about their trials and/or provided additional data. Thanks also due to: Dr Boby Mihaylova who helped produce the protocol for this review; Dr Richard Stevens, who provided some statistical advice; Dr Brian Shine, who provided advice on pooling different measures of CKD and proteinuria; Professor Paul Glasziou, Dr Boby Mihaylova, and from the Patient & Public Involvement Group, Dr Elizabeth Holloway, Dr Peter Kirby, and Mrs Rachael Patel, for their comments about the manuscript and helpful suggestions that have improved the text; and, the Stakeholder Group and Steering Committee of our NIHR Programme Grant, for discussions that inspired this paper.

COMPETING INTERESTS

Kathryn Taylor, Julie McLellan, Jeffrey Aronson and Susannah Fleming receive funding from the NIHR Programme for Applied Research.

Jan Verbakel receives funding from the NIHR Community Healthcare MIC, Nuffield Department of Primary Care Health Sciences, University of Oxford NIHR Diagnostic Evidence Co-operative (DEC). Daniel Lasserson receives funding from the NIHR Community Healthcare MIC.

Rafael Perera receives funding from the NIHR Oxford Biomedical Research Centre Programme, the NIHR Programme for Applied Research, the NIHR HPRU Gastrointestinal Infections Group, and the NIHR Diagnostic Evidence Co-operative (DEC).

FUNDING

For peer review only

This article presents independent research funded by the NIHR under the Programme Grants for Applied Research programme (RP-PG-1210-12003). The views expressed are those of the authors and not necessarily those of the NHS, the NIHR or the Department of Health.

AUTHOR CONTRIBUTIONS

JM, RP, CB, JYV, JA, DSL, CoC, KST, BS, RH and AB were involved in the conception and design of the study. NR, JM and JA devised the search strategy. NR completed the search. JM, NP, KST, JYV and SF selected studies for inclusion, and KST, JM, JYV and NP extracted data. KST carried out the data analysis with advice from RP. KST and JM together wrote the first draft of the manuscript and all authors contributed critically to subsequent revisions and approved the final manuscript. KST and JM had full access to all data in the study and take responsibility for the integrity and accuracy of the data analysis. KST and JM are guarantors.

ETHICAL APPROVAL

Not required.

DATA SHARING

No additional data are available.

TRANSPARENCY

The lead author (the manuscript's guarantor) affirms that the manuscript is an honest, accurate, and transparent account of the study being reported; that no important aspects of the study have been omitted; and that any discrepancies from the study as planned (and, if relevant, registered) have been explained.

For peer review only

FIGURE LEGENDS

Figure 1. PRISMA diagram

Figure 2. Ratio of means of estimated glomerular filtration rate at the end of the trials for antihypertensives vs comparator (boxes) and pooled estimates across studies (diamonds) calculated by the random effects DerSimonian and Laird method, split by chronic kidney disease stage

CKD – Chronic kidney disease; CHARMa and CHARMb – CKD stage 3 and 4 respectively with estimated glomerular filtration rate measured by the MDRD equation; IDNTa - Intervention is irbesartan; IDNTb - Intervention is amlodipine.

Figure 3. Ratio of means of estimated glomerular filtration rate at the end of the trials for lipid-modifying drugs vs comparator (boxes) and pooled estimates across studies (diamonds) calculated by the random effects DerSimonian and Laird method, split by chronic kidney disease stage

CKD – Chronic kidney disease; ASCOT-LLAa - Atorvastatin and amlodipine vs placebo and amlodipine; ASCOT-LLAb - Atorvastatin and atenolol vs placebo and atenolol; GISSI-HFa – CKD stage 3; GISSI-HFb- CKD stage 4; GREACEa – Atorvastatin vs usual care for the group with metabolic syndrome; GREACEb – Atorvastatin vs usual care for the group without metabolic syndrome; SHARPa – CKD stages 1 to 3; SHARPb – CKD stage 4.

Figure 4. Ratio of means estimated glomerular filtration rate at the end of the trials for glycaemic control drugs vs comparator (boxes) and pooled estimates across studies (diamonds) calculated by the random effects DerSimonian and Laird method, split by chronic kidney disease stage

CKD – Chronic kidney disease; Bode 2015L – Intervention is low dose canagliflozin (100mg per day); EMPA-REG H – Intervention is high dose empagliflozin (25 mg daily); EMPA-REG H – Intervention is low dose empagliflozin (10 mg daily); Kohan 2014L – Intervention is low dose dapagliflozin (5 mg per day); CANVASa – CKD stage 3a, CANVASb – CKD stage 3b; SHARPa – CKD stages 1 to 3; SHARPb – CKD stage 4; TECOSa – CKD stage 3a; TECOSb – CKD stage 3b.

For peer review only - http://bmjopen.bmj.com/site/about/guidelines.xhtml

For peer review only

Figure 5. Effect of treatment on estimated glomerular filtration rate by drug class: chronic kidney disease stage 3 and 4 only

ROM>1 favoured the intervention group (higher values are better). "Studies" refers to the study of different populations. Of the 35 included studies, 15 reported renal function of patients with CKD stage 3 and/or stage 4. Of these, 9 reported renal function of a single population and 6 reported results for two CKD subgroups or dose dependent subgroups.

Figure 6. Relative risk of cardiovascular disease during trials of lipid-modifying drugs vs comparator (boxes) and pooled estimates across trials (diamonds) calculated by the random effects DerSimonian and Laird method, split by chronic kidney disease stage.

GREACEa – Atorvastatin vs usual care for the group with metabolic syndrome; GREACEb – Atorvastatin vs usual care for the group without metabolic syndrome.

Figure 7. Relative risk of all-cause mortality during trials of lipid-modifying drugs vs comparator (boxes) and pooled estimates across trials (diamonds) calculated by the random effects DerSimonian and Laird method, split by chronic kidney disease stage.

GREACEa – Atorvastatin vs usual care for the group with metabolic syndrome; GREACEb – Atorvastatin vs usual care for the group without metabolic syndrome.

References

1. Hill NR, Fatoba ST, Oke JL, et al. Global Prevalence of Chronic Kidney Disease - A Systematic Review and Meta-Analysis. *PloS one* 2016;11(7):e0158765. doi: 10.1371/journal.pone.0158765 [published Online First: 2016/07/08]

2. Go AS, Chertow GM, Fan D, et al. Chronic kidney disease and the risks of death, cardiovascular events, and hospitalization. *N Engl J Med* 2004;351(13):1296-305. doi: 10.1056/NEJMoa041031

For peer review only - http://bmjopen.bmj.com/site/about/guidelines.xhtml

3. National Institute for Health and Care Excellence. Chronic kidney disease: early identification and management of chronic kidney disease in adults in primary and secondary care (CG182). London: National Clinical Guideline Centre, 2014.

4. Levey AS, Eckardt KU, Tsukamoto Y, et al. Definition and classification of chronic kidney disease: a position statement from Kidney Disease: Improving Global Outcomes (KDIGO). *Kidney Int* 2005;67(6):2089-100. doi: 10.1111/j.1523-1755.2005.00365.x [published Online First: 2005/05/11]

5. Levin A, Stevens PE. Summary of KDIGO 2012 CKD Guideline: behind the scenes, need for guidance, and a framework for moving forward. *Kidney Int* 2014;85(1):49-61. doi: 10.1038/ki.2013.444 [published Online First: 2013/11/29]

6. de Lusignan S, Tomson C, Harris K, et al. UK Prevalence of Chronic Kidney Disease for the Adult Population Is 6.76% Based on Two Creatinine Readings. *Nephron Clinical Practice* 2012;120(2):c107-c07.

7. Oke J, Shine B, McFadden E, et al. Trends in serum creatinine testing in Oxfordshire, UK, 1993-2013: a population-based cohort study. *BMJ open* 2015;5(12):e009459. doi: 10.1136/bmjopen-2015-009459 [published Online First: 2015/12/18]

8. Peterson JC, Adler S, Burkart JM, et al. Blood pressure control, proteinuria, and the progression of renal disease. The Modification of Diet in Renal Disease Study. *Ann Intern Med* 1995;123(10):754-62.

9. Sandhu S, Wiebe N, Fried LF, et al. Statins for improving renal outcomes: a meta-analysis. *J Am Soc Nephrol* 2006;17(7):2006-16. doi: 10.1681/ASN.2006010012

10. Wang PH, Lau J, Chalmers TC. Meta-analysis of effects of intensive blood-glucose control on late complications of type I diabetes. *Lancet* 1993;341(8856):1306-9.

11. Phisitkul S, Khanna A, Simoni J, et al. Amelioration of metabolic acidosis in patients with low GFR reduced kidney endothelin production and kidney injury, and better preserved GFR. *Kidney Int* 2010;77(7):617-23. doi: 10.1038/ki.2009.519

For peer review only

For peer review only - http://bmjopen.bmj.com/site/about/guidelines.xhtml

12. Mahajan A, Simoni J, Sheather SJ, et al. Daily oral sodium bicarbonate preserves glomerular filtration rate by slowing its decline in early hypertensive nephropathy. *Kidney Int* 2010;78(3):303-9. doi: 10.1038/ki.2010.129

13. Brito-Ashurst Id, Varagunam M, Raftery MJ, et al. Bicarbonate supplementation slows progression of CKD and improves nutritional status. *Journal of the American Society of Nephrology* 2009;20(9):2075-84.

14. Lv J, Ehteshami P, Sarnak MJ, et al. Effects of intensive blood pressure lowering on the progression of chronic kidney disease: a systematic review and meta-analysis. *CMAJ* 2013;185(11):949-57. doi: 10.1503/cmaj.121468

15. Bakris GL. Slowing nephropathy progression: focus on proteinuria reduction. *Clin J Am Soc Nephrol* 2008;3 Suppl 1:S3-10. doi: 10.2215/CJN.03250807

16. Lewis A, Emberson JR, Blackwell LJ, et al. Effect of Lowering LDL-Cholesterol on Kidney Function: Meta-Analysis of Data from 120,000 Participants in 21 Randomized Trials. *Journal of the American Society of Nephrology Abstract supplement* 2012;23:685A.

17. Liberati A, Altman DG, Tetzlaff J, et al. The PRISMA statement for reporting systematic reviews and meta-analyses of studies that evaluate healthcare interventions: explanation and elaboration. *BMJ* 2009;339:b2700. doi: 10.1136/bmj.b2700

18. Levey AS, Bosch JP, Lewis JB, et al. A more accurate method to estimate glomerular filtration rate from serum creatinine: a new prediction equation. Modification of Diet in Renal Disease Study Group. *Ann Intern Med* 1999;130(6):461-70. [published Online First: 1999/03/13]

19. Neuen BL, Ohkuma T, Neal B, et al. Cardiovascular and Renal Outcomes With Canagliflozin According to Baseline Kidney Function: Data from the CANVAS Program. *Circulation* 2018 doi: 10.1161/circulationaha.118.035901 [published Online First: 2018/06/27]

20. Neal B, Perkovic V, Matthews DR. Canagliflozin and Cardiovascular and Renal Events in Type 2 Diabetes. *N Engl J Med* 2017;377(21):2099. doi: 10.1056/NEJMc1712572 [published Online First: 2017/11/23]

For peer review only

For peer review only - http://bmjopen.bmj.com/site/about/guidelines.xhtml

21. Higgins JPT, Green S. Cochrane Handbook for Systematic Reviews of Interventions: Wiley 2011.

22. Higgins JP. Commentary: Heterogeneity in meta-analysis should be expected and appropriately quantified. *International journal of epidemiology* 2008;37(5):1158-60. doi: 10.1093/ije/dyn204 [published Online First: 2008/10/04]

23. DerSimonian R, Laird N. Meta-analysis in clinical trials. *Controlled clinical trials* 1986;7(3):177-88. [published Online First: 1986/09/01]

24. Riley RD, Higgins JP, Deeks JJ. Interpretation of random effects meta-analyses. *Bmj* 2011;342:d549. doi: 10.1136/bmj.d549 [published Online First: 2011/02/12]

25. Higgins JP, Thompson SG, Spiegelhalter DJ. A re-evaluation of random-effects meta-analysis. *Journal of the Royal Statistical Society Series A, (Statistics in Society)* 2009;172(1):137-59. doi: 10.1111/j.1467-985X.2008.00552.x [published Online First: 2009/04/22]

26. Friedrich JO, Adhikari NK, Beyene J. The ratio of means method as an alternative to mean differences for analyzing continuous outcome variables in meta-analysis: a simulation study. *BMC Med Res Methodol* 2008;8:32. doi: 10.1186/1471-2288-8-32

27. Friedrich JO, Adhikari NK, Beyene J. Ratio of means for analyzing continuous outcomes in meta-analysis performed as well as mean difference methods. *J Clin Epidemiol* 2011;64(5):556-64. doi: 10.1016/j.jclinepi.2010.09.016 [published Online First: 2011/03/31]

28. Levey AS, Stevens LA, Schmid CH, et al. A new equation to estimate glomerular filtration rate. *Ann Intern Med* 2009;150(9):604-12. [published Online First: 2009/05/06]

29. Mantel N, Haenszel W. Statistical aspects of the analysis of data from retrospective studies of disease. *Journal of the National Cancer Institute* 1959;22(4):719-48. [published Online First: 1959/04/01]

30. Greenland S, Robins JM. Estimation of a common effect parameter from sparse follow-up data. *Biometrics* 1985;41(1):55-68. [published Online First: 1985/03/01]

For peer review only

For peer review only - http://bmjopen.bmj.com/site/about/guidelines.xhtml

31. Cornell JE, Mulrow CD, Localio R, et al. Random-effects meta-analysis of inconsistent effects: a time for change. *Ann Intern Med* 2014;160(4):267-70. doi: 10.7326/m13-2886 [published Online First: 2014/04/15]

32. Rover C, Knapp G, Friede T. Hartung-Knapp-Sidik-Jonkman approach and its modification for random-effects meta-analysis with few studies. *BMC Med Res Methodol* 2015;15:99. doi: 10.1186/s12874-015-0091-1 [published Online First: 2015/11/18]

33. Hartung J, Knapp G. A refined method for the meta-analysis of controlled clinical trials with binary outcome. *Statistics in medicine* 2001;20(24):3875-89. [published Online First: 2002/01/10]

34. Peters JL, Sutton AJ, Jones DR, et al. Contour-enhanced meta-analysis funnel plots help distinguish publication bias from other causes of asymmetry. *J Clin Epidemiol* 2008;61(10):991-6. doi: 10.1016/j.jclinepi.2007.11.010 [published Online First: 2008/06/10]

35. Stata Statistical Software: Release 14 [program]. College Station, TX: StataCorp LP, 2015.

36. Review Manager (RevMan) [program]. Version 5.3. Copenhagen: The Nordic Cochrane Centre, 2014.

37. Hawkins NM, Jhund PS, Pozzi A, et al. Severity of renal impairment in patients with heart failure and atrial fibrillation: implications for non-vitamin K antagonist oral anticoagulant dose adjustment. *European journal of heart failure* 2016;18(9):1162-71. doi: 10.1002/ejhf.614 [published Online First: 2016/09/07]

38. Hillege HL, Nitsch D, Pfeffer MA, et al. Renal function as a predictor of outcome in a broad spectrum of patients with heart failure. *Circulation* 2006;113(5):671-8. doi: 10.1161/circulationaha.105.580506 [published Online First: 2006/02/08]

39. Pfeffer MA, Swedberg K, Granger CB, et al. Effects of candesartan on mortality and morbidity in patients with chronic heart failure: the CHARM-Overall programme. *Lancet* 2003;362(9386):759-66. [published Online First: 2003/09/19]

For peer review only

For peer review only - http://bmjopen.bmj.com/site/about/guidelines.xhtml

40. Leiter LA, Cefalu WT, de Bruin TW, et al. Long-term maintenance of efficacy of dapagliflozin in patients with type 2 diabetes mellitus and cardiovascular disease. *Diabetes Obes Metab* 2016;18(8):766-74. doi: 10.1111/dom.12666 [published Online First: 2016/03/25]

41. Cefalu WT, Leiter LA, de Bruin TW, et al. Dapagliflozin's Effects on Glycemia and Cardiovascular Risk Factors in High-Risk Patients With Type 2 Diabetes: A 24-Week, Multicenter, Randomized, Double-Blind, Placebo-Controlled Study With a 28-Week Extension. *Diabetes care* 2015;38(7):1218-27. doi: 10.2337/dc14-0315 [published Online First: 2015/04/09]

42. Leiter LA, Cefalu WT, de Bruin TW, et al. Dapagliflozin added to usual care in individuals with type 2 diabetes mellitus with preexisting cardiovascular disease: a 24-week, multicenter, randomized, double-blind, placebo-controlled study with a 28-week extension. *J Am Geriatr Soc* 2014;62(7):1252-62. doi: 10.1111/jgs.12881 [published Online First: 2014/06/04]

43. Hou FF, Zhang X, Zhang GH, et al. Efficacy and safety of benazepril for advanced chronic renal insufficiency. *N Engl J Med* 2006;354(2):131-40. doi: 10.1056/NEJMoa053107 [published Online First: 2006/01/13]

44. Fassett RG, Robertson IK, Ball MJ, et al. Effect of atorvastatin on kidney function in chronic kidney disease: a randomised double-blind placebo-controlled trial. *Atherosclerosis* 2010;213(1):218-24.

45. Sakata Y, Shiba N, Takahashi J, et al. Clinical impacts of additive use of olmesartan in hypertensive patients with chronic heart failure: the supplemental benefit of an angiotensin receptor blocker in hypertensive patients with stable heart failure using olmesartan (SUPPORT) trial. *European heart journal* 2015; 36(15). http://cochranelibrary-wiley.com/o/cochrane/clcentral/articles/437/CN-01255437/frame.html

https://www.ncbi.nlm.nih.gov/pmc/articles/PMC4466154/pdf/ehu504.pdf.

46. Sakata Y, Nochioka K, Miura M, et al. Supplemental benefit of an angiotensin receptor blocker in hypertensive patients with stable heart failure using olmesartan (SUPPORT) trial--rationale

For peer review only - http://bmjopen.bmj.com/site/about/guidelines.xhtml

and design. *Journal of cardiology* 2013;62(1):31-6. doi: 10.1016/j.jjcc.2013.02.011 [published Online First: 2013/06/19]

47. Kimura, Genjiro, Kasahara, et al. Effects of atorvastatin on renal function in patients with dyslipidemia and chronic kidney disease: assessment of clinical usefulness in CKD patients with atorvastatin (ASUCA) trial. *Clinical & Experimental Nephrology* 2016;8:8.

48. Management of Elevated Cholesterol in the Primary Prevention Group of Adult Japanese (MEGA) Study Group. Design and baseline characteristics of a study of primary prevention of coronary events with pravastatin among Japanese with mildly elevated cholesterol levels. *Circulation journal : official journal of the Japanese Circulation Society* 2004;68(9):860-7. [published Online First: 2004/08/27]

49. Nakamura H, Mizuno K, Ohashi Y, et al. Pravastatin and cardiovascular risk in moderate chronic kidney disease. *Atherosclerosis* 2009;206(2):512-7.

50. Nakamura H, Arakawa K, Itakura H, et al. Primary prevention of cardiovascular disease with pravastatin in Japan (MEGA Study): a prospective randomised controlled trial. *Lancet* 2006;368(9542):1155-63. doi: 10.1016/s0140-6736(06)69472-5 [published Online First: 2006/10/03]

51. Torres VE, Abebe KZ, Chapman AB, et al. Angiotensin blockade in late autosomal dominant polycystic kidney disease. *N Engl J Med* 2014;371(24):2267-76. doi: 10.1056/NEJMoa1402686 [published Online First: 2014/11/18]

52. Lewis EJ, Hunsicker LG, Clarke WR, et al. Renoprotective effect of the angiotensin-receptor antagonist irbesartan in patients with nephropathy due to type 2 diabetes. *N Engl J Med* 2001;345(12):851-60. doi: 10.1056/NEJMoa011303 [published Online First: 2001/09/22]

53. Evans M, Bain SC, Hogan S, et al. Irbesartan delays progression of nephropathy as measured by estimated glomerular filtration rate: post hoc analysis of the Irbesartan Diabetic Nephropathy Trial. *Nephrology, dialysis, transplantation : official publication of the European Dialysis and*

For peer review only

For peer review only - http://bmjopen.bmj.com/site/about/guidelines.xhtml

Transplant Association - European Renal Association 2012;27(6):2255-63. doi: 10.1093/ndt/gfr696 [published Online First: 2011/12/17]

54. Berl T, Hunsicker LG, Lewis JB, et al. Cardiovascular outcomes in the Irbesartan Diabetic Nephropathy Trial of patients with type 2 diabetes and overt nephropathy. *Ann Intern Med* 2003;138(7):542-9. [published Online First: 2003/04/02]

55. Lambers Heerspink HJ, Weldegiorgis M, Inker LA, et al. Estimated GFR decline as a surrogate end point for kidney failure: a post hoc analysis from the Reduction of End Points in Non-Insulin-Dependent Diabetes With the Angiotensin II Antagonist Losartan (RENAAL) study and Irbesartan Diabetic Nephropathy Trial (IDNT). *American journal of kidney diseases : the official journal of the National Kidney Foundation* 2014;63(2):244-50. doi: 10.1053/j.ajkd.2013.09.016 [published Online First: 2013/11/12]

56. Pfeffer MA, Braunwald E, Moye LA, et al. Effect of captopril on mortality and morbidity in patients with left ventricular dysfunction after myocardial infarction. Results of the survival and ventricular enlargement trial. The SAVE Investigators. *N Engl J Med* 1992;327(10):669-77. doi: 10.1056/nejm199209033271001 [published Online First: 1992/09/13]

57. Downs JR, Clearfield M, Weis S, et al. Primary prevention of acute coronary events with lovastatin in men and women with average cholesterol levels: results of AFCAPS/TexCAPS. Air Force/Texas Coronary Atherosclerosis Prevention Study. *Jama* 1998;279(20):1615-22. [published Online First: 1998/06/05]

58. Kendrick J, Shlipak MG, Targher G, et al. Effect of lovastatin on primary prevention of cardiovascular events in mild CKD and kidney function loss: a post hoc analysis of the Air Force/Texas Coronary Atherosclerosis Prevention Study. *American Journal of Kidney Diseases* 2010;55(1):42-9.

59. Downs JR, Beere PA, Whitney E, et al. Design & rationale of the Air Force/Texas Coronary Atherosclerosis Prevention Study (AFCAPS/TexCAPS). *The American journal of cardiology* 1997;80(3):287-93. [published Online First: 1997/08/01]

For peer review only - http://bmjopen.bmj.com/site/about/guidelines.xhtml

60. Robins SJ, Collins D, Wittes JT, et al. Relation of gemfibrozil treatment and lipid levels with major coronary events: VA-HIT: a randomized controlled trial. *Jama* 2001;285(12):1585-91. [published Online First: 2001/04/13]

61. Tonelli M, Collins D, Robins S, et al. Effect of gemfibrozil on change in renal function in men with moderate chronic renal insufficiency and coronary disease. *American Journal of Kidney Diseases* 2004;44(5):832-9.

62. Rubins HB, Robins SJ, Iwane MK, et al. Rationale and design of the Department of Veterans Affairs High-Density Lipoprotein Cholesterol Intervention Trial (HIT) for secondary prevention of coronary artery disease in men with low high-density lipoprotein cholesterol and desirable low-density lipoprotein cholesterol. *The American journal of cardiology* 1993;71(1):45-52. [published Online First: 1993/01/01]

63. Goraya N, Munoz-Maldonado Y, Simoni J, et al. Treatment of metabolic acidosis in chronic kidney disease with fruits and vegetables but not sodium bicarbonate yields fewer adverse cardiovascular events after five-years follow up. *Circulation Conference: Resuscitation Science Symposium, ReSS* 2017;136(Supplement 1)

64. Goraya N, Simoni J, Jo CH, et al. Treatment of metabolic acidosis in patients with stage 3 chronic kidney disease with fruits and vegetables or oral bicarbonate reduces urine angiotensinogen and preserves glomerular filtration rate. *Kidney International* 2014;86(5):1031-8.

65. Bianchi S, Bigazzi R, Campese VM. Intensive versus conventional therapy to slow the progression of idiopathic glomerular diseases. *American journal of kidney diseases : the official journal of the National Kidney Foundation* 2010;55(4):671-81. doi: 10.1053/j.ajkd.2009.11.006 [published Online First: 2010/01/26]

66. Asselbergs FW, Diercks GF, Hillege HL, et al. Effects of fosinopril and pravastatin on cardiovascular events in subjects with microalbuminuria. *Circulation* 2004;110(18):2809-16. doi: 10.1161/01.cir.0000146378.65439.7a [published Online First: 2004/10/20]

For peer review only - http://bmjopen.bmj.com/site/about/guidelines.xhtml

67. Huskey J, Lindenfeld J, Cook T, et al. Effect of simvastatin on kidney function loss in patients with coronary heart disease: findings from the Scandinavian Simvastatin Survival Study (4S). *Atherosclerosis* 2009;205(1):202-6.

68. Pedersen TR, Wilhelmsen L, Faergeman O, et al. Follow-up study of patients randomized in the Scandinavian simvastatin survival study (4S) of cholesterol lowering. *The American journal of cardiology* 2000;86(3):257-62. [published Online First: 2000/08/03]

69. Design and baseline results of the Scandinavian Simvastatin Survival Study of patients with stable angina and/or previous myocardial infarction. *The American journal of cardiology* 1993;71(5):393-400. [published Online First: 1993/02/15]

70. Sever PS, Dahlof B, Poulter NR, et al. Prevention of coronary and stroke events with atorvastatin in hypertensive patients who have average or lower-than-average cholesterol concentrations, in the Anglo-Scandinavian Cardiac Outcomes Trial--Lipid Lowering Arm (ASCOT-LLA): a multicentre randomised controlled trial. *Lancet* 2003;361(9364):1149-58. doi: 10.1016/s0140-6736(03)12948-0 [published Online First: 2003/04/11]

71. Gupta A, Chang CL, Collier D, et al. The relationship between statin therapy and progression of renal damage among 10305 hypertensive patients randomised in the ascot-Lipid-Lowering Arm (LLA). *Atherosclerosis Supplements* 2011;Conference: 79th European Atherosclerosis Society Congress, EAS 2011 Gothenburg Sweden. Conference Start: 20110626 Conference End: 20110629. Conference Publication:(var.pagings). 12 (1):158-59.

72. Nanayakkara PWB, Guldener Cv, Wee PMt, et al. Effect of a treatment strategy consisting of pravastatin, vitamin E, and homocysteine lowering on carotid intima-media thickness, endothelial function, and renal function in patients with mild to moderate chronic kidney disease: results from the Anti-Oxidant Therapy in Chronic Renal Insufficiency (ATIC) Study. *Archives of Internal Medicine* 2007;167(12):1262-70.

73. Tavazzi L, Maggioni AP, Marchioli R, et al. Effect of rosuvastatin in patients with chronic heart failure (the GISSI-HF trial): a randomised, double-blind, placebo-controlled trial. *Lancet*

For peer review only - http://bmjopen.bmj.com/site/about/guidelines.xhtml

2008;372(9645):1231-9. doi: 10.1016/s0140-6736(08)61240-4 [published Online First: 2008/09/02]

74. Tavazzi L, Tognoni G, Franzosi MG, et al. Rationale and design of the GISSI heart failure trial: a large trial to assess the effects of n-3 polyunsaturated fatty acids and rosuvastatin in symptomatic congestive heart failure. *European journal of heart failure* 2004;6(5):635-41. doi: 10.1016/j.ejheart.2004.03.001 [published Online First: 2004/08/11]

75. Athyros VG, Mikhailidis DP, Liberopoulos EN, et al. Effect of statin treatment on renal function and serum uric acid levels and their relation to vascular events in patients with coronary heart disease and metabolic syndrome. *Nephrology Dialysis Transplantation* 2007;22(1):118-27.

76. Athyros VG, Papageorgiou AA, Mercouris BR, et al. Treatment with atorvastatin to the National Cholesterol Educational Program goal versus 'usual' care in secondary coronary heart disease prevention. The GREek Atorvastatin and Coronary-heart-disease Evaluation (GREACE) study. *Current medical research and opinion* 2002;18(4):220-8. [published Online First: 2002/08/31]

77. Ford I, Bezlyak V, Stott DJ, et al. Reduced glomerular filtration rate and its association with clinical outcome in older patients at risk of vascular events: secondary analysis. *PLoS medicine* 2009;6 (1)(e16)

78. Patel A, MacMahon S, Chalmers J, et al. Effects of a fixed combination of perindopril and indapamide on macrovascular and microvascular outcomes in patients with type 2 diabetes mellitus (the ADVANCE trial): a randomised controlled trial. *Lancet* 2007;370(9590):829-40. doi: 10.1016/s0140-6736(07)61303-8 [published Online First: 2007/09/04]

79. Heerspink HJ, Ninomiya T, Perkovic V, et al. Effects of a fixed combination of perindopril and indapamide in patients with type 2 diabetes and chronic kidney disease. *European heart journal* 2010;31(23):2888-96. doi: 10.1093/eurheartj/ehq139 [published Online First: 2010/05/27]

For peer review only

For peer review only - http://bmjopen.bmj.com/site/about/guidelines.xhtml

80. Peters R, Beckett N, Poulter R, et al. Kidney function in the very elderly with hypertension: data from the hypertension in the very elderly (HYVET) trial. *Age and ageing* 2013;42(2):253-8. doi: 10.1093/ageing/afs109 [published Online First: 2012/08/23]

81. Beckett NS, Peters R, Fletcher AE, et al. Treatment of hypertension in patients 80 years of age or older. *N Engl J Med* 2008;358(18):1887-98. doi: 10.1056/NEJMoa0801369 [published Online First: 2008/04/02]

82. Bulpitt C, Fletcher A, Beckett N, et al. Hypertension in the Very Elderly Trial (HYVET): protocol for the main trial. *Drugs & aging* 2001;18(3):151-64. [published Online First: 2001/04/17]

83. Ting RD, Keech AC, Drury PL, et al. Benefits and safety of long-term fenofibrate therapy in people with type 2 diabetes and renal impairment: the FIELD Study. *Diabetes care* 2012;35(2):218-25.

84. The need for a large-scale trial of fibrate therapy in diabetes: the rationale and design of the Fenofibrate Intervention and Event Lowering in Diabetes (FIELD) study. [ISRCTN64783481]. *Cardiovascular diabetology* 2004;3:9. doi: 10.1186/1475-2840-3-9 [published Online First: 2004/12/02]

85. Keech A, Simes RJ, Barter P, et al. Effects of long-term fenofibrate therapy on cardiovascular events in 9795 people with type 2 diabetes mellitus (the FIELD study): randomised controlled trial. *Lancet* 2005;366(9500):1849-61. doi: 10.1016/s0140-6736(05)67667-2 [published Online First: 2005/11/29]

86. Zinman B, Wanner C, Lachin JM, et al. Empagliflozin, Cardiovascular Outcomes, and Mortality in Type 2 Diabetes. *N Engl J Med* 2015;373(22):2117-28. doi: 10.1056/NEJMoa1504720 [published Online First: 2015/09/18]

87. Wanner C, Inzucchi SE, Lachin JM, et al. Empagliflozin and Progression of Kidney Disease in Type 2 Diabetes. *N Engl J Med* 2016;375(4):323-34. doi: 10.1056/NEJMoa1515920 [published Online First: 2016/06/15]

For peer review only

For peer review only - http://bmjopen.bmj.com/site/about/guidelines.xhtml

88. Cornel JH, Bakris GL, Stevens SR, et al. Effect of Sitagliptin on Kidney Function and Respective Cardiovascular Outcomes in Type 2 Diabetes: Outcomes From TECOS. *Diabetes care* 2016;39(12):2304-10. doi: 10.2337/dc16-1415 [published Online First: 2016/10/16]

89. Green JB, Bethel MA, Armstrong PW, et al. Effect of Sitagliptin on Cardiovascular Outcomes in Type 2 Diabetes. *N Engl J Med* 2015;373(3):232-42. doi: 10.1056/NEJMoa1501352 [published Online First: 2015/06/09]

90. Zannad F, McMurray JJ, Krum H, et al. Eplerenone in patients with systolic heart failure and mild symptoms. *N Engl J Med* 2011;364(1):11-21. doi: 10.1056/NEJMoa1009492 [published Online First: 2010/11/16]

91. Rossignol P, Dobre D, McMurray JJ, et al. Incidence, determinants, and prognostic significance of hyperkalemia and worsening renal function in patients with heart failure receiving the mineralocorticoid receptor antagonist eplerenone or placebo in addition to optimal medical therapy: results from the Eplerenone in Mild Patients Hospitalization and Survival Study in Heart Failure (EMPHASIS-HF). *Circulation Heart failure* 2014;7(1):51-8. doi: 10.1161/circheartfailure.113.000792 [published Online First: 2013/12/04]

92. Brenner BM, Cooper ME, de Zeeuw D, et al. Effects of losartan on renal and cardiovascular outcomes in patients with type 2 diabetes and nephropathy. *N Engl J Med* 2001;345(12):861-9. doi: 10.1056/NEJMoa011161 [published Online First: 2001/09/22]

93. de Zeeuw D, Remuzzi G, Parving HH, et al. Proteinuria, a target for renoprotection in patients with type 2 diabetic nephropathy: lessons from RENAAL. *Kidney Int* 2004;65(6):2309-20. doi: 10.1111/j.1523-1755.2004.00653.x [published Online First: 2004/05/20]

94. Kowey PR, Dickson TZ, Zhang Z, et al. Losartan and end-organ protection--lessons from the RENAAL study. *Clinical cardiology* 2005;28(3):136-42. [published Online First: 2005/04/09]

95. Winkelmayer WC, Zhang Z, Shahinfar S, et al. Efficacy and safety of angiotensin II receptor blockade in elderly patients with diabetes. *Diabetes care* 2006;29(10):2210-7. doi: 10.2337/dc06-0570 [published Online First: 2006/09/28]

For peer review only - http://bmjopen.bmj.com/site/about/guidelines.xhtml

96. Brenner BM, Cooper ME, de Zeeuw D, et al. The losartan renal protection study--rationale, study design and baseline characteristics of RENAAL (Reduction of Endpoints in NIDDM with the Angiotensin II Antagonist Losartan). *Journal of the renin-angiotensin-aldosterone system : JRAAS* 2000;1(4):328-35. doi: 10.3317/jraas.2000.062 [published Online First: 2002/04/23]

97. Pitt B, Pfeffer MA, Assmann SF, et al. Spironolactone for heart failure with preserved ejection fraction. *N Engl J Med* 2014;370(15):1383-92. doi: 10.1056/NEJMoa1313731 [published Online First: 2014/04/11]

98. Crouse JR, 3rd, Raichlen JS, Riley WA, et al. Effect of rosuvastatin on progression of carotid intima-media thickness in low-risk individuals with subclinical atherosclerosis: the METEOR Trial. *Jama* 2007;297(12):1344-53. doi: 10.1001/jama.297.12.1344 [published Online First: 2007/03/27]

99. Shepherd J, Blauw GJ, Murphy MB, et al. The design of a prospective study of Pravastatin in the Elderly at Risk (PROSPER). PROSPER Study Group. PROspective Study of Pravastatin in the Elderly at Risk. *The American journal of cardiology* 1999;84(10):1192-7. [published Online First: 1999/11/24]

100. Shepherd J, Blauw GJ, Murphy MB, et al. Pravastatin in elderly individuals at risk of vascular disease (PROSPER): a randomised controlled trial. *Lancet* 2002;360(9346):1623-30. [published Online First: 2002/11/30]

101. Baigent C, Landray MJ, Reith C, et al. The effects of lowering LDL cholesterol with simvastatin plus ezetimibe in patients with chronic kidney disease (Study of Heart and Renal Protection): a randomised placebo-controlled trial. *Lancet* 2011;377(9784):2181-92.

102. Haynes R, Lewis D, Emberson J, et al. Effects of lowering LDL cholesterol on progression of kidney disease. *Journal of the American Society of Nephrology* 2014;25(8):1825-33.

103. Sharp Collaborative G. Study of Heart and Renal Protection (SHARP): randomized trial to assess the effects of lowering low-density lipoprotein cholesterol among 9,438 patients with chronic

For peer review only - http://bmjopen.bmj.com/site/about/guidelines.xhtml

kidney disease. *American heart journal* 2010;160(5):785-94.e10. doi: 10.1016/j.ahj.2010.08.012 [published Online First: 2010/11/26]

104. Bode B, Stenlof K, Harris S, et al. Long-term efficacy and safety of canagliflozin over 104 weeks in patients aged 55-80 years with type 2 diabetes. *Diabetes Obes Metab* 2015;17(3):294-303. doi: 10.1111/dom.12428 [published Online First: 2014/12/17]

105. Bode B, Stenlof K, Sullivan D, et al. Efficacy and safety of canagliflozin treatment in older subjects with type 2 diabetes mellitus: a randomized trial. *Hospital practice (1995)* 2013;41(2):72-84. doi: 10.3810/hp.2013.04.1020 [published Online First: 2013/05/18]

106. Kohan DE, Fioretto P, Tang W, et al. Long-term study of patients with type 2 diabetes and moderate renal impairment shows that dapagliflozin reduces weight and blood pressure but does not improve glycemic control. *Kidney Int* 2014;85(4):962-71. doi: 10.1038/ki.2013.356 [published Online First: 2013/09/27]

107. de Brito-Ashurst I, Varagunam M, Raftery MJ, et al. Bicarbonate supplementation slows progression of CKD and improves nutritional status. *J Am Soc Nephrol* 2009;20(9):2075-84. doi: 10.1681/ASN.2008111205

108. Palmer SC, Navaneethan SD, Craig JC, et al. HMG CoA reductase inhibitors (statins) for people with chronic kidney disease not requiring dialysis. *The Cochrane database of systematic reviews* 2014(5):Cd007784. doi: 10.1002/14651858.CD007784.pub2 [published Online First: 2014/06/01]

109. Zhang X, Xiang C, Zhou YH, et al. Effect of statins on cardiovascular events in patients with mild to moderate chronic kidney disease: a systematic review and meta-analysis of randomized clinical trials. *BMC cardiovascular disorders* 2014;14:19. doi: 10.1186/1471-2261-14-19 [published Online First: 2014/02/18]

110. Herrington WG, Emberson J, Mihaylova B, et al. Impact of renal function on the effects of LDL cholesterol lowering with statin-based regimens: a meta-analysis of individual participant data

For peer review only

For peer review only - http://bmjopen.bmj.com/site/about/guidelines.xhtml

from 28 randomised trials. *The lancet Diabetes & endocrinology* 2016;4(10):829-39. doi: 10.1016/s2213-8587(16)30156-5 [published Online First: 2016/08/02]

For peer review only

For peer review only - http://bmjopen.bmj.com/site/about/guidelines.xhtml

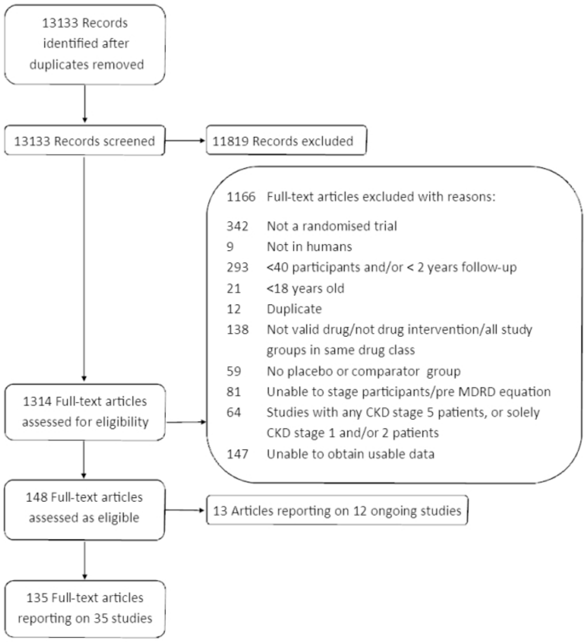

PRISMA diagram

104x148mm (300 x 300 DPI)

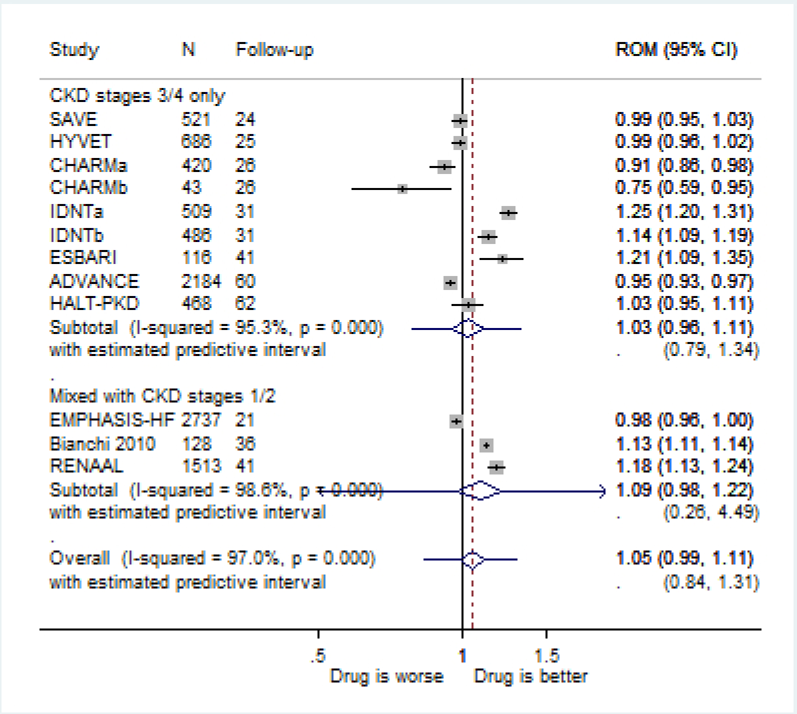

Ratio of means of estimated glomerular filtration rate at the end of the trials for antihypertensives vs comparator (boxes) and pooled estimates across studies (diamonds) calculated by the random effects DerSimonian and Laird method, split by chronic kidney disease stage
CKD – Chronic kidney disease; CHARMa and CHARMb – CKD stage 3 and 4 respectively with estimated glomerular filtration rate measured by the MDRD equation; IDNTa - Intervention is irbesartan; IDNTb - Intervention is amlodipine.

140x125mm (72 x 72 DPI)

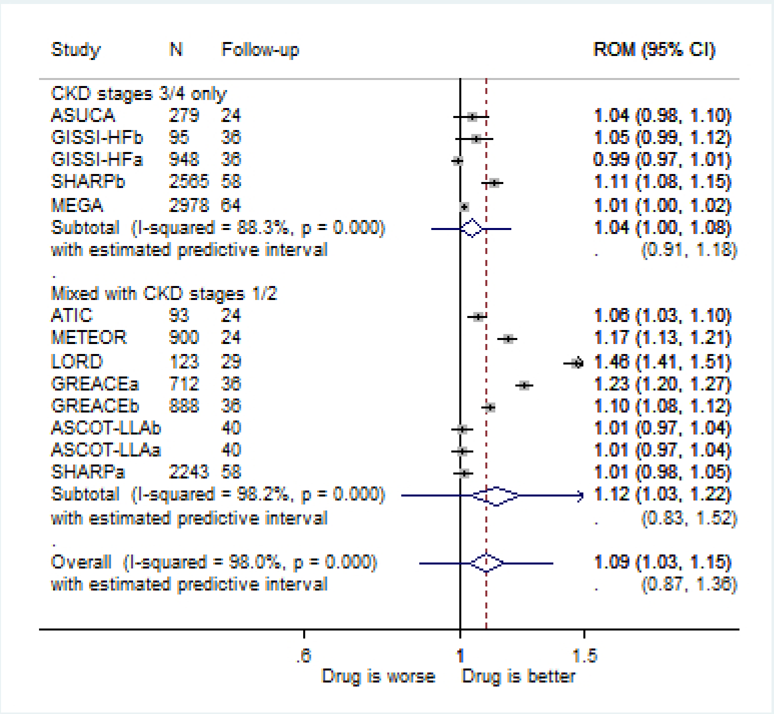

Ratio of means of estimated glomerular filtration rate at the end of the trials for lipid-modifying drugs vs comparator (boxes) and pooled estimates across studies (diamonds) calculated by the random effects DerSimonian and Laird method, split by chronic kidney disease stage
CKD – Chronic kidney disease; ASCOT-LLAa - Atorvastatin and amlodipine vs placebo and amlodipine; ASCOT-LLAb - Atorvastatin and atenolol vs placebo and atenolol; GISSI-HFa – CKD stage 3; GISSI-HFb- CKD stage 4; GREACEa – Atorvastatin vs usual care for the group with metabolic syndrome; GREACEb – Atorvastatin vs usual care for the group without metabolic syndrome; SHARPa – CKD stages 1 to 3; SHARPb – CKD stage 4.

136x125mm (72 x 72 DPI)

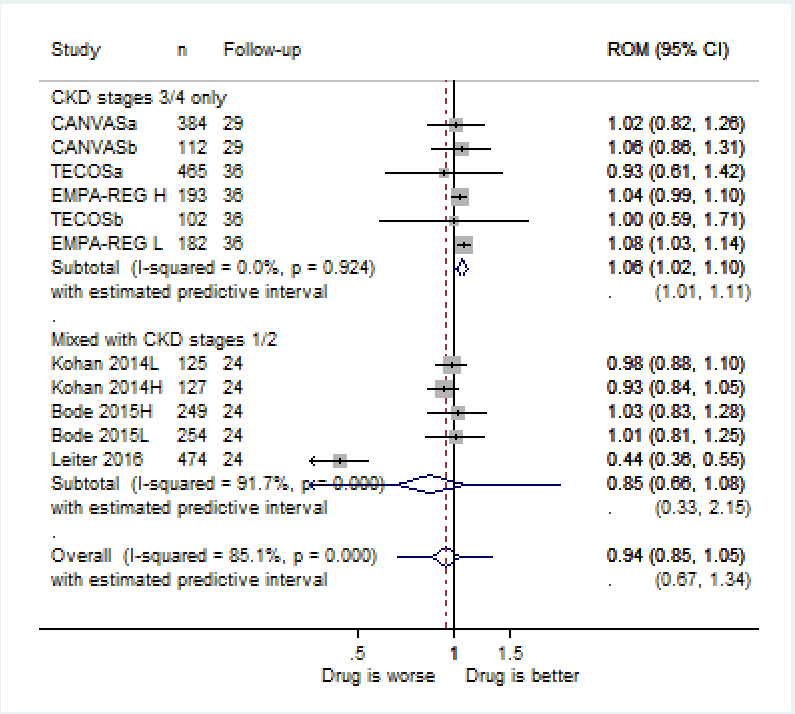

Ratio of means estimated glomerular filtration rate at the end of the trials for glycaemic control drugs vs comparator (boxes) and pooled estimates across studies (diamonds) calculated by the random effects DerSimonian and Laird method, split by chronic kidney disease stage
CKD – Chronic kidney disease; Bode 2015L – Intervention is low dose canagliflozin (100mg per day); EMPA-REG H – Intervention is high dose empagliflozin (25 mg daily); EMPA-REG H – Intervention is low dose empagliflozin (10 mg daily); Kohan 2014L – Intervention is low dose dapagliflozin (5 mg per day); CANVASa – CKD stage 3a, CANVASb – CKD stage 3b; SHARPa – CKD stages 1 to 3; SHARPb – CKD stage 4; TECOSa – CKD stage 3a; TECOSb – CKD stage 3b.

140x125mm (72 x 72 DPI)

Drug class	Studies	Population	I^2	Ratio of means (95% confidence interval)
Anti-hypertensive	9	5433	95.3%	1.03 (0.96, 1.11)
Lipid modification	5	6865	88.3%	1.04 (1.00, 1.08)
Glycaemic control	6	1438	0%	1.06 (1.02, 1.10)
Bicarbonates	2	180	91.1%	1.08 (0.84. 1.38)

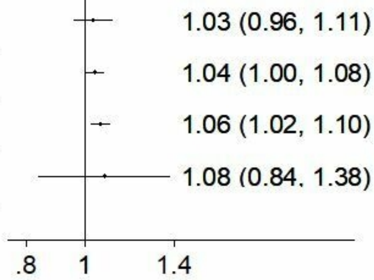

Effect of treatment on estimated glomerular filtration rate by drug class: chronic kidney disease stage 3 and 4 only
ROM>1 favoured the intervention group (higher values are better). “Studies” refers to the study of different populations. Of the 35 included studies, 15 reported renal function of patients with CKD stage 3 and/or stage 4. Of these, 9 reported renal function of a single population and 6 reported results for two CKD subgroups or dose dependent subgroups.

69x24mm (300 x 300 DPI)

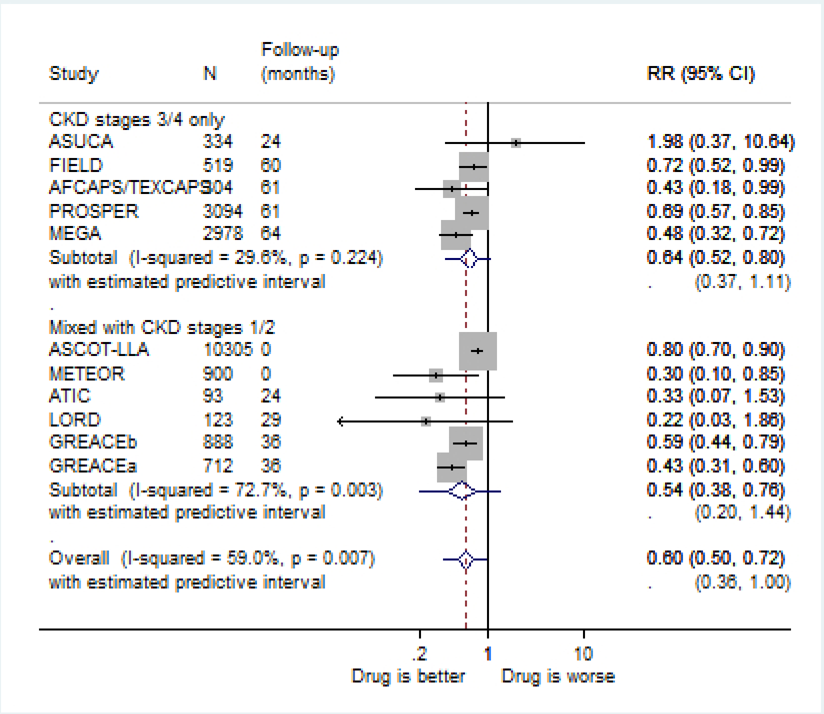

Relative risk of cardiovascular disease during trials of lipid-modifying drugs vs comparator (boxes) and pooled estimates across trials (diamonds) calculated by the random effects DerSimonian and Laird method, split by chronic kidney disease stage.
GREACEa – Atorvastatin vs usual care for the group with metabolic syndrome; GREACEb – Atorvastatin vs usual care for the group without metabolic syndrome.

145x125mm (72 x 72 DPI)

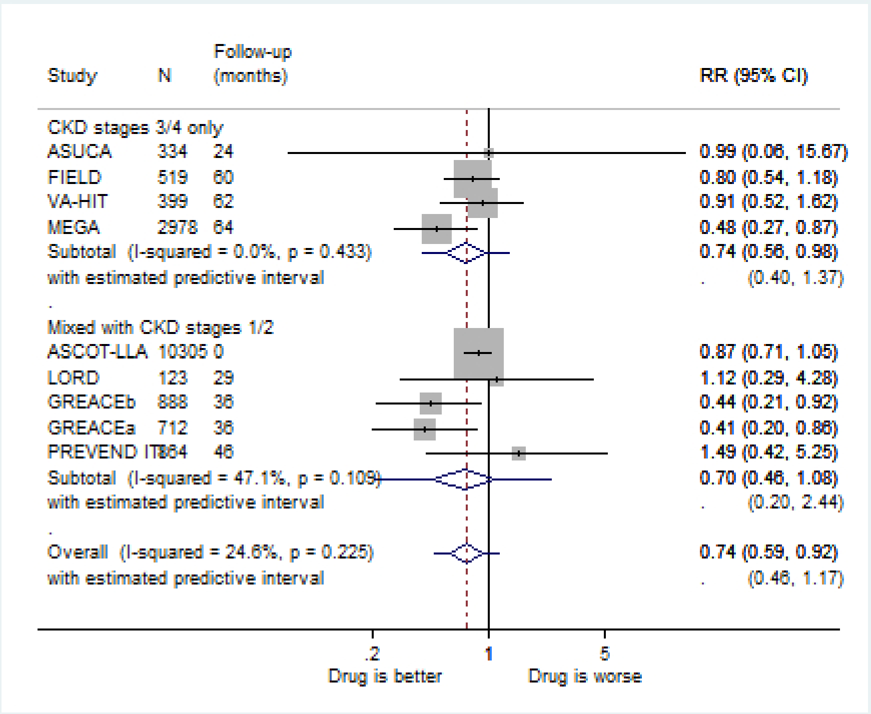

Relative risk of all-cause mortality during trials of lipid-modifying drugs vs comparator (boxes) and pooled estimates across trials (diamonds) calculated by the random effects DerSimonian and Laird method, split by chronic kidney disease stage.
GREACEa – Atorvastatin vs usual care for the group with metabolic syndrome; GREACEb – Atorvastatin vs usual care for the group without metabolic syndrome.

153x125mm (72 x 72 DPI)

Supplementary Figure 1. Kidney Disease Improving Global outcomes (KDIGO) 2012

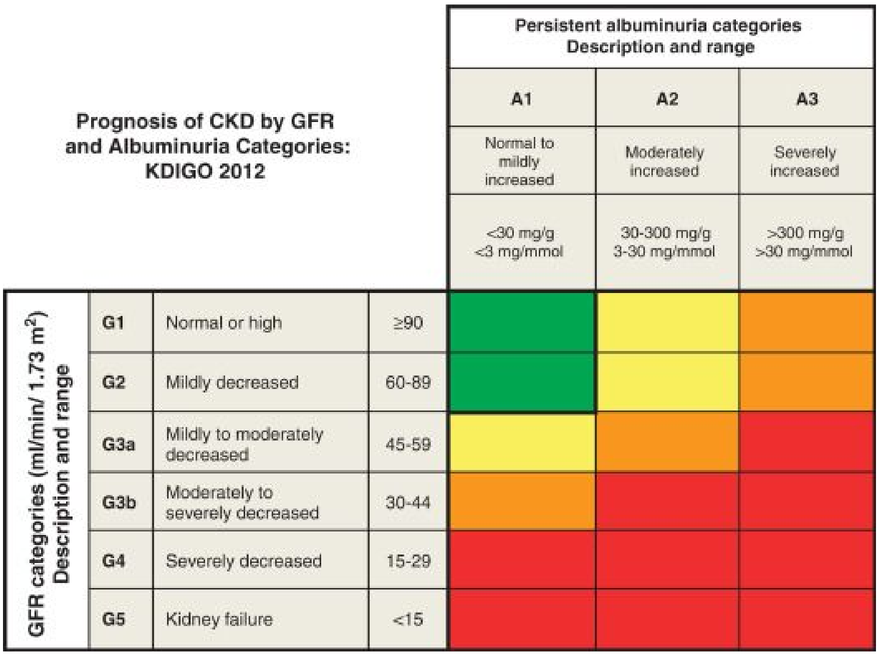

Albuminuria = proteinuria. Green: low risk (if no other markers of disease, no CKD); yellow moderately increased risk; orange: high risk; red: very high risk

review only

Supplementary Figure 2. Cochrane Collaboration’s Risk of bias

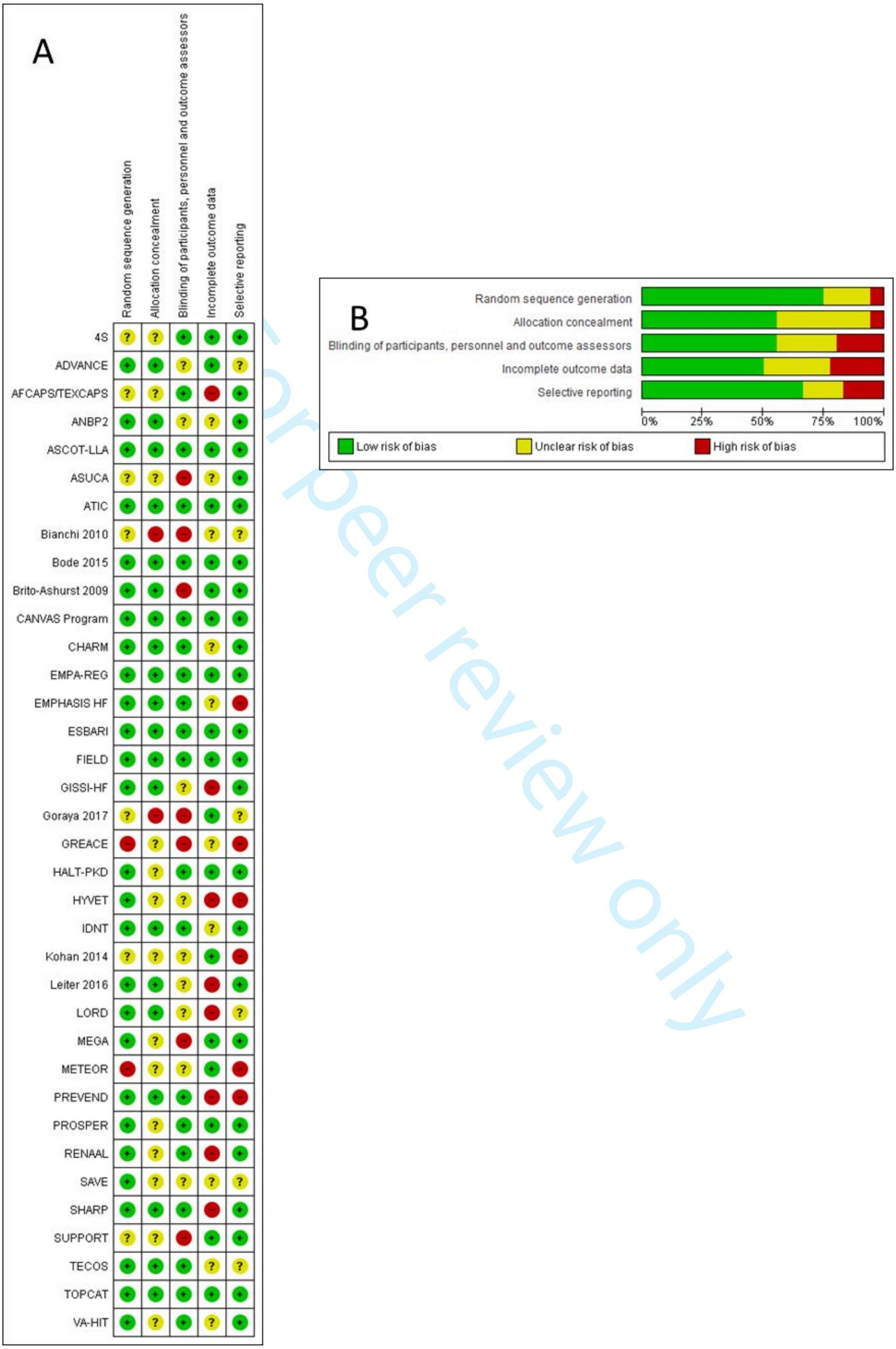

Based on 35 studies. A - Summary of review authors' judgements about each included study; B - Graph of review authors' judgements about each risk of bias item presented as percentages across all included studies

Supplementary Figure 3. Funnel plots and contour enhanced funnel plots, for estimated glomerular filtration rate data, split by drug group

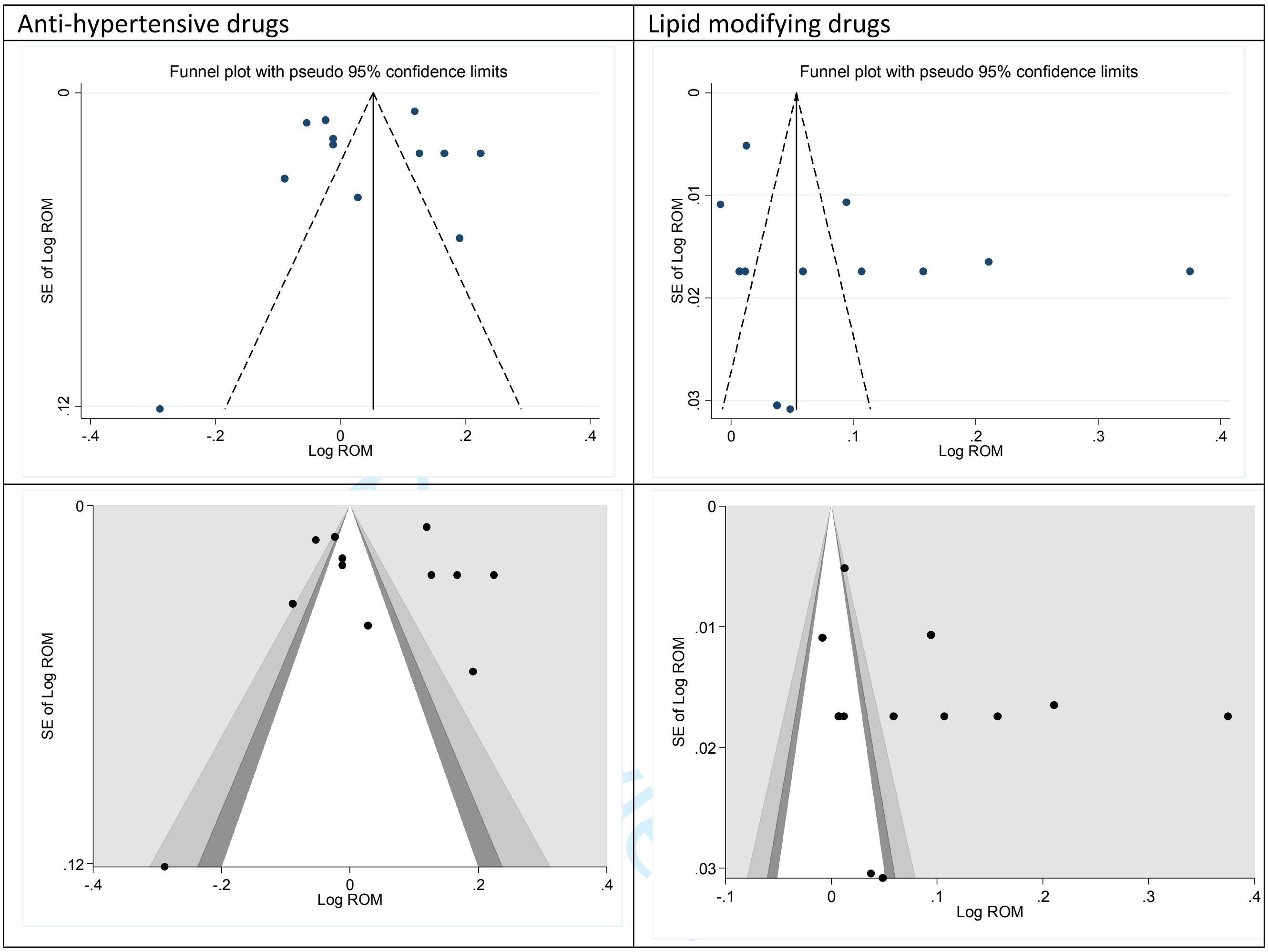

A positive Log ROM favours the intervention drug. Log refers to the natural logarithm.

Contour plots shading: light grey (p<1%); medium grey (1%<p<5%); dark grey (5%<p<10%); white (p>10%)

Supplementary Figure 4. Ratio of means of estimated glomerular filtration rate at the end of the trials for anti-hypertensives vs comparator (boxes) and pooled estimates across studies (diamonds) calculated by the random effects DerSimonian and Laird method, for chronic kidney disease stages 3 and 4 and split by drug class

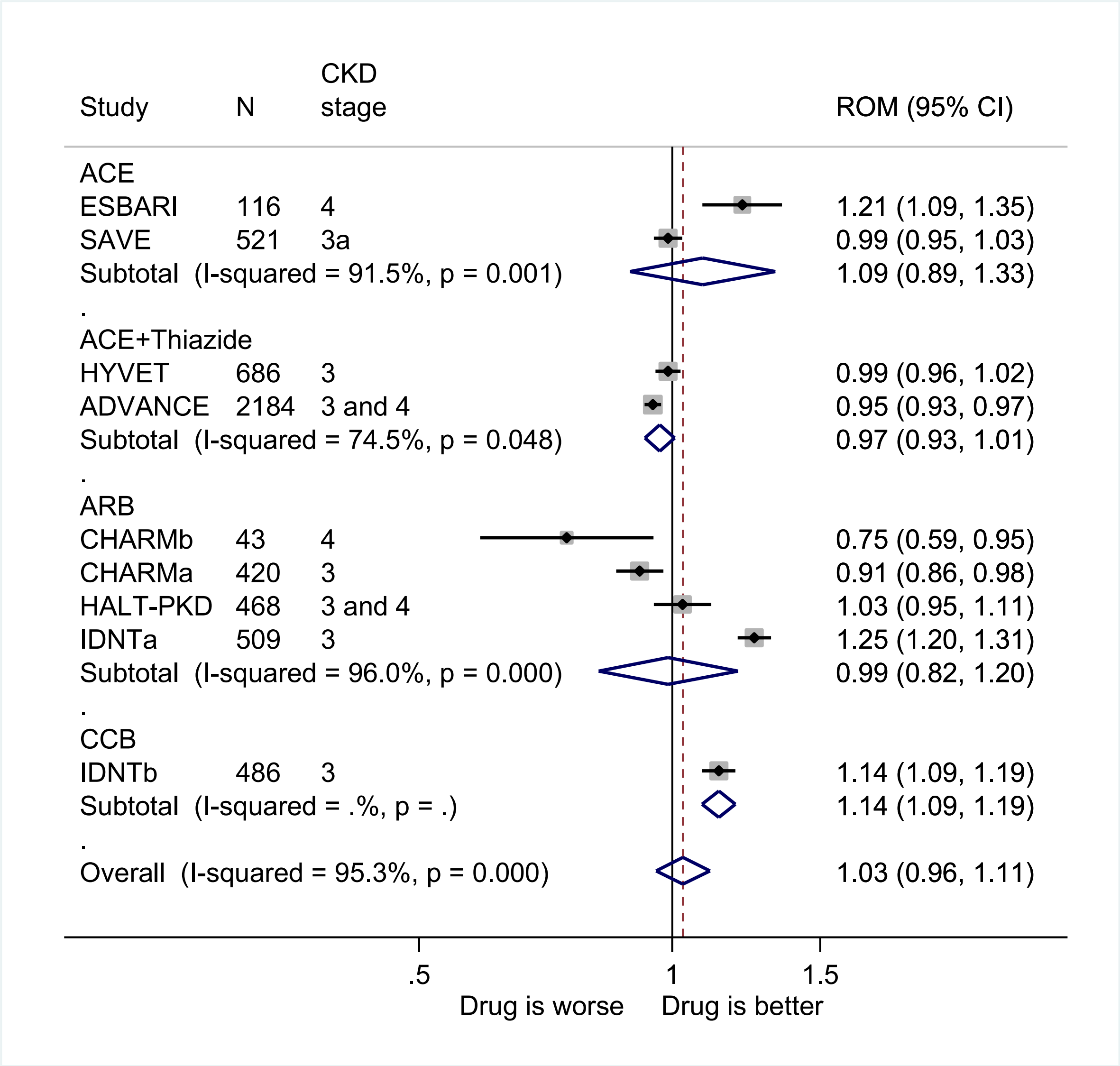

CKD – chronic kidney disease; CHARMa and CHARMb – CKD stage 3 and 4 respectively with eGFR measured by MDRD equation; IDNTa - intervention is Irbesartan; IDNTb - intervention is Amlodipine.

Supplementary Figure 5. Ratio of means of proteinuria at the end of the trials for anti-hypertensives vs comparator (boxes) and pooled estimates across studies (diamonds) calculated by the random effects DerSimonian and Laird method, split by chronic kidney disease stage

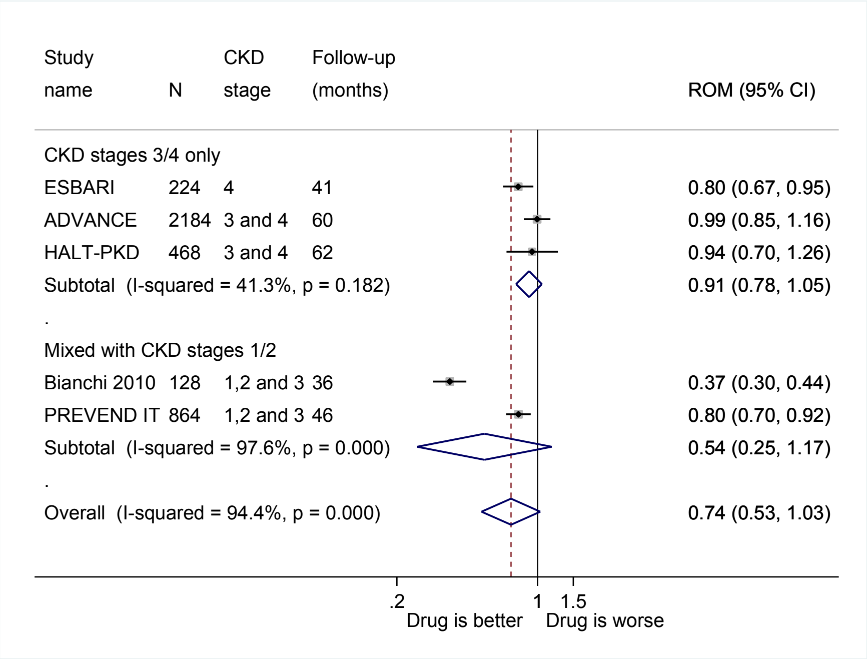

CKD – chronic kidney disease. Prediction interval for overall (0.20 to 2.70).

Supplementary Table 1. Kidney Disease Outcomes Quality Initiative classification (Levy 2003)

Stages of CKD	eGFR (ml/min/1.73m^2)	Description
1	≥ 90	Normal or increased GFR, with other evidence of kidney damage
2	60–89	Slight decrease in GFR, with other evidence of kidney damage
3A	45–59	Moderate decrease in GFR, with or without other evidence of kidney damage
3B	30–44	
4	15–29	Severe decrease in GFR, with or without other evidence of kidney damage
5	< 15	Established renal failure

CKD – Chronic kidney disease; GFR –glomerular filtration rate.

For peer review only

Supplementary Table 2. Characteristics of studies

Study (trial name/author)	Drug class	Trial N*	Analysis N*	Follow up (mths)**	CKD stages†	Population that we studied [method of obtaining data on subgroup for analysis]	Country
ADVANCE[79 80] §	A	10640	2184	60	3 and 4	Type II diabetes and, most likely, CKD stages 1 to 5. [Authors gave data for stages 3 and 4]	Multinational
Bianchi 2010[66]	A	128	128	36	1 to 3	Idiopathic chronic glomerulonephritis and CKD stages 1 to 3	Italy
CHARM[38-40] (3 trials) §	A	7599	463/845	26	3 and 4	Symptomatic chronic heart failure and, most likely, CKD stages 1 to 5. [Authors gave data for stages 3 and 4]	Multinational
EMPHASIS-HF[91 92]	A	2737	2737	21	1 to 3	Systolic heart failure and CKD stages 1, 2 and 3a.	Multinational
ESBARI[44] §	A	224	116	41	4	Advanced chronic renal insufficiency without diabetes and CKD stages 4 and 5. [Authors gave data for stage 4]	China
HALT-PKD[52] §	A	486	468	24	3 and 4	Moderately advanced autosomal dominant polycystic kidney disease and CKD stages 3 and 4. [Authors gave further data]	USA
HYVET[81-83] §	A	3845	686	25	3	Very elderly hypertensive population and, most likely, CKD stages 1 to 5. [Authors gave data for stage 3]	Multinational
IDNT[53-56]	A	1715	995	36	3	Hypertensive and with diabetic nephropathy due to type II diabetes and CKD stages 1 to 5 [Stage 3 data was published]	USA
PREVEND IT[67]	A/L‡	864	864	46	1 to 3	Microalbuminuria and CKD stages 1 to 3.	Netherlands
RENAAL[93-97]	A	1513	1513	41	2 to 4	Type II diabetic nephropathy and CKD stages 2 to 4.	Multinational
SAVE[57] §	A	2231	521	36	3a	Left ventricular dysfunction after M. CKD stages 1 to 5. [Authors gave data for stage 3a]	USA/Canada
SUPPORT[46 47]	A	1147	1147	53	1 to 4	Hypertensive with heart failure. CKD stages 1 to 4.	Japan
TOPCAT[98]	A	3445	3445	40	1 to 3	Symptomatic heart failure and preserved ejection fraction.	Multinational
4S[68-70]	L	4444	409	66	3	Coronary heart disease and elevated LDL and, most likely, CKD stages 1 to 5. [Stage 3 data was published]	Europe
AFCAPS/ TEXCAPS[58-60]	L	6605	304	61	2 to 4	With CKD without significant diabetes or cardiovascular disease [Stage 3 and 4 data was published]	USA
ASCOT-LLA[71 72] §	L‡	10305	10305	40	1 to 3	Hypertension and, most likely, CKD stages 1 to 5. [Stage 1 to 3 data was published and authors gave further data]	Europe
ASUCA[48] §	L	349	279/334	24	3 and 4	Dyslipidemia. [Stage 3 and 4 data was published and authors gave further data]	Japan

For peer review only

ATIC[73]	L	93	93	24	2 to 4	Mild/moderate CKD.	Netherlands
FIELD[84-86]	L	9795	519	60	3	Type II diabetes and renal impairment. CKD stages 1 to 3 [Stage 3 data was published]	Multinational
GISSI-HF[74 75] §	L	4631	1043	36	3 and 4	Chronic heart failure. [Obtained data on stages 3 and 4 from the company that owns the data]	Italy
GREACE[76 77]	L	1600	1600	36	1 to 3	Coronary heart disease, and CKD stages 1 to 3. [Authors reported a post-hoc analysis on those with and without metabolic syndrome]	Greece
LORD[45]	L	123	123	29	2 to 4	CKD stages 2 to 4.	Tasmania
MEGA[49-51]	L	7832	2978	64	3	Hypercholesterolemia and normal kidney function. CKD stages 1 to 3. [Stage 3 data was published]	Japan
METEOR[99]	L	984	984	24	1 to 3	Asymptomatic individuals with moderately elevated cholesterol and low risk of cardiovascular disease.	Multinational
PROSPER[78 100 101]	L	5796	3094	24	3 and 4	With pre-existing risk of vascular disease or risk of developing this condition. Most likely with CKD stages 1 to 5. [Stage 3 and 4 data was published]	Europe
SHARP[102-104]	L	9270	4808/4720	58	1 to 4	CKD stages 1 to 5. [Stage 1 to 4 data was published]	Multinational
VA-HIT[61-63]	L	399	399	24	3	Coronary disease and low HDL. Most likely CKD stages 1 to 5. [Stage 3 data was published]	USA
Bode 2015[105 106]	G	714	503	24	1 to 3	Type II diabetes and CKD.	Multinational
CANVAS Program[19 20] (2 trials) §	G	10142	496¶	29	3	Two trials of populations with type II diabetes and high cardiovascular risk with CKD stages 1 to 4. [Stage 3a and 3b data was published]	Multinational
EMPA-REG OUTCOME[87 88]	G	7020††	1819/7020	36	3 §§	Type II diabetes and CKD stages 1 to 3. [Stage 3 data was published]	Multinational
Kohan 2014[107]	G	252	252	24	2 to 4	Type II diabetes and moderate renal impairment with CKD stages 2 to 4.	Multinational
Leiter 2016[41-43] (2 trials)	G	1887	474	24	1 to 3	Two trials of populations with type II diabetes and cardiovascular disease with CKD stages 1 to 3. One of the trials also included participants with hypertension.	Multinational
TECOS[89 90]	G	14671	567/3321	36	3	Type II diabetes and cardiovascular disease and CKD stages 1 to 3. [Stage 3 data was published]	Multinational

For peer review only

Brito-Ashurst 2009[13]	B	134	134	24	4	Stage 4 chronic CKD (creatinine clearance 15 to 30 ml/min per 1.73 m^2 and metabolic acidosis (serum bicarbonate 16 to 20 mmol/l)	UK
Goraya 2017[64 65]	B	108	108	60	3	Stage 3 CKD and metabolic acidosis (plasma TCO_2 22 to 24 mmol/l)	USA

* Trial population size (single or combined trials) and analysis population (if two numbers are given, the first refers to the primary outcome); ** mean or median; † CKD stages are given for the analysis population; †† 7028 patients were randomised; ‡ 2X2 factorial trial; §Unpublished data was provided by the authors; §§ - Used data for CKD stage 3 in the main analysis and data for CKD stages 1 to 3 as a sensitivity analysis; ¶- Included eGFR data from time before longest follow-up. CKD – chronic kidney disease. Drug class: A - antihypertensive; L – lipid-modifying; G – glycaemic control; B- sodium bicarbonate.

For peer review only

Supplementary Table 3. Interventions of studies

Study	Drug class	Interventions (dose and frequency, if stated)
ADVANCE[79 80]	A	Perindopril (2 to 4 mg daily) + indapamide (0.625 to 1.25 mg daily) vs placebo
Bianchi 2010[66]	A	Ramipril (initially 10 mg daily) + irbesartan (300 mg daily) + atorvastatin (20 mg daily) + spironolactone (25 mg daily) vs ramipril (10 mg daily) + atorvastatin (10 mg daily), possibly + hydrochlorothiazide or furosemide, calcium antagonists, beta-blockers or alpha-receptor antagonists.
CHARM[38-40]	A	Candesartan (4 or 8 mg to 32 mg daily) vs placebo
EMPHASIS-HF[91 92]	A	Eplerenone (25 to 50 mg daily) vs placebo
ESBARI[44]	A	Benazepril (20 mg twice a day) vs placebo
HALT-PKD[52]	A	Telmisartan (40 to 80 mg daily) vs placebo
HYVET[81-83]	A	Indapamide initially (1.5 to 2 or 4 mg daily) and possibly + perindopril later to achieve target blood pressure vs placebo
IDNT[53-56]	A	Irbesartan (75 to 100 mg daily) vs amlodipine (2.5 to 10 mg daily) vs placebo
PREVEND IT[67]	A/L	2x2 factorial design: fosinopril (20 mg daily) or matching placebo and pravastatin (40 mg daily) or matching placebo<br>Data for antihypertensive analysis: (fosinopril+pravastatin or fosinopril+placebo) vs (placebo only or placebo + pravastatin)<br>Data for lipid-modifying analysis: (fosinopril+pravastatin or pravastatin+placebo) vs (placebo only or placebo + fosinopril)
RENAAL[93-97]	A	Losartan (5 to 100 mg daily) vs placebo
SAVE[57]	A	Captopril (6.5 to 50 mg 3 times a day) vs placebo
SUPPORT[46 47]	A	Olmesartan (5-10mg and increased to 40mg daily) vs placebo
TOPCAT[98]	A	Spironolactone (15-45 mg daily) vs placebo
4S[68-70]	L	Simvastatin (20 to 40 mg daily) vs placebo
AFCAPS/TEXCAPS[58-60]	L	Lovastatin (20 mg daily) vs placebo
ASCOT-LLA[71 72]	L	2x2 factorial design: atorvastatin (10 mg daily) + amlodipine (5 to 10 mg daily) vs atorvastatin (10 mg daily) + atenolol (50 to 100mg daily) vs placebo + amlodipine (5 to 10 mg daily) vs placebo + atenolol (50 to 100 mg daily)<br>Data for atenolol analysis: (atorvastatin + atenolol) vs (placebo + atenolol)<br>Data for amlodipine analysis: (atorvastatin + amlodipine) vs (placebo + amlodipine)
ASUCA[48]	L	Atorvastatin (10 to 5-20 mg daily) vs diet
ATIC[73]	L	Pravastatin (40 mg daily) + vitamin E (300 mg daily) + folic acid (15 mg daily) + pyridoxine hydrochloride (100 mg daily) + cyanacobalamin (1 mg daily) vs Placebo
FIELD[84-86]	L	Fenofibrate (200 mg daily) vs placebo

For peer review only

GISSI-HF[74 75]	L	Rosuvastatin (10mg) daily vs placebo
GREACE[76 77]	L	Atorvastatin (10 to 80 mg daily) vs usual care
LORD[45]	L	Atorvastatin (10 mg daily) vs placebo
MEGA[49-51]	L	Pravastatin (10 mg initially) + diet vs diet
METEOR[99]	L	Rosuvastatin (40 mg daily) vs placebo
PROSPER[78 100 101]	L	Pravastatin (40 mg daily) vs placebo
SHARP[102-104]	L	Simvastatin (20 mg daily) + ezetimibe (10 mg daily) vs placebo
VA-HIT[61-63]	L	Gemfibrozil (1200 mg daily) vs placebo
Bode 2015[105 106]	G	Canagliflozin (100 mg daily) vs canagliflozin (300 mg daily) vs placebo
CANVAS Program[19 20]	G	Canagliflozin (100mg-300mg) vs placebo
EMPA-REG OUTCOME[87 88]	G	Empagliflozin (25mg daily) vs empagliflozin (10mg daily) vs placebo
Kohan 2014[107]	G	Dapagliflozin (10 mg daily) vs dapagliflozin (50 mg daily) vs placebo
Leiter 2016[41-43]*	G	Dapagliflozin (10 mg daily) vs placebo
TECOS[89 90]	G	Sitagliptin (100 mg daily) vs placebo
Brito-Ashurst 2009[13]	B	Oral sodium bicarbonate (600 mg 3 times a day) vs routine standard care
Goraya 2017[64 65]	B	Oral sodium bicarbonate (> 22 mq/L) vs routine care

* Reported in Leiter 2014 and Cefalu 2015. Drug class: A – antihypertensive; L – lipid-modifying; G – glycaemic control; B – sodium bicarbonate

For peer review only

Table S3. Baseline characteristics

Trial	Trial N	Baseline N	Analysis N	CKD stages*	Drug class	Age Mean (SD)	Male (%)	Non-white ethnicity (%)	Current Smoker (%)	Body mass index Mean (SD)	Systolic blood pressure Mean (SD)	Diastolic blood pressure Mean (SD)
Trials for which baseline data was provided for all patients included in the analysis												
ADVANCE[79 80]	10640	2184	2184/ 2033	3 to 4	A	68.2 (6.4)	43.1	NS	10.4	28.7 (5.2)	147.0 (23.3)	79.8 (11.3)
Bianchi 2010[66]	128	128	128	1 to 3	A	53.2 (0.9)	64.1	NS	28.9	25.1 (0.3)	156.1 (1.3)	93.7 (0.7)
EMPHASIS-HF[91 92]	2737	2737	2737	1 to 3	A	68.6 (7.6)	77.5	17.1	NS	27.5 (4.9)	124.0 (17.0)	75.0 (10.0)
PREVEND IT[67] (A)	864	864	864	1 to 3	A	51.3 (11.8)	64.9	3.9	39.9	26.0 (4.5)	130.0 (17.5)	76.0 (10.0)
PREVEND IT[67] (L)	864	864	864	1 to 3	L	51.3 (11.8)	65.0	3.9	38.2	26.0 (4.0)	130.5 (17.5)	76.5 (10.0)
RENAAL[93-97]	1513	1513	1513	2 to 4	A	60.0 (7.0)	63.2	51.3	18.3	29.5 (6.0)	152.5 (19.5)	82.0 (10.5)
TOPCAT[98]	3445	3445	3445	1 to 3	A	68.7 (11.2)	48.5	11.1	10.5	31 (6.7)	130 (14.5)	80.0 (7.4)
4S[68-70]	4444	409	409	3	L	62.2 (5.0)	54.5	NS	16.1	25.9 (3.4)	143.1 (21.4)	83.7 (9.6)
AFCAPS/ TEXCAPS[58-60]	6605	304	304	2 to 4	L	62.0 (7.5)	78.3	1.0	7.9	26.5 (3.0)	141.6 (18.0)	79.0 (10.0)
ATIC[73]	93	93	93	2 to 4	L	53.0 (12.0)	56.9	NS	35.5	26.5 (4.5)	135.0 (20.9)	78.5 (12.0)
FIELD[84-86]	9795	519	519	3	L	66.5 (5.9)	41.2	NS	6.9	NS	NS	NS
GREACE[76 77]	1600	1600	1600	1 to 3	L	58.5 (7.5)	79.4	NS	NS	25.8 (4.4)	129.0 (16.9)	77.5 (7.9)
LORD[45]	123	123	123	2 to 4	L	60.2 (15.1)	65.0	0	NS	28.5 (6.0)	143.4 (19.8)	80.7 (12.6)
MEGA[49-51]	7832	2978	2978	3	L	60.0	24.3	NS	12.5	NS	132.9	NS
PROSPER[78 100 101]	5796	3094	3094	3	L	75.5 (3.4)	42.0	NS	22.9	27.0 (4.3)	154.7 (21.6)	83.7 (11.3)
VA-HIT[61-63]	2531	399	399	3	L	67.3 (5.2)	100	9.5	14.0	NS	134.0 (18.3)	77.2 (9.8)
Kohan 2014[107]	252	252	252	2 to 4	G	67.0 (8.1)	65.1	16.3	NS	NS	132.1 (16.4)	73.3 (9.3)
EMPA-REG OUTCOME[87 88]	7020†	1819	1819	3	G	67.1 (7.8)	67.8	NS	NS	31.0 (5.5)	136.2 (18.2)	74.5 (10.0)
Brito-Ashurst 2009[13]	134	134	134	4	B	54.8 (2.4)	51.5	48.0	NS	NS	123.9 (1.3)	75.8 (1.7)
Goraya 2017[64 65]	108	108	108	3	B	53.7 (5.1)	44.0	NS	NS	NS	162.3 (11.1)	NS

For peer review only

Trials for which baseline data was not provided for all patients included in the analysis but for a different subgroup of the trial population or the whole trial population(s)												
CHARM[38-40] (3 trials)	7599	2680	463/845	3 to 4	A	65.3 (11.6)	66.6	NS	14.2	NS	128.2 (18.7)	73.6 (10.7)
ESBARI[44]	224	224	116/224	4	A	44.7 (15.5)	49.5	NS	NS	22.8 (5.0)	152.4 (23.1)	85.9 (10.4)
HYVET[81-83]	3845	1194	686	3	A	83.8 (3.3)	31.0	NS	NS	25.0 (3.8)	173.7 (8.9)	NS
HALT-PKD[52]	486	486	468	3 to 4	A	48.7 (8.3)	48.3	6.5	NS	28.0 (5.2)	121.5 (15.6)	75.9 (10.9)
IDNT[53-56]	1715	1715	995	3	A	58.9 (7.8)	66.3	27.5	NS	30.8 (5.8)	159.0 (19.7)	87.0 (11.0)
SAVE[57]	2231	2231	521	3a	A	59.4	82.5	NS	NS	NS	112.5	70.0
SUPPORT[46 47]	1147	1147	1139	1 to 4	A	65.7 (10.2)	74.7	NS	NS	24.4 (4.1)	127.9 (18.1)	74.4 (12.0)
ASCOT-LLA[71 72]	10305	10305	10305	1 to 3	L	63.1 (8.5)	81.2	NS	32.7	23.6 (6.9)	164.2 (17.8)	95.0 (10.3)
ASUCA[48]	349	334	279/334	3 to 4	L	63.2 (8.1)	63.8	NS	14.1	25.6 (3.6)	133.2 (16.3)	76.8 (10.6)
GISSI-HF[74 75]	4631	4574	1043	3 to 4	L	68.0 (11.0)	77.4	NS	14.0	27.1 (4.5)	127.0 (18.0)	77.0 (10.0)
METEOR[99]	984	984	900	1 to 3	L	57.0 (6.0)	59.7	15.7	4.0	27.2 (4.0)	139.0 (21.0)	80.0 (12.0)
SHARP[102-104]	9270	6245	4808/ 4720	1 to 4	L	63.0 (12.0)	62.5	28.5	12.0	27.4 (5.5)	139.0 (21.0)	80.0 (12.0)
Bode 2015[105 106]	714	714	503	1 to 3	G	63.6 (6.2)	55.5	23.1	NS	31.6 (4.6)	NS	NS
CANVAS Program[19 20] (2 trials)	10142	10142	496	3	G	63.3 (8.3)	64.2	21.7	17.8	32.0 (5.9)	136.6 (15.8)	77.7 (9.7)
Leiter 2016[41-43] (2 trials)	1887	568	474/568	1 to 3	G	62.9 (7.3)	93.9	NS	NS	32.7 (5.5)	133.8 (13.5)	77.6 (8.5)
TECOS[89 90]	14671	3321	567/3321	3	G	68.3 (7.8)	63.6	68.7	7.4	30.5 (5.8)	136.0 (18.0)	76.7 (10.7)

NS – not stated; * CKD stages are given for the analysis population; Drug class: A – anti-hypertensive; L – lipid modifying; G – glycaemic control; B- sodium bicarbonate.

Supplementary Table 5. Results of analyses

	Effect measure (95% CI)	No of studies or populations*	I^2
**1. Estimated Glomerular Filtration Rate**			
Antihypertensive drugs			
Random effects, DerSimonian and Laird method (Base Case)	1.03 (0.96 to 1.11)	9	95.3%
Fixed effects, inverse-variance method	1.01 (0.99 to 1.02)	9	95.3%
Hartung-Knapp-Sidik-Jonkman method	1.03 (0.93 to 1.14)	9	95.3%
Excluding studies with estimates	0.98 (0.94 to 1.03)	7	81.0%
Excluding studies with high risk of attrition or allocation bias	1.03 (0.95 to 1.13)	8	95.8%
Using CKD EPI data for CHARM trial	1.03 (0.96 to 1.11)	9	95.1%
More conservative imputation, using a larger SE	1.03 (0.97 to 1.10)	9	92.5%
Adding studies with CKD stage 1/2 patients	1.05 (0.99 to 1.11)	12	97.0%
Hartung-Knapp-Sidik-Jonkman method and studies with CKD stage 1/2 patients	1.05 (0.97 to 1.13)	12	97.0%
Lipid modifying drugs			
Random effects, DerSimonian and Laird method (Base Case)	1.04 (1.0001 to 1.08)	5	88.3%
Fixed effects, inverse-variance method	1.02 (1.01 to 1.03)	5	88.3%
Hartung-Knapp-Sidik-Jonkman method	1.04 (0.98 to 1.10)	5	88.3%
Excluding studies with estimates (SHARPb)	1.01 (0.99 to 1.03)	4	45.3%
Excluding studies with high risk of attrition or allocation bias	1.01 (1.003 to 1.02)	2	0%
More conservative imputation, using a larger SE	1.03 (0.999 to 1.06)	5	73.9%
Adding studies with CKD stage 1/2 patients	1.09 (1.03 to 1.15)	13	98.0%
Hartung-Knapp-Sidik-Jonkman method and studies with CKD stage 1/2 patients	1.09 (1.02 to 1.16)	13	98.0%
Glycaemic control drugs			
Random effects, DerSimonian and Laird method (Base Case)	1.06 (1.02 to 1.10)	6	0%
Fixed effects, inverse-variance method	No change		
Hartung-Knapp-Sidik-Jonkman method	1.06 (1.01 to 1.11)	6	0%
Excluding studies with estimates	0.96 (0.69 to 1.34)	2	0%
Excluding studies with high risk of attrition or allocation bias	No change		
More conservative imputation, using a larger SE	No change		

For peer review only

Adding studies with CKD stage 1/2 patients	0.94 (0.85 to 1.05)	11	85.1%
Hartung-Knapp-Sidik-Jonkman method and studies with CKD stage 1/2 patients	0.94 (0.80 to 1.12)	11	85.1%
CKD stage 1 to 3 data for EMPA-REG and adding studies with stage 1/2 patients	0.93 (0.82 to 1.04)	11	81.4%
Excluding Leiter 2016 (outlier) and Adding studies with CKD stage 1/2 patients	1.04 (1.01 to 1.07)	10	0%
Sodium bicarbonate			
Random effects, DerSimonian and Laird method	1.08 (0.84 to 1.38)	2	91.1%
**2. Proteinuria**			
Antihypertensive drugs			
Random effects, DerSimonian and Laird method (Base Case)	0.91 (0.82 to 1.01)	3	41.3%
Fixed effects to inverse-variance method	No change		
Excluding studies with estimates	0.80 (0.67 to 0.95)	1	-
Excluding studies of PER and PCR	0.98 (0.86 to 1.12)	2	0%
Excluding studies with high risk of attrition or allocation bias	No change		
More conservative imputation to using a larger SE	No change		
Adding studies with CKD stage 1/2 patients	0.74 (0.53 to 1.03)	5	94.5%
**3. Adverse events**			
Anti-hypertensive drugs			
HALT-PKD, CKD stages 3-4	0.89 (0.74 to 1.07)	1	-
Adding studies with CKD stage 1/2 patients	0.94 (0.83 to 1.05)	4	58.0%
Lipid modifying drugs			
Random effects, DerSimonian and Laird method (Base Case)	0.91 (0.78 to 1.06)	3	17.8%
Fixed effects to Mantel–Haenszel method	0.92 (0.86 to 0.98)	3	17.8%
Excluding studies with high risk of attrition or allocation bias	0.96 (0.52 to 1.79)	2	55.8%
Adding studies with CKD stage 1/2 patients	1.02 (0.79 to 1.32	5	55.3%
Glycaemic control drugs			
EMPA-REG	0.96 (0.94 to 0.99)	1	-
Adding studies with CKD stage 1/2 patients	1.01 (0.96 to 1.06)	6	62.7%
**4. Combined maintenance dialysis and kidney transplantation**			
Anti-hypertensive drugs			

For peer review only

HALT-PKD	0.69 (0.50 to 0.96)	1	-
Adding RENAAL (CKD stages 2 to 4)	0.75 (0.64 to 0.88)	2	0%
Lipid modifying drugs			
Random effects, DerSimonian and Laird method (Base Case)	0.97 (0.88 to 1.08)	3	0%
Fixed effects to Mantel–Haenszel method	No change		
Excluding studies with high risk of attrition or allocation bias	1.20 (0.34 to 4.25)	2	0%
Adding studies with stages 1-4 CKD	0.97 (0.88 to 1.07)	5	7.8%
Glycaemic control drugs			
EMPA-REG	0.78 (0.61 to 1.01)	1	-
Adding studies with CKD stage 1/2 patients	0.64 (0.27 to 1.53)	4	38.2%
**5. Cardiovascular events**			
Antihypertensive drugs			
Random effects, DerSimonian and Laird method (Base Case)	0.66 (0.33 to 1.34)	2 (with 1 excluded)	-
Fixed effects to Mantel–Haenszel method	No change		
Excluding studies with high risk of attrition or allocation bias	No change		
Adding studies with CKD stage 1/2 patients	0.92 (0.80 to 1.06)	4 (1 excluded)	0%
Lipid modifying drugs			
Random effects, DerSimonian and Laird method (Base Case)	0.64 (0.52 to 0.80)	5	29.6%
Fixed effects to Mantel–Haenszel method	0.65 (0.56 to 0.76)	5	29.6%
Excluding studies with high risk of attrition or allocation bias	0.66 (0.52 to 0.83)	4	35.1%
Excluding non-ITT study (AFCAPS/TEXCAPS)	0.66 (0.52 to 0.83)	4	35.1%
Adding studies with CKD stage 1/2 patients	0.60 (0.50 to 0.72)	11	59.0%
Glycaemic control drugs			
TECOS (CKD stage 3)	0.92 (0.79 to 1.08)	1	-
**6. Cardiovascular mortality**			
Antihypertensive drugs			
Random effects, DerSimonian and Laird method (Base Case)	0.79 (0.58 to 1.07)	2	0%
Fixed effects to Mantel–Haenszel method	0.78 (0.58 to 1.07)	2	0%
Excluding studies with high risk of attrition or allocation bias	No change		
Adding studies with CKD stage 1/2 patients	0.93 (0.80 to 1.08)	7	30.0%
Lipid modifying drugs			
Random effects, DerSimonian and Laird method (Base Case)	0.52 (0.30 to 0.92)	2	0%

For peer review only

Fixed effects to Mantel–Haenszel method	0.52 (0.29 to 0.91)	2	0%
Excluding studies with high risk of attrition or allocation bias	0.53 (0.30 to 0.93)	1	-
Excluding non-ITT study (AFCAPS/TEXCAPS)	0.53 (0.30 to 0.93)	1	-
Adding studies with CKD stage 1/2 patients	0.58 (0.35 to 0.92)	5	0%
Glycaemic control drugs			
Random effects, DerSimonian and Laird method (Base Case)	0.74 (0.53 to 1.02)	2	77.8%
Fixed effects to Mantel–Haenszel method	0.74 (0.63 to 0.86)	2	77.8%
Excluding studies with high risk of attrition or allocation bias	No change		
Adding studies with CKD stage 1/2 patients	0.73 (0.57 to 0.93)	4	37.2%
**7. All-cause mortality**			
Antihypertensive drugs			
Random effects, DerSimonian and Laird method (Base Case)	0.93 (0.81 to 1.08)	3	0%
Fixed effects to Mantel–Haenszel method	0.92 (0.80 to 1.07)	3	0%
Excluding studies with high risk of attrition or allocation bias	No change		
Adding studies with CKD stage 1/2 patients	0.94 (0.87 to 1.02)	8	31.0%
Lipid modifying drugs			
Random effects, DerSimonian and Laird method (Base Case)	0.74 (0.56 to 0.98)	4	0%
Fixed effects to Mantel–Haenszel method	0.72 (0.54 to 0.95)	4	0%
Excluding studies with high risk of attrition or allocation bias	No change		
Adding studies with CKD stage 1/2 patients	0.74 (0.59 to 0.92)	9	24.6%
Glycaemic control drugs			
Random effects, DerSimonian and Laird method (Base Case)	0.88 (0.74 to 1.04)	2	32.2%
Fixed effects to Mantel–Haenszel method	No change		
Excluding studies with high risk of attrition or allocation bias	No change		
Adding studies with CKD stage 1/2 patients	0.86 (0.73 to 1.02)	7	0%

For peer review only

Supplementary file 1. MEDLINE Search Terms.

# ▲	Searches
1	renal insufficiency/ or renal insufficiency, chronic/ or exp kidney failure, chronic/
2	(chronic adj (renal or kidney*) adj2 (fail* or insufficien* or disease? or disorder? or condition?)).ti,ab.
3	(late stage adj5 (kidney* or renal)).ti,ab.
4	((stage 3 or stage 3a or stage 3b or stage iii or stage iiia or stage iiib or stage 4 or stage iv) adj5 (kidney or renal)).ti,ab.
5	1 or 2 or 3 or 4
6	exp Angiotensin-Converting Enzyme Inhibitors/
7	((angiotensin adj2 converting enzyme inhibitor?) or ace inhibitor?).ti,ab.
8	(captopril or cilazapril or enalapril or fosinopril or imidapril or lisinopril or moexipril or perindopril or quinapril or ramipril or trandolapril).ti,ab.
9	exp Angiotensin Receptor Antagonists/
10	((angiotensin adj2 receptor antagonist?) or (angiotensin adj2 receptor blocker?) or sartans).ti,ab.
11	(ace or ace-i or ace-ii or acei or aceii or arb).ti,ab.
12	(azilsartan or candesartan or eprosartan or irbesartan or losartan or olmesartan or telmisartan or valsartan).ti,ab.
13	exp Thiazides/
14	thiazide*.ti,ab.
15	(Bendroflumethiazide or Chlortalidone or chlorthalidone or Cyclopenthiazide or Indapamide or Metolazone or Xipamide or Chlorothiazide or Hydrochlorothiazide).ti,ab.
16	exp Mineralocorticoid Receptor Antagonists/
17	aldosterone antagonist?.ti,ab.
18	(eplerenone or spironolactone).ti,ab.
19	exp Adrenergic beta-Antagonists/
20	(beta* blocker? or b-blocker? or beta* adrenoceptor antagonist? or b-adrenoceptor antagonist? or beta* adrenoceptor block* or b-adrenoceptor block* or beta* adrenergic block* or b adrenergic block*).ti,ab.
21	(propranolol or acebutolol or atenolol or bisoprolol or carvedilol or celiprolol or esmolol or labetalol or metropolol or nadolol or nebivolol or oxprenolol or pindolol or sotalol or timolol).ti,ab.
22	exp Calcium Channel Blockers/
23	(calcium channel block* or calcium antagonist?).ti,ab.
24	(amlodipine or diltiazem or felodipine or isradipine or lacidipine or lercanidipine or nicardipine or nifedipine or verapamil or nimodipine or nisoldipine).ti,ab.
25	exp Adrenergic alpha-Antagonists/
26	(alpha* blocker? or a-blocker? or alpha* adrenoceptor antagonist? or a-adrenoceptor antagonist? or alpha* adrenoceptor block* or a-adrenoceptor block* or postsynaptic alpha* block* or postsynaptic a-block*).ti,ab.
27	(doxazosin or indoramin or prazosin or terazosin).ti,ab.
28	Imidazoline Receptors/ag [Agonists]
29	(imidazoline receptor agonist? or centrally acting hypertensive*).ti,ab.

For peer review only

30	moxonidine.ti,ab.
31	(clonidine or methyldopa).ti,ab.
32	exp Vasodilator Agents/
33	vasodilator?.ti,ab.
34	(hydralazine or minoxidil).ti,ab.
35	Antihypertensive Agents/
36	(antihypertensive? or anti-hypertensive? or ((antihypertensive or anti-hypertensive) adj3 (drug? or agent? or therap* or treatment))).ti.
37	((blood pressure or bp) adj3 (drug? or agent? or therap* or treatment)).ti.
38	exp Insulins/
39	insulin?.ti,ab.
40	exp Sulfonylurea Compounds/
41	sulfonylurea?.ti,ab.
42	(Glibenclamide or gliclazide or glimepiride or glipizide or tolbutamide or chlorpropamide or glibornuride).ti,ab.
43	biguanides/ or metformin/
44	(biguanide? or metformin).ti,ab.
45	exp Glycoside Hydrolase Inhibitors/
46	(alpha glucosidase inhibitor? or acarbose).ti,ab.
47	Dipeptidyl-Peptidase IV Inhibitors/
48	(Dipeptidyl-Peptidase IV Inhibitor? or Dipeptidyl-Peptidase 4 Inhibitor? or DipeptidylPeptidase IV Inhibitor? or DipeptidylPeptidase 4 Inhibitor? or Dipeptidyl-Peptidase type IV Inhibitor? or Dipeptidyl-Peptidase type 4 Inhibitor? or DipeptidylPeptidase type IV Inhibitor? or DipeptidylPeptidase type 4 Inhibitor? or gliptin?).ti,ab.
49	(alogliptin or linagliptin or saxagliptin or sitagliptin or vildagliptin).ti,ab.
50	Thiazolidinediones/
51	(Thiazolidinedione? or glitazone?).ti,ab.
52	(pioglitazone or rosiglitazone).ti,ab.
53	(meglitinides? or nateglinide or repaglinide).ti,ab.
54	(Incretin mimetic? or glucagon-like peptide-1 activator? or GLP-1 activator?).ti,ab.
55	(exenatide or liraglutide or lixisenatide).ti,ab.
56	Sodium-Glucose Transporter 2/ai [Antagonists & Inhibitors]
57	(Sodium-glucose co-transporter 2 inhibitor? or SGLT2 inhibitor?).ti,ab.
58	(canagliflozin or dapagliflozin).ti,ab.
59	Hypoglycemic Agents/
60	(glyc?emic control adj3 (drug? or agent? or therap* or treatment)).ti.
61	exp Hydroxymethylglutaryl-CoA Reductase Inhibitors/
62	((hmg coa adj2 inhibitor?) or (Hydroxymethylglutaryl-CoA adj2 Inhibitors) or statin?).ti,ab.
63	(atorvastatin or fluvastatin or pravastatin or rosuvastatin or simvastatin or cerivastatin).ti,ab.
64	bile acid sequestran?.ti,ab.
65	(colesevelam or colestyramine or colestipol).ti,ab.
66	exp Fibric Acids/
67	(fibric acid? or fibrate?).ti,ab.

For peer review only

68	(bezafibrate or ciprofibrate or fenofibrate or gemfibrozil or clofibrate).ti,ab.
69	Nicotinic Acids/
70	(nicotinic acid? or acipimox).ti,ab.
71	exp *Fatty Acids, Omega-3/
72	(omega 3 fatty acid* or omega 3 marine triglyceride*).ti,ab.
73	(ezetimibe or lomitapide).ti,ab.
74	Hypolipidemic Agents/
75	((lipid lower* or hypolipidem* or cholesterol lower* or hypocholesterol*) adj2 (drug? or agent? or therap* or treatment)).ti,ab.
76	sodium bicarbonate/
77	sodium bicarbonate.ti,ab.
78	(Chortalidone or chorthalidone or chorpropamide or canaglifozin or dapaglifozin or canaglifloxin or dapaglifloxin or canaglifoxin or dapaglifoxin or giptin? or alogiptin or linagiptin or saxagiptin or sitagiptin or vildagiptin or Eposartan or Metopolol or popranolol or popranolole or propanalol or propanalole or propanolol or propranolole).ti,ab.
79	6 or 7 or 8 or 9 or 10 or 11 or 12 or 13 or 14 or 15 or 16 or 17 or 18 or 19 or 20 or 21 or 22 or 23 or 24 or 25 or 26 or 27 or 28 or 29 or 30 or 31 or 32 or 33 or 34 or 35 or 36 or 37 or 38 or 39 or 40 or 41 or 42 or 43 or 44 or 45 or 46 or 47 or 48 or 49 or 50 or 51 or 52 or 53 or 54 or 55 or 56 or 57 or 58 or 59 or 60 or 61 or 62 or 63 or 64 or 65 or 66 or 67 or 68 or 69 or 70 or 71 or 72 or 73 or 74 or 75 or 76 or 77 or 78
80	5 and 79
81	limit 80 to "reviews (maximizes specificity)"
82	randomized controlled trial.pt.
83	controlled clinical trial.pt.
84	randomized.ab.
85	placebo.ab.
86	drug therapy.fs.
87	randomly.ab.
88	trial.ab.
89	groups.ab.
90	82 or 83 or 84 or 85 or 86 or 87 or 88 or 89
91	exp animals/ not humans.sh.
92	90 not 91
93	80 and 92
94	limit 93 to yr="1999 -Current"
95	randomized controlled trial.pt.
96	controlled clinical trial.pt.
97	randomized.ab.
98	placebo.ab.
99	clinical trials as topic.sh.
100	randomly.ab.
101	trial.ti.
102	95 or 96 or 97 or 98 or 99 or 100 or 101
103	exp animals/ not humans.sh.
104	102 not 103

For peer review only

105	80 and 104
106	limit 105 to yr="1999 -Current"

For peer review only

PRISMA 2009 Checklist

Section/topic	#	Checklist item	Reported on page #
**TITLE**			
Title	1	Identify the report as a systematic review, meta-analysis, or both.	1
**ABSTRACT**			
Structured summary	2	Provide a structured summary including, as applicable: background; objectives; data sources; study eligibility criteria, participants, and interventions; study appraisal and synthesis methods; results; limitations; conclusions and implications of key findings; systematic review registration number.	3-4
**INTRODUCTION**			
Rationale	3	Describe the rationale for the review in the context of what is already known.	5-6
Objectives	4	Provide an explicit statement of questions being addressed with reference to participants, interventions, comparisons, outcomes, and study design (PICOS).	6
**METHODS**			
Protocol and registration	5	Indicate if a review protocol exists, if and where it can be accessed (e.g., Web address), and, if available, provide registration information including registration number.	4
Eligibility criteria	6	Specify study characteristics (e.g., PICOS, length of follow-up) and report characteristics (e.g., years considered, language, publication status) used as criteria for eligibility, giving rationale.	7-8
Information sources	7	Describe all information sources (e.g., databases with dates of coverage, contact with study authors to identify additional studies) in the search and date last searched.	6-7
Search	8	Present full electronic search strategy for at least one database, including any limits used, such that it could be repeated.	5, Suppl. File 1
Study selection	9	State the process for selecting studies (i.e., screening, eligibility, included in systematic review, and, if applicable, included in the meta-analysis).	7
Data collection process	10	Describe method of data extraction from reports (e.g., piloted forms, independently, in duplicate) and any processes for obtaining and confirming data from investigators.	7
Data items	11	List and define all variables for which data were sought (e.g., PICOS, funding sources) and any assumptions and simplifications made.	8-9
Risk of bias in individual studies	12	Describe methods used for assessing risk of bias of individual studies (including specification of whether this was done at the study or outcome level), and how this information is to be used in any data synthesis.	9
Summary measures	13	State the principal summary measures (e.g., risk ratio, difference in means).	9-10
Synthesis of results	14	Describe the methods of handling data and combining results of studies, if done, including measures of consistency (e.g., I^2) for each meta-analysis.	9-11

For peer review only

PRISMA 2009 Checklist

Section/topic	#	Checklist item	Reported on page #
Risk of bias across studies	15	Specify any assessment of risk of bias that may affect the cumulative evidence (e.g., publication bias, selective reporting within studies).	11
Additional analyses	16	Describe methods of additional analyses (e.g., sensitivity or subgroup analyses, meta-regression), if done, indicating which were pre-specified.	10-11
**RESULTS**			
Study selection	17	Give numbers of studies screened, assessed for eligibility, and included in the review, with reasons for exclusions at each stage, ideally with a flow diagram.	12-13, Figure 1
Study characteristics	18	For each study, present characteristics for which data were extracted (e.g., study size, PICOS, follow-up period) and provide the citations.	13, Suppl. Tables 2, 3, 4
Risk of bias within studies	19	Present data on risk of bias of each study and, if available, any outcome level assessment (see item 12).	14, Suppl. Figure 2
Results of individual studies	20	For all outcomes considered (benefits or harms), present, for each study: (a) simple summary data for each intervention group (b) effect estimates and confidence intervals, ideally with a forest plot.	14-19, Figures 2-7, Suppl. Table 5
Synthesis of results	21	Present results of each meta-analysis done, including confidence intervals and measures of consistency.	14-19, Figures 2-7, Suppl. Table 5
Risk of bias across studies	22	Present results of any assessment of risk of bias across studies (see Item 15).	14, Suppl. Figure 2
Additional analysis	23	Give results of additional analyses, if done (e.g., sensitivity or subgroup analyses, meta-regression [see Item 16]).	14-19, Figures 2-7, Suppl. Table 5
**DISCUSSION**			
Summary of evidence	24	Summarize the main findings including the strength of evidence for each main outcome; consider their relevance to key groups (e.g., healthcare providers, users, and policy makers).	19, 21
Limitations	25	Discuss limitations at study and outcome level (e.g., risk of bias), and at review-level (e.g., incomplete retrieval of identified research, reporting bias).	20-21
Conclusions	26	Provide a general interpretation of the results in the context of other evidence, and implications for future research.	19-21
**FUNDING**			
Funding	27	Describe sources of funding for the systematic review and other support (e.g., supply of data); role of funders for the systematic review.	22-23

For peer review only - http://bmjopen.bmj.com/site/about/guidelines.xhtml

PRISMA 2009 Checklist

From: Moher D, Liberati A, Tetzlaff J, Altman DG, The PRISMA Group (2009). Preferred Reporting Items for Systematic Reviews and Meta-Analyses: The PRISMA Statement. PLoS Med 6(7): e1000097. doi:10.1371/journal.pmed1000097

For more information, visit: **www.prisma-statement.org**.

For peer review only

BMJ Open

Effects of antihypertensives, lipid-modifying drugs, glycaemic control drugs and sodium bicarbonate on the progression of stages 3 and 4 chronic kidney disease in adults: a systematic review and meta-analysis

Journal:	*BMJ Open*
Manuscript ID	bmjopen-2019-030596.R1
Article Type:	Original research
Date Submitted by the Author:	13-Aug-2019

Complete List of Authors:	Taylor, Kathryn; University of Oxford, Nuffield Department of Primary Care Health Sciences<br>Mclellan, Julie; University of Oxford, Nuffield Department of Primary Care Health Sciences<br>Verbakel, Jan; University of Oxford, Nuffield Department of Primary Care Health Sciences; KU Leuven, Department of Public Health and Primary Care<br>Aronson, Jeffrey; University of Oxford, Nuffield Department of Primary Care Health Sciences<br>Lasserson, Daniel; University of Birmingham, Institute of Applied Health Research; University of Oxford, Nuffield Department of Medicine<br>Pidduck, Nicola; University of Oxford, Nuffield Department of Primary Care Health Sciences<br>Roberts, Nia; University of Oxford, Bodleian Health Care Libraries<br>Fleming, Susannah; University of Oxford, Nuffield Department of Primary Care Health Sciences<br>O'Callaghan, Christopher; University of Oxford, Nuffield Department of Clinical Medicine; Oxford Radcliffe Hospitals Trust, John Radcliffe Hospital<br>Bankhead, Clare; University of Oxford, Nuffield Department of Primary Care Health Sciences<br>Banerjee, Amitava; University College London, Farr Institute of Health Informatics Research<br>Hobbs, Richard; University of Oxford, Nuffield Department of Primary Care Health Sciences<br>Perera, Rafael; University of Oxford, Nuffield Department of Primary Care Health Sciences

**Primary Subject Heading**:	Renal medicine
Secondary Subject Heading:	General practice / Family practice
Keywords:	Chronic renal failure < NEPHROLOGY, Cardiovascular events, Meta-analysis, Renal function, Drug treatment

SCHOLARONE™
Manuscripts

Effects of antihypertensives, lipid-modifying drugs, glycaemic control drugs, and sodium bicarbonate on the progression of stages 3 and 4 chronic kidney disease in adults: a systematic review and meta-analysis

Kathryn S Taylor[1], Julie McLellan[1], Jan Y Verbakel[1,2], Jeffrey K Aronson[1], Daniel S Lasserson[3], Nicola Pidduck[1], Nia Roberts[4], Susannah Fleming[1], Christopher A O'Callaghan[5], Clare Bankhead[1], Amitava Banerjee[6], F D Richard Hobbs[1], Rafael Perera[1].

[1] Nuffield Department of Primary Care Health Sciences, University of Oxford, OX2 6GG, United Kingdom

[2] Department of Public Health and Primary Care, KU Leuven, Belgium

[3] Institute of Applied Health Research, College of Medical and Dental Sciences, Murray Learning Centre, University of Birmingham, Edgbaston, Birmingham, B15 2TT, United Kingdom.

[4] Knowledge Centre, ORC Research Building, Old Road Campus, Oxford, OX3 7DQ

[5] Nuffield Department of Medicine, University of Oxford, Henry Wellcome Building, Roosevelt Drive, Oxford OX3 7BN, United Kingdom

[6] Health Data Research UK-London, Institute of Health Informatics, University College London, 222 Euston Road London, NW1 2DA, United Kingdom

Kathryn S Taylor, Medical Statistician

Julie McLellan, Senior Systematic Reviewer

Jan Verbakel, Senior Clinical Researcher and Tenure Track Professor

Jeffrey K Aronson, Consultant Physician and Clinical Pharmacologist

For peer review only - http://bmjopen.bmj.com/site/about/guidelines.xhtml

Daniel Lasserson, Professor of Ambulatory Care

Nicola Pidduck, Project Manager and Systematic Reviewer

Nia Roberts, Librarian

Susannah Fleming, Quantitative Researcher

Christopher A O'Callaghan, Professor of Medicine and Honorary Consultant Physician and Nephrologist

Amitava Banerjee, Senior Clinical Lecturer in Clinical Data Science and Honorary Consultant Cardiologist

F D Richard Hobbs, Professor of Primary Care Health Sciences

Rafael Perera, Professor of Medical Statistics

Correspondence to: Julie McLellan, Nuffield Department of Primary Care Health Sciences, University of Oxford, OX2 6GG, United Kingdom, julie.mclellan@phc.ox.ac.uk, 01865 289667

Word Count: 5145

For peer review only

For peer review only - http://bmjopen.bmj.com/site/about/guidelines.xhtml

ABSTRACT

Objectives To evaluate the effects of drug interventions that may modify the progression of chronic kidney disease (CKD) in adults with CKD stages 3 and 4.

Design Systematic review and meta-analysis.

Methods Searching MEDLINE, EMBASE, DARE, CENTRAL, CDSR, ICTRP, HTA, SCI, SSCI, CPCI and Clinical Trials Register, from March 1999 to July 2018, we identified **r**andomised controlled trials (RCTs), of drugs for hypertension, lipid modification, glycaemic control, and sodium bicarbonate, compared with placebo, no drug, or a drug from another class, in ≥40 adults with CKD stages 3 and/or 4, with at least two years follow-up and reporting renal function (primary outcome), proteinuria, adverse events, maintenance dialysis, transplantation, cardiovascular events, cardiovascular mortality or all-cause mortality. Two reviewers independently screened citations and extracted data.. For continuous outcomes, we used the ratio of means (ROM) at the end of the trial in random-effects meta-analyses. We assessed methodological quality with the Cochrane Risk of Bias Tool and confidence in the evidence using the Grading of Recommendations Assessment, Development and Evaluation (GRADE) framework.

Results We included 35 RCTs and over 51,000 patients. Data were limited. Heterogeneity varied. Final renal function (estimated glomerular filtration rate) was 6% higher in those taking glycaemic control drugs (ROM 1.06, 95% CI 1.02 to 1.10, I^2=0%, low GRADE confidence) and 4% higher in those taking lipid-modifying drugs (ROM 1.04, 1.00 to 1.08, I^2=88%, very low GRADE confidence). For RCTs of antihypertensive drugs, there were no significant differences in renal function. Treatment with lipid-modifying drugs led to a 36% reduction in cardiovascular disease and 26% reduction in all-cause mortality.

For peer review only - http://bmjopen.bmj.com/site/about/guidelines.xhtml

Conclusions Glycaemic control and lipid-modifying drugs may slow the progression of CKD, but we found no evidence of benefit nor harm from antihypertensive drugs. However, given the data limitations, further research is needed to confirm these findings.

PROSPERO registration number CRD42015017501

ARTICLE SUMMARY

Strengths and limitations of this study

- By focusing on patients with CKD stage 3 and 4, we provide a primary care perspective on the management of CKD.
- We completed an extensive and comprehensive search including common drug misspellings, with no restriction on language or publication type.
- Generalisability of our findings is limited by the paucity of trials, high statistical heterogeneity, high clinical heterogeneity, in terms of co-morbidities, and by the effect of drugs on CKD being secondary in all the studies.
- Lack of data restricted our ability to explore the baseline characteristics of studies, the effects of drugs, the link between treatment effect and drug dose, and assess the potential for publication bias.

For peer review only

For peer review only - http://bmjopen.bmj.com/site/about/guidelines.xhtml

INTRODUCTION

Chronic kidney disease (CKD) is long-term loss of renal function and is a global health problem, with an estimated prevalence of 11-13%.[1] CKD is an independent risk factor for cardiovascular disease,[2] and people with mild-to-moderate CKD are at increased risk of mortality, and progression to renal failure.[3]

Historically, CKD was categorised by its cause, which was predominantly diabetes, hypertension, glomerulonephritis, or polycystic kidney disease. Treatment could be recommended, but often opportunities were missed for early detection of the disease or prevention of clinical complications and further deterioration. The National Kidney Foundation, addressed this challenge by developing a five-stage classification system based on the glomerular filtration rate (GFR) (supplementary material), [4] which was subsequently modified to take into account degrees of proteinuria (supplementary material).[5] A UK audit in 2012 estimated that 90% of cases of CKD are at stage 3, and of these, 84% are stage 3a (eGFR 45-59 ml/min/1.73^2).[6]

Patients with CKD who are managed in primary care include those in stages 1 and 2, with an emphasis on lifestyle and diet changes. Patients in stages 3 and 4 may be managed either in primary care or shared care with specialist nephrology services, with a greater use of medication, primarily for associated long-term conditions, such as diabetes and hypertension. Both published data for Oxfordshire[7] and unpublished national data from our group show that some patients with CKD stage 4 have their renal function monitored in primary care.

Four different classes of drugs may modify CKD progression: antihypertensives,[8] lipid-modifying drugs,[9] glycaemic control medications in patients with diabetes,[10] and sodium bicarbonate.[11-13] Sodium bicarbonate addresses metabolic acidosis, a prevalent complication in moderate and late-stage CKD, and may offer a simple and low-cost treatment to slow CKD progression. The effects of

For peer review only - http://bmjopen.bmj.com/site/about/guidelines.xhtml

drugs on CKD are secondary, as these drugs are primarily used to treat hypertension, hypercholesterolemia, or diabetes.

Systematic reviews have been compiled to summarise the effects of drug interventions on the rate of CKD progression.[9 10 14-16] However, while two have reported results based on estimated GFR (eGFR), none have reported results with reference to CKD staging. Furthermore, no systematic review has compared the effects of different drug classes.

Our objective was to carry out a systematic review and meta-analysis of randomised controlled trials, to examine and compare the effects on the progression of CKD of the four classes of drugs, focusing on patients who can be managed in primary care or shared care with specialist nephrology services.

METHODS

Our systematic review protocol was registered with PROSPERO and reported in line with recommendations from the PRISMA statement for reporting systematic reviews and meta-analyses of studies that evaluate healthcare interventions (PRISMA[17]).

Search strategy

The search strategy was designed with advice from an information specialist (NR) and clinicians including nephrologists. MeSH terms included terms for population, study design, and intervention drugs (randomised control trials, chronic kidney, renal, drug classes, and specific drug names, including common misspellings). Our included drug list is shown in the supplementary material.

For peer review only - http://bmjopen.bmj.com/site/about/guidelines.xhtml
For peer review only

Searches were conducted in Ovid MEDLINE (supplementary material), the Cochrane Controlled Register of Controlled Trials (CENTRAL), the Cochrane Database of Systematic Reviews (CDSR), Database of Abstracts of Reviews of Effects (DARE), Embase, Health Technology Assessment (HTA), Science Citation Index (SCI), Social Science Citation Index (SSCI), Conference Proceedings Citation Index (CPCI), Clinical Trials Register, and the World Health Organization International Clinical Trials Platform (ICTRP) to 6 July 2018 to identify relevant studies, regardless of their publication type. No language restriction was applied. All publications from March 1999 onwards were considered, to align with the publication of the first equation to calculate eGFR, which had led to the introduction of CKD staging.[18] Reference lists were also examined.

Study selection

Two reviewers (KST, JM, NP and SF; in pairs) reviewed the title and abstract of each reference and identified potentially relevant references. Considering the full texts of these studies, two reviewers independently selected studies to be included in the review using predetermined inclusion criteria. Disagreements about study inclusion were resolved by a third reviewer.

Our study inclusion criteria were:

(1) Randomised controlled trials of drugs that may modify the progression of CKD, antihypertensives, lipid-modifying drugs, glycaemic control drugs, and sodium bicarbonate

(2) Control arms were either given a placebo drug, no drug intervention, or a comparator drug from the three other classes

(3) Adult participants

(4) Patients with stage 3 and/or stage 4 CKD. Studies additionally including patients with stage 1 and/or stage 2 CKD were also included to increase the number of potential studies, but we would expect the treatment effect to be overestimated in these studies, as in very advanced

For peer review only - http://bmjopen.bmj.com/site/about/guidelines.xhtml

disease, the response to treatment may be attenuated by the severity of associated physiological changes.

(5) Populations with at least 40 participants

(6) Follow-up of at least two years

(7) Publications reporting a quantitative summary of effect as a change from baseline, or an endpoint measure of at least one of seven pre-specified outcomes

   i. Renal function, which could be measured by GFR, eGFR, creatinine clearance (CrCl) or estimated CrCl (eCrCl). This was our primary outcome of interest.
   ii. Proteinuria, which could be measured by protein excretion rate (PER), protein creatinine ratio (PCR), albumin excretion rate (AER) or albumin creatinine ratio (ACR).
   iii. Adverse events
   iv. Commencement of maintenance dialysis or kidney transplantation
   v. Cardiovascular events
   vi. Cardiovascular mortality
   vii. All-cause mortality

We excluded trials that reported only data about populations that included people with CKD stage 5, to avoid underestimating the treatment effect, as we would expect stage 5 patients to have more co-morbidities, which might lead to worse outcomes.

Data extraction and quality assessment

Data were extracted independently by two reviewers (KST, JM, NP and JV; in pairs) using a data extraction form, which was piloted on a sample of five studies.

For peer review only - http://bmjopen.bmj.com/site/about/guidelines.xhtml

For peer review only

Extracted data included study details (setting, intervention dose and frequency, follow-up period) information on study participants (age, gender, ethnicity, smoking status, blood pressure, CKD stage, existing co-morbidities) and outcome data. For one study[19 20] we extracted endpoint eGFR data from an earlier point than the longest follow-up as the patient numbers at the longest follow-up were so small (<7% of the population). We contacted study authors to seek clarification about data and to request data that could be included in our study.

The methodological quality of studies was assessed independently by two reviewers (KST, JM, NP and JV; in pairs) using the Cochrane Risk of Bias tool.[21] This tool considers the method of random sequence generation and allocation concealment (selection bias), blinding of participants, personnel and outcomes (performance and detection biases), the levels and balance of missing outcome data (attrition bias), and completeness of reporting (selective reporting bias).

Disagreements about extracted data or methodological assessments were resolved by a third reviewer.

Data synthesis and analysis

We expected high statistical heterogeneity,[22] but pooled data to give an indication of the average effect. We also pooled data when there were only two or three studies, although this could be potentially misleading, in order to give an indication of potential trends had there been more study data. We carried out random-effects analyses based on the DerSimonian and Laird method[23] and assessed statistical heterogeneity using the I-squared statistic and calculated approximate 95% prediction intervals to estimate the likely effects in a clinical setting[24] using the methods of Higgins et al.[25]

For peer review only - http://bmjopen.bmj.com/site/about/guidelines.xhtml

For the continuous outcomes (renal function and proteinuria), we used a ratio of means (ROM)[26 27] effect measure, which allowed us to combine data from trials that reported different measures of renal function or proteinuria in a single analysis. Using the ROM effect measure overcomes limitations of the well-established standardised mean difference[21], by providing units that are more easily interpreted than standard deviations (SDs), and avoiding the problems associated with presenting results with SDs, which can produce misleading results by deflating treatment effects for studies of heterogeneous populations and inflating treatment effects in homogeneous populations[28] We calculated the ROM as the ratio of the final eGFR in the intervention group to the final eGFR in the comparator group, so ROM>1 favoured the intervention group for eGFR, where higher values are better than lower values. If a study of renal function reported reductions from baseline or rates of decline rather than endpoint data, we estimated the ROM of endpoints by the ratio of the reduction from baseline or rate of decline in the comparator group to that in the intervention group (the reciprocal) to ensure the direction of effect was consistent across all studies, as higher values of declining renal function are worse. ROM<1 favoured the intervention group for the proteinuria outcomes (ACR, AER, PER, PCR), where higher values are worse.

For a few studies it was necessary to make further estimates by: approximating a mean using the median; estimating a standard deviation from the interquartile range;[21] or estimating the standard error of the ROM by imputation using the mean value of the known standard errors of the other studies.

Meta-analysis was carried out on the log-transformed ROM and its standard error. For the primary analysis, eGFR measured by the Modification of Diet in Renal Disease (MDRD) equation was selected over Chronic Kidney Disease Epidemiology Collaboration (CKD-EPI) data, as MDRD is the main equation advocated in clinical guidelines and CKD-EPI was not introduced until 2009;[5 29] eGFR was also selected over CrCl. When analysing the effects of treatments on proteinuria we pooled data for PER,

For peer review only - http://bmjopen.bmj.com/site/about/guidelines.xhtml

PCR, ACR, and AER by using a ROM effect measure. For the dichotomous outcomes, we specified the relative risk as the effect measure.

We performed separate sensitivity analyses to ensure that our estimates of the effect of treatment in studies of patients with CKD stages 3 and 4 only (our main focus) were not sensitive to our choice of model, assumptions, the quality of the studies, the estimations made, or our choices of data.

Pre-specified sensitivity analyses were

1) Fixed-effect analyses, based on the inverse-variance method were carried out for the continuous outcomes, and based on the Mantel–Haenszel method for the dichotomous outcomes.[30 31]
2) Excluding studies rated as low quality, which we defined as having a high risk of attrition bias or allocation concealment bias; studies rated as 'unclear' were not excluded.
3) Excluding studies without intention-to-treat analysis. This was pre-specified on the assumption that we would be dealing with primary studies of the progression of CKD but as the studies were secondary or post-hoc this analysis was no longer relevant.

A further, standard set of sensitivity analyses were

4) Excluding studies with estimates.
5) Using more conservative estimates, considering those of the standard error of the ROM by imputing with the 75% percentile value of the known standard errors rather than the mean value, reflecting greater uncertainty.
6) Using alternative data, considering CKD-EPI data instead of MDRD data, for studies that measured eGFR using both equations.
7) Excluding studies that measured PCR and PER in the analysis of proteinuria, as urine albumin is a more accurate measure of glomerular damage.
8) Excluding each individual study, one-by-one, to see if the level of heterogeneity depended on a particular study.

For peer review only - http://bmjopen.bmj.com/site/about/guidelines.xhtml

Post-hoc sensitivity analyses were

9) Using the Hartung-Knapp-Sidik-Jonkman variance correction to calculate 95% confidence intervals reflecting the uncertainty in heterogeneity estimates.[32-34]
10) Excluding studies with non-placebo controls

Pre-specified subgroup analyses were conducted by age (<65 or ≥65 years), ethnicity, smoking status, intensity of intervention, high blood pressure (≥140/90mmHg), low blood pressure (≤90/60mmHg), method of eGFR measurement, degree of proteinuria in the renal function analysis, drug type within each drug class, and by CKD stages 3 and 4 separately. Heterogeneity was assessed using the I^2 statistic. Publication bias was explored using funnel plot analysis with contour plots.[21 35]

We used the Grading of Recommendations, Assessment and Evaluation (GRADE) framework[21] to report the overall quality of evidence for the primary outcome (final eGFR). The certainty in the evidence for each outcome was graded as high, moderate, low or very low.

When describing the included trials, we refer to "studies" to encompass single trials and publications that report more than one trial. When reporting analyses of the effects of the drugs, we refer to populations as some studies reported data for CKD subgroups or dose-dependent subgroups. All results of meta-analysis are displayed in forest plots and/or tabulated. Results are divided into those for studies of patients with CKD stages 3 and 4 only and studies of patients with CKD stages 3 and 4 mixed with patients with stages 1 and 2.

All analyses were carried out using STATA v14.2[36] except the risk of bias figures which were created in RevMan v 5.3.[37]

Patient involvement

For peer review only - http://bmjopen.bmj.com/site/about/guidelines.xhtml

Members of a patient and public involvement group were part of the Stakeholder Group and Steering Committee of the National Institute for Health Research (NIHR) Programme Grant that inspired this study. No patients or patient representatives were involved in setting the outcome measures, nor were they directly involved in developing plans for the design or implementation of the study. Three members of this group commented on our manuscript and we thank them for their help in our acknowledgement section. A one-day dissemination event is planned, to report the results of all the studies funded by the NIHR Programme Grant, including this study. Members of the patient and public involvement group will be invited to this event.

RESULTS

Search results

Through database registry and hand searches we identified 13,133 records (Figure 1), of which 35 studies (51,155 patients) satisfied our eligibility criteria.

Study characteristics

Most of the 35 studies reported data about a single trial while three studies reported data about a pooled analysis of more than one trial.[19 20 38-43] Of the 35 studies, 1 from China,[44] 1 from Tasmania,[45] 3 from Japan,[46-51] 6 from the USA/Canada,[52-65] 9 from Europe,[13 66-78] either in single or multiple countries, and the other 15 studies were multinational (supplementary material). Nineteen studies[13 19 20 38-40 44 48-57 61-65 68-70 74 75 78-90] provided data for populations with CKD stages 3 and/or 4 only. These studies had between 108 and 3094 patients and the mean or median follow-up was between 24 and 66 months.

For peer review only - http://bmjopen.bmj.com/site/about/guidelines.xhtml

One study[67] had a 2x2 factorial design involving an antihypertensive drug and a lipid-modifying drug and this study is included in our analysis of both antihypertensive drugs and lipid-modifying drugs. Another study had a 2x2[71 72] factorial design involving two antihypertensive drugs and a lipid-modifying drug, and this study is only included in our analysis of lipid-modifying drugs because all patients received antihypertensives. The other 33 studies were 12 studies of antihypertensives,[16 38-40 44 46 47 52-57 66 67 79-83 91-98] 13 of lipid-modifying drugs,[45 48-51 58-63 68-70 73-78 84-86 99-104] 6 of glycaemic control agents[19 20 41-43 87-90 105-107] and 2 of sodium bicarbonate[13 64 65] (supplementary material). Four studies[45 58-60 73 102-104] had populations with CKD and no comorbidities highlighted, 27 studies had a single co-morbidity and 4 studies had more than one comorbidity. Co-morbidities were cardiovascular in 19 studies;[19 20 38-43 46-51 53 54 57 61-63 68-72 74-78 81-83 89-92 98-101] type II diabetes in 9 studies[19 20 41-43 53-56 79 80 87-90 93-97 105-107] (including all 6 studies of glycaemic control drugs); and a renal disorder or damage (idiopathic chronic glomerulonephritis, advanced chronic renal insufficiency without diabetes, advanced autosomal dominant polycystic kidney disease, renal impairment and metabolic acidosis) in 7 studies[44 52 64-67 84-86 108] (supplementary material). In 15 studies,[13 45-47 61-67 71-73 76 77 91-99 107] the population analysed for our primary outcome was the whole trial population and in the other 20 the population analysed was a subgroup of the trial population, reported as either a subgroup or post-hoc analysis.(supplementary material).

Baseline data were provided for the same population as that included in our analyses for 19 of the 35 studies;[13 45 49-51 58-70 73 76-80 84-88 91-98 107] for the other 16 studies, baseline data were only provided for either a different subset of the trial population or the whole trial population (supplementary material).

Risk of bias

We considered the methodological quality of all 35 eligible studies (supplementary material), based on the design of the trial(s) from which data were obtained. Eight studies[19 20 44 71-73 84-88 98 105 106] were

For peer review only - http://bmjopen.bmj.com/site/about/guidelines.xhtml

ranked with high quality as they had 'Low' risk of bias across all domains and we rated one study[76 77] as 'High' risk of bias for three domains and 'Unclear' across two domains. Most studies described their method of randomisation, but fewer studies were clear about how they concealed the group allocation and whether participants, personnel and outcome assessors were blinded. The risk of attrition bias was high in nearly a quarter of the studies.

We had sufficient data to consider the possibility of publication bias based on the primary outcome of renal function in those taking antihypertensive and lipid-modifying drugs. The contour-enhanced funnel plots suggested possible publication bias, as few studies were in the area of non-significance (supplementary material).

Renal function

Most of the available data were on eGFR.

The pooled ratio of means of eGFR for antihypertensive drugs versus comparators at the end of the studies is shown in Figure 2. There was no significant effect in studies of populations with CKD stages 3 and 4, and heterogeneity between studies was substantial at 94% (yielding a prediction interval of 0.79 to 1.34) and this was not attributable to a single study. Adding data from the studies of patients that included those with CKD stages 1 and/or 2 increased the benefit to the intervention group but not significantly. All except one study (Bianchi 2010[66]) had placebo controls. All the sensitivity analyses produced similar results of no significant treatment effect and heterogeneity remained substantial (supplementary material).

The only subgroup analysis that was possible, of those pre-specified, was by drug type within the antihypertensive drug class. Among studies of CKD stages 3 and 4 only, there were no significant

For peer review only - http://bmjopen.bmj.com/site/about/guidelines.xhtml

treatment effects within the different drug types (supplementary material), except in the case of calcium channel blockers, where the endpoint eGFR was 14% higher in the intervention group (ROM 1.14, 95% confidence interval 1.01 to 1.27), but this was based on a single study.[53 54]

In studies of patients with CKD stages 3 and 4 there was a marginally higher benefit to those taking the lipid-modifying drugs (Figure 3), as the endpoint eGFR was 4% higher (ROM 1.04, 1.00 to 1.08, statistically significant, I^2=88, STATA's metaninf command produced output that indicated that the lower confidence bound was 1.0001 to 4 d.p.) with a prediction interval of 0.91 to 1.18. This result was not robust as the results of sensitivity analysis were variable (supplementary material). The high degree of heterogeneity was attributed to the SHARP study[102-104] of the CKD stage 4 subgroup, as removing this study reduced the I^2 from 88.3% to 45.3% . The intervention for this study was a higher dose of statin than in the other studies, and another lipid modifying drug, ezetimibe, was also used. Adding data from the studies of patients including those with CKD stages 1 and/or 2 increased the benefit to the intervention group but not significantly and there was substantial heterogeneity between studies (I^2=98%). Three trials had non-placebo controls (ASUCA[48], MEGA[49-51] and GREACE[76 77]).

There was a significant benefit to those taking glycaemic controlling drugs (Figure 4) as the mean eGFR at the end of the study was 6% higher in the intervention group (ROM 1.06, 1.02 to 1.10). Heterogeneity between studies was low (I^2=0%) yielding a prediction interval of 1.01 to 1.11. Sensitivity analyses showed that this result was robust (supplementary material). Adding the CKD stage 1 to 4 studies, there was no significant difference between the intervention and comparator groups and the heterogeneity between studies was high (I^2=85%). In all studies the comparator groups received placebo drugs. We assessed the outlier[41-43] as having high attrition bias as of the 1887 who were randomised, only 487 completed the 104 day study period. After excluding this study, there was

For peer review only - http://bmjopen.bmj.com/site/about/guidelines.xhtml

a significant benefit to the intervention group, which was similar to that for the studies of CKD stage 3 and 4 patients.

A study[64 65] of patients with CKD stage 3 reported a 22% higher endpoint eGFR from taking sodium bicarbonate (ROM 1.22, 1.10 to 1.36). Another study[13] of patients with CKD stage 4, reported that taking sodium bicarbonate produced no significant effect on renal function in terms of creatinine clearance (ROM 0.94, 0.85 to 1.05). Pooling these data, there was no significant treatment effect, based on renal function (supplementary material). In both studies the comparator groups received routine care.

Figure 5 summarises the effects of treatment on renal function by drug class for the four drug classes. The GRADE confidence in these estimates was very low for our analysis of antihypertensive drugs, lipid regulating drugs and sodium bicarbonate, and low for our analysis of glycaemic control drugs.

Proteinuria

Data pooling for proteinuria was possible only for antihypertensive drugs in five studies. For studies of CKD stages 3 and 4 and overall, there was no significant treatment effect (supplementary material), with ROM 0.91 (0.78 to 1.05) and low heterogeneity at 41.3%. All sensitivity analyses produced the same result of no treatment effect with low heterogeneity (supplementary material).

Two studies of lipid-modifying drugs reported proteinuria. The ATIC study[73] of patients with CKD stages 2 to 4 reported that treatment reduced urinary albumin excretion and provided medians and ranges of the AER (77, 3 to 2509 mg/24 hours for the intervention group, and 107, 5 to 3545 mg/24 hours for the comparator group). The PREVEND IT lipid study[67] of patients with CKD stages 1 to 3 reported that treatment slightly increased urinary albumin excretion by quoting medians and

For peer review only - http://bmjopen.bmj.com/site/about/guidelines.xhtml

interquartile ranges (21.8 and 11.6 to 41.9 mg/24 hours for the intervention group, and 20.3 and 12.5 to 40.5 mg/24 hours for the control group).

One glycaemic control study reported proteinuria as an outcome. The CANVAS program[19 20] reported that treatment significantly reduced albuminuria compared with the placebo group. ACR was expressed on the geometric mean scale, as 13% lower in the intervention group compared with the controls (95% CI 1% to 24%) in patients with CKD stage 3a, and 26% lower (20% to 31%) in patients with CKD stages 3b and 4.

One sodium bicarbonate study[64 65] reported that treatment lowered the ACR by quoting median and interquartile range 257.1 (205.5 to 305.0 mg/g Cr) for the intervention group and 300.3 (241.8 to 347.5 mg/g Cr) for the control group.

Adverse events

There were no significant differences in the risk of adverse events in the studies of antihypertensives, lipid-modifying drugs and glycaemic control agents (supplementary material). Neither of the bicarbonate studies reported adverse events.

Maintenance dialysis and kidney transplantation

Two studies of antihypertensive drugs reported the number of patients who deteriorated and required maintenance dialysis or kidney transplantation. The risk in the intervention group was 31% lower than in the control group in the HALT PKD study[52] of patients with stages 3 and 4 CKD (relative risk 0.69, 95% CI 0.50 to 0.96). In the RENAAL study[93-97], which was based on patients with CKD stages 2 to 4, treatment with antihypertensives led to a 23% reduction in risk of deterioration to kidney

For peer review only - http://bmjopen.bmj.com/site/about/guidelines.xhtml

For peer review only

failure (RR 0.77, 0.64 to 0.93). There were no significant differences in this outcome in the studies of lipid-modifying drugs or glycaemic control drugs (supplementary material).

Cardiovascular events

There were no significant differences between the intervention and control groups in the risk of cardiovascular events in the studies of antihypertensives whereas treatment with lipid-modifying drugs led to a reduction in risk of 36% (RR 0.64, 0.52 to 0.80, I^2= 30%) (Figure 6). All the sensitivity analyses yielded similar results and heterogeneity generally remained low (supplementary material). The reduction in risk was slightly higher when data were added from the studies of patients that included those with CKD stages 1 and/or 2, but this reduction in risk was not significant (supplementary material).

The TECOS glycaemic control study[89 90] reported the number of patients who experienced cardiovascular events in patients with CKD stage 3 patients. They reported no significant differences between the intervention and control groups (supplementary material).

Mortality

In both our analyses of the studies of antihypertensive drugs and glycaemic control drugs we found no significant differences, between the intervention and comparator groups, in the risk of cardiovascular mortality and all-cause mortality (supplementary material).

Two studies reported the effect of lipid-modifying drugs on the risk of cardiovascular mortality in patients with CKD stages 3 and 4. In the FIELD study[84-86], the risk was 47% lower in the intervention

For peer review only - http://bmjopen.bmj.com/site/about/guidelines.xhtml

group (RR 0.53, 0.30 to 0.92), and in the AFCAPS/TEXCAPS study[58-60], there was no significant difference. The latter study was not based on an intention-to-treat analysis.

In the studies of patients with CKD stage 3 and 4 only, treatment with lipid-modifying drugs reduced the risk of all-cause mortality by 26% (RR 0.74, 0.56 to 0.98) (Figure 7 and supplementary material).

DISCUSSION

Main findings

We found limited data on the effects of antihypertensive, lipid-modifying, and glycaemic control drugs and sodium bicarbonate on the progression of renal function in patients with CKD stages 3 and 4. There was heterogeneity of patient populations in the included studies. Pooling data provided no evidence of benefit or impairment of renal function in these patients attributable to antihypertensive drugs. There was some suggestion of benefit to renal function from taking glycaemic control drugs and lipid-modifying drugs, and a clear reduction in cardiovascular disease and all-cause mortality by taking lipid-modifying drugs. There was insufficient evidence to draw any conclusions about sodium bicarbonates. There was only sufficient data to study the effects of antihypertensive drugs on proteinuria and we found no significant treatment effect.

Comparison with other reviews

Our review is comparable to two reviews of drug interventions in patients with CKD, as they report changes in eGFR as outcome measures. Our findings on the protective effect of lipid-modifying drugs is consistent with the findings of Sandhu et al,[9] who concluded that statins produce a small reduction

For peer review only - http://bmjopen.bmj.com/site/about/guidelines.xhtml

For peer review only

in the progression of CKD in patients with cardiovascular disease. Our results are also consistent with those of Lewis et al[16] who reported the preliminary results of a review that considered the effect on kidney function of lowering low density lipoprotein cholesterol with statins. Their preliminary findings suggested that for patients taking statins there was a highly significant reduction in the annual rate of CKD progression compared with control groups (based on the annual rate of change in eGFR). Both of these studies considered general populations with CKD. Our finding that lipid-modifying drugs reduce the risk of cardiovascular events and all-cause mortality also concurs with other reviews of patients with CKD.[109-111]

Strengths and limitations

Our study is a first attempt to summarise the literature on drug treatment for patients with CKD types 3 and 4. We have identified, evaluated, and compared the effects of different drug interventions in reducing the rate of CKD progression in patients with CKD stages 3 and 4. By focusing on this patient group, we took a primary care perspective on the management of CKD. Our search was extensive and comprehensive, with no restrictions on language or publication type. By using the ROM as the effect measure for continuous outcomes we were able to pool data on different measures in a single analysis and overcome the limitations of the more established SMD. This provided an overall indication of the treatment effect, as did pooling data in cases where there were few studies or a high degree of heterogeneity. The generalisability of our findings is limited by the paucity of studies, the high degree of statistical heterogeneity, and clinical heterogeneity in terms of co-morbidities and treatment interventions. We concluded that clinical heterogeneity was inevitable in studies in these patients, as most were undergoing treatment with background medications. Paucity of data restricted our ability to explore the baseline characteristics of studies and the effects of lipid-modifying drugs, glycaemic control drugs and bicarbonates, particularly on proteinuria and our other secondary outcomes. We were also restricted in our ability to investigate the link between treatment effect and drug dose and

For peer review only - http://bmjopen.bmj.com/site/about/guidelines.xhtml

assess publication bias fully. There was a wide variation of study characteristics (in terms of population size and follow up period) and baseline characteristics (in terms of male/female split, smoking status, ethnicity, and hypertensive status). The generalisability of the findings is also limited by the effect of drugs on CKD being secondary in all the studies. For example, antihypertensives target hypertension and glycaemic control drugs target diabetes. Our ability to assess the quality of the included studies was restricted, because for nearly half the studies, we could not assess allocation concealment bias, and for around a quarter of the studies we could not ascertain the risk of attrition bias or whether the participants, personnel and outcomes assessors had been blinded. Further bias may have arisen as most of the reports in our included studies were subgroup or post-hoc analyses of trials. The balance achieved by randomising the whole trial population may not apply to subgroups of patients. However, we were unable to check this as few of these studies reported the necessary baseline characteristics, split by intervention group. Few studies reported the use of maintenance dialysis and kidney transplantation, which may be considered to be more appropriate survival outcomes for patients with CKD stages 3 and 4, compared with mortality. Limitations may also arise from the possible impact on patient management decisions following the introduction of CKD staging in 2002. Before that, patients were treated on the basis of the perceived cause of CKD rather than the CKD itself. A number of the included studies had recruitment periods that began before 2002, so their populations would be a mixture of patients managed under the old and new guidance. However, in spite of all these limitations, the results of our analyses are consistent with those of other studies.

Implications for clinical practice and future research

In populations with CKD stages 3 and 4, lipid-modifying drugs and glycaemic control drugs may improve renal function, and lipid-modifying drugs are associated with a reduction in cardiovascular events. Although antihypertensive drugs and sodium bicarbonate are presumed to reduce CKD progression,[8 11-13] we did not find a significant treatment effect. However, the paucity of data and high

For peer review only - http://bmjopen.bmj.com/site/about/guidelines.xhtml

heterogeneity does not allow strong recommendations for clinical practice. Therefore, future research is needed into the effects of drugs on the progression in CKD, in particular in studies that focus on CKD progression as a primary outcome.

Our finding of limited data is important given that CKD is a common progressive disease. The drugs we considered are commonly used, but studies have concentrated on cardiovascular outcomes and not CKD outcomes.

CONCLUSIONS

This review suggests that glycaemic control and lipid regulation may improve renal function in patients with moderate CKD. There was no evidence of either benefit or harm from antihypertensive drugs. However, the data were limited and heterogeneity was high for many of the analyses. Therefore, our findings are tentative.

ACKNOWLEDGEMENTS

We would like to thank all those who clarified our queries about their trials and/or provided additional data including Professor Nathaniel Hawkins, Professor Fan Fan Hou, Ms Jennifer Meessen, Professor Bruce Neal, Professor Valdo Perkovic, Dr Ruth Peters, Professor Scott D Solomon, Dr Di Xie and a number of anonymous others. We would also like to acknowledge all those who provided data and/or clarification about their trials which, unfortunately, we were unable to include in our study including Professor Fan Fan Hou and Dr Di Xie, Mr Colin Bicknell, Professor Bob Byington, Dr Enayet Karim Chowdhury, Professor Fan Fan Hou, Dr Dipak Kotecha, Dr Javier Martinez-Martin, Professor John McMurray, Dr Robert G Nelson, Nove Nordisk, Hiromi Rakugi MD, Dr Marcello Tonelli, Professor William White, Dr Di Xie and Professor Keng Thye Woo. Thanks also due to: Dr Boby Mihaylova who

For peer review only - http://bmjopen.bmj.com/site/about/guidelines.xhtml

For peer review only

helped produce the protocol for this review; Dr Richard Stevens, who provided some statistical advice; Dr Brian Shine, who provided advice on pooling different measures of CKD and proteinuria; Professor Paul Glasziou, Dr Boby Mihaylova, and from the Patient & Public Involvement Group, Dr Elizabeth Holloway, Dr Peter Kirby, and Mrs Rachael Patel, for their comments about the manuscript and helpful suggestions that have improved the text; and, the Stakeholder Group and Steering Committee of our NIHR Programme Grant, for discussions that inspired this paper.

COMPETING INTERESTS

Kathryn Taylor, Julie McLellan, Jeffrey Aronson and Susannah Fleming receive funding from the NIHR Programme for Applied Research.

Jan Verbakel receives funding from the NIHR Community Healthcare MIC, Nuffield Department of Primary Care Health Sciences, University of Oxford NIHR Diagnostic Evidence Co-operative (DEC). Daniel Lasserson receives funding from the NIHR Community Healthcare MIC.

FD Richard Hobbs acknowledges his part-funding from the National Institute for Health Research (NIHR) School for Primary Care Research, the NIHR Collaboration for Leadership in Health Research and Care (CLARHC) Oxford, the NIHR Oxford Biomedical Research Centre (BRC,)UHT), and the NIHR Oxford Medtech and In-Vitro Diagnostics Co-operative (MIC).

Rafael Perera receives funding from the NIHR Oxford Biomedical Research Centre Programme, the NIHR Programme for Applied Research, the NIHR HPRU Gastrointestinal Infections Group, and the NIHR Diagnostic Evidence Co-operative (DEC).

FUNDING

This article presents independent research funded by the NIHR under the Programme Grants for Applied Research programme (RP-PG-1210-12003). The views expressed are those of the authors and not necessarily those of the NHS, the NIHR or the Department of Health.

For peer review only - http://bmjopen.bmj.com/site/about/guidelines.xhtml

AUTHOR CONTRIBUTIONS

JM, RP, CB, JYV, JA, DSL, CoC, KST, RH and AB were involved in the conception and design of the study. NR, JM and JA devised the search strategy. NR completed the search. JM, NP, KST, JYV and SF selected studies for inclusion, and KST, JM, JYV and NP extracted data. KST carried out the data analysis with advice from RP. KST and JM together wrote the first draft of the manuscript and all authors contributed critically to subsequent revisions and approved the final manuscript. KST and JM had full access to all data in the study and take responsibility for the integrity and accuracy of the data analysis. KST and JM are guarantors.

ETHICAL APPROVAL

Not required.

DATA SHARING

No additional data are available.

TRANSPARENCY

The lead author (the manuscript's guarantor) affirms that the manuscript is an honest, accurate, and transparent account of the study being reported; that no important aspects of the study have been omitted; and that any discrepancies from the study as planned (and, if relevant, registered) have been explained.

For peer review only

FIGURE LEGENDS

Figure 1. PRISMA diagram

Figure 2. Ratio of means of estimated glomerular filtration rate at the end of the trials for antihypertensives vs comparator (boxes) and pooled estimates across studies (diamonds) calculated by the random effects DerSimonian and Laird method, split by chronic kidney disease stage

CKD – Chronic kidney disease; CHARMa and CHARMb – CKD stage 3 and 4 respectively with estimated glomerular filtration rate measured by the MDRD equation; IDNTa - Intervention is irbesartan; IDNTb - Intervention is amlodipine.

Figure 3. Ratio of means of estimated glomerular filtration rate at the end of the trials for lipid-modifying drugs vs comparator (boxes) and pooled estimates across studies (diamonds) calculated by the random effects DerSimonian and Laird method, split by chronic kidney disease stage

CKD – Chronic kidney disease; ASCOT-LLAa - Atorvastatin and amlodipine vs placebo and amlodipine; ASCOT-LLAb - Atorvastatin and atenolol vs placebo and atenolol; GISSI-HFa – CKD stage 3; GISSI-HFb-CKD stage 4; GREACEa – Atorvastatin vs usual care for the group with metabolic syndrome; GREACEb – Atorvastatin vs usual care for the group without metabolic syndrome; SHARPa – CKD stages 1 to 3; SHARPb – CKD stage 4.

Figure 4. Ratio of means estimated glomerular filtration rate at the end of the trials for glycaemic control drugs vs comparator (boxes) and pooled estimates across studies (diamonds) calculated by the random effects DerSimonian and Laird method, split by chronic kidney disease stage

CKD – Chronic kidney disease; Bode 2015L – Intervention is low dose canagliflozin (100mg per day); EMPA-REG H – Intervention is high dose empagliflozin (25 mg daily); EMPA-REG H – Intervention is low dose empagliflozin (10 mg daily); Kohan 2014L – Intervention is low dose dapagliflozin (5 mg per day); CANVASa – CKD stage 3a, CANVASb – CKD stage 3b; SHARPa – CKD stages 1 to 3; SHARPb – CKD stage 4; TECOSa – CKD stage 3a; TECOSb – CKD stage 3b.

For peer review only - http://bmjopen.bmj.com/site/about/guidelines.xhtml

For peer review only

Figure 5. Effect of treatment on estimated glomerular filtration rate by drug class: chronic kidney disease stage 3 and 4 only

ROM>1 favoured the intervention group (higher values are better). "Studies" refers to the study of different populations. Of the 35 included studies, 15 reported renal function of patients with CKD stage 3 and/or stage 4. Of these, 9 reported renal function of a single population and 6 reported results for two CKD subgroups or dose dependent subgroups.

Figure 6. Relative risk of cardiovascular disease during trials of lipid-modifying drugs vs comparator (boxes) and pooled estimates across trials (diamonds) calculated by the random effects DerSimonian and Laird method, split by chronic kidney disease stage.

GREACEa – Atorvastatin vs usual care for the group with metabolic syndrome; GREACEb – Atorvastatin vs usual care for the group without metabolic syndrome.

Figure 7. Relative risk of all-cause mortality during trials of lipid-modifying drugs vs comparator (boxes) and pooled estimates across trials (diamonds) calculated by the random effects DerSimonian and Laird method, split by chronic kidney disease stage.

GREACEa – Atorvastatin vs usual care for the group with metabolic syndrome; GREACEb – Atorvastatin vs usual care for the group without metabolic syndrome.

References

1. Hill NR, Fatoba ST, Oke JL, et al. Global prevalence of chronic kidney disease - a systematic review and meta-analysis. *PloS One* 2016; 11(7): e0158765. doi: 10.1371/journal.pone.0158765 [published Online First: 2016/07/08]

2. Go AS, Chertow GM, Fan D, et al. Chronic kidney disease and the risks of death, cardiovascular events, and hospitalization. *N Engl J Med* 2004; 351(13): 1296-305. doi: 10.1056/NEJMoa041031

For peer review only - http://bmjopen.bmj.com/site/about/guidelines.xhtml

3. National Institute for Health and Care Excellence. Chronic kidney disease: early identification and management of chronic kidney disease in adults in primary and secondary care (CG182). London: National Clinical Guideline Centre, 2014.

4. Levey AS, Eckardt KU, Tsukamoto Y, et al. Definition and classification of chronic kidney disease: a position statement from Kidney Disease: Improving Global Outcomes (KDIGO). *Kidney Int* 2005; 67(6): 2089-100. doi: 10.1111/j.1523-1755.2005.00365.x [published Online First: 2005/05/11]

5. Levin A, Stevens PE. Summary of KDIGO 2012 CKD Guideline: behind the scenes, need for guidance, and a framework for moving forward. *Kidney Int* 2014; 85(1): 49-61. doi: 10.1038/ki.2013.444 [published Online First: 2013/11/29]

6. de Lusignan S, Tomson C, Harris K, et al. UK Prevalence of Chronic Kidney Disease for the Adult Population Is 6.76% Based on Two Creatinine Readings. *Nephron Clin Pract* 2012; 120(2): c107-c07.

7. Oke J, Shine B, McFadden E, et al. Trends in serum creatinine testing in Oxfordshire, UK, 1993-2013: a population-based cohort study. *BMJ Open* 2015; 5(12): e009459. doi: 10.1136/bmjopen-2015-009459 [published Online First: 2015/12/18]

8. Peterson JC, Adler S, Burkart JM, et al. Blood pressure control, proteinuria, and the progression of renal disease. The Modification of Diet in Renal Disease Study. *Ann Intern Med* 1995; 123(10): 754-62.

9. Sandhu S, Wiebe N, Fried LF, et al. Statins for improving renal outcomes: a meta-analysis. *J Am Soc Nephrol* 2006; 17(7): 2006-16. doi: 10.1681/ASN.2006010012

10. Wang PH, Lau J, Chalmers TC. Meta-analysis of effects of intensive blood-glucose control on late complications of type I diabetes. *Lancet* 1993; 341(8856): 1306-9.

11. Phisitkul S, Khanna A, Simoni J, et al. Amelioration of metabolic acidosis in patients with low GFR reduced kidney endothelin production and kidney injury, and better preserved GFR. *Kidney Int* 2010; 77(7): 617-23. doi: 10.1038/ki.2009.519

For peer review only - http://bmjopen.bmj.com/site/about/guidelines.xhtml

12. Mahajan A, Simoni J, Sheather SJ, et al. Daily oral sodium bicarbonate preserves glomerular filtration rate by slowing its decline in early hypertensive nephropathy. *Kidney Int* 2010; 78(3): 303-9. doi: 10.1038/ki.2010.129

13. Brito-Ashurst Id, Varagunam M, Raftery MJ, et al. Bicarbonate supplementation slows progression of CKD and improves nutritional status. *J Am Soc Nephrol* 2009; 20(9): 2075-84.

14. Lv J, Ehteshami P, Sarnak MJ, et al. Effects of intensive blood pressure lowering on the progression of chronic kidney disease: a systematic review and meta-analysis. *CMAJ* 2013; 185(11): 949-57. doi: 10.1503/cmaj.121468

15. Bakris GL. Slowing nephropathy progression: focus on proteinuria reduction. *Clin J Am Soc Nephrol* 2008; 3 Suppl 1: S3-10. doi: 10.2215/CJN.03250807

16. Lewis A, Emberson JR, Blackwell LJ, et al. Effect of Lowering LDL-Cholesterol on Kidney Function: Meta-Analysis of Data from 120,000 Participants in 21 Randomized Trials. *J Am Soc Nephrol Abstract supplement* 2012; 23: 685A.

17. Liberati A, Altman DG, Tetzlaff J, et al. The PRISMA statement for reporting systematic reviews and meta-analyses of studies that evaluate healthcare interventions: explanation and elaboration. *BMJ* 2009; 339: b2700. doi: 10.1136/bmj.b2700

18. Levey AS, Bosch JP, Lewis JB, et al. A more accurate method to estimate glomerular filtration rate from serum creatinine: a new prediction equation. Modification of Diet in Renal Disease Study Group. *Ann Intern Med* 1999; 130(6): 461-70. [published Online First: 1999/03/13]

19. Neuen BL, Ohkuma T, Neal B, et al. Cardiovascular and Renal Outcomes With Canagliflozin According to Baseline Kidney Function: Data from the CANVAS Program. *Circulation* 2018 doi: 10.1161/circulationaha.118.035901 [published Online First: 2018/06/27]

20. Neal B, Perkovic V, Matthews DR. Canagliflozin and Cardiovascular and Renal Events in Type 2 Diabetes. *N Engl J Med* 2017; 377(21): 2099. doi: 10.1056/NEJMc1712572 [published Online First: 2017/11/23]

21. Higgins JPT, Green S. Cochrane Handbook for Systematic Reviews of Interventions: Wiley 2011.

For peer review only - http://bmjopen.bmj.com/site/about/guidelines.xhtml

22. Higgins JP. Commentary: Heterogeneity in meta-analysis should be expected and appropriately quantified. *Int J Epidemiol* 2008; 37(5): 1158-60. doi: 10.1093/ije/dyn204 [published Online First: 2008/10/04]

23. DerSimonian R, Laird N. Meta-analysis in clinical trials. *Control Clin Trials* 1986; 7(3): 177-88. [published Online First: 1986/09/01]

24. Riley RD, Higgins JP, Deeks JJ. Interpretation of random effects meta-analyses. *BMJ* 2011; 342: d549. doi: 10.1136/bmj.d549 [published Online First: 2011/02/12]

25. Higgins JP, Thompson SG, Spiegelhalter DJ. A re-evaluation of random-effects meta-analysis. *J R Stat Soc Ser A Stat Soc* 2009; 172(1): 137-59. doi: 10.1111/j.1467-985X.2008.00552.x [published Online First: 2009/04/22]

26. Friedrich JO, Adhikari NK, Beyene J. The ratio of means method as an alternative to mean differences for analyzing continuous outcome variables in meta-analysis: a simulation study. *BMC Med Res Methodol* 2008; 8: 32. doi: 10.1186/1471-2288-8-32

27. Friedrich JO, Adhikari NK, Beyene J. Ratio of means for analyzing continuous outcomes in meta-analysis performed as well as mean difference methods. *J Clin Epidemiol* 2011; 64(5): 556-64. doi: 10.1016/j.jclinepi.2010.09.016 [published Online First: 2011/03/31]

28. Guyatt GH, Thorlund K, Oxman AD, et al. GRADE guidelines: 13. Preparing summary of findings tables and evidence profiles-continuous outcomes. *J Clin Epidemiol* 2013; 66(2): 173-83. doi: 10.1016/j.jclinepi.2012.08.001 [published Online First: 2012/11/03]

29. Levey AS, Stevens LA, Schmid CH, et al. A new equation to estimate glomerular filtration rate. *Ann Intern Med* 2009; 150(9): 604-12. [published Online First: 2009/05/06]

30. Mantel N, Haenszel W. Statistical aspects of the analysis of data from retrospective studies of disease. *J Natl Cancer Inst* 1959; 22(4): 719-48. [published Online First: 1959/04/01]

31. Greenland S, Robins JM. Estimation of a common effect parameter from sparse follow-up data. *Biometrics* 1985; 41(1): 55-68. [published Online First: 1985/03/01]

For peer review only

For peer review only - http://bmjopen.bmj.com/site/about/guidelines.xhtml

32. Cornell JE, Mulrow CD, Localio R, et al. Random-effects meta-analysis of inconsistent effects: a time for change. *Ann Intern Med* 2014; 160(4): 267-70. doi: 10.7326/m13-2886 [published Online First: 2014/04/15]

33. Rover C, Knapp G, Friede T. Hartung-Knapp-Sidik-Jonkman approach and its modification for random-effects meta-analysis with few studies. *BMC Med Res Methodol* 2015; 15: 99. doi: 10.1186/s12874-015-0091-1 [published Online First: 2015/11/18]

34. Hartung J, Knapp G. A refined method for the meta-analysis of controlled clinical trials with binary outcome. *Stat Med* 2001; 20(24): 3875-89. [published Online First: 2002/01/10]

35. Peters JL, Sutton AJ, Jones DR, et al. Contour-enhanced meta-analysis funnel plots help distinguish publication bias from other causes of asymmetry. *J Clin Epidemiol* 2008; 61(10): 991-6. doi: 10.1016/j.jclinepi.2007.11.010 [published Online First: 2008/06/10]

36. Stata Statistical Software: Release 14 [program]. College Station, TX: StataCorp LP, 2015.

37. Review Manager (RevMan) [program]. Version 5.3. Copenhagen: The Nordic Cochrane Centre, 2014.

38. Hawkins NM, Jhund PS, Pozzi A, et al. Severity of renal impairment in patients with heart failure and atrial fibrillation: implications for non-vitamin K antagonist oral anticoagulant dose adjustment. *Eur J Heart Fail* 2016; 18(9): 1162-71. doi: 10.1002/ejhf.614 [published Online First: 2016/09/07]

39. Hillege HL, Nitsch D, Pfeffer MA, et al. Renal function as a predictor of outcome in a broad spectrum of patients with heart failure. *Circulation* 2006; 113(5): 671-8. doi: 10.1161/circulationaha.105.580506 [published Online First: 2006/02/08]

40. Pfeffer MA, Swedberg K, Granger CB, et al. Effects of candesartan on mortality and morbidity in patients with chronic heart failure: the CHARM-Overall programme. *Lancet* 2003; 362(9386): 759-66. [published Online First: 2003/09/19]

For peer review only

For peer review only - http://bmjopen.bmj.com/site/about/guidelines.xhtml

41. Leiter LA, Cefalu WT, de Bruin TW, et al. Long-term maintenance of efficacy of dapagliflozin in patients with type 2 diabetes mellitus and cardiovascular disease. *Diabetes Obes Metab* 2016; 18(8): 766-74. doi: 10.1111/dom.12666 [published Online First: 2016/03/25]

42. Cefalu WT, Leiter LA, de Bruin TW, et al. Dapagliflozin's Effects on Glycemia and Cardiovascular Risk Factors in High-Risk Patients With Type 2 Diabetes: A 24-Week, Multicenter, Randomized, Double-Blind, Placebo-Controlled Study With a 28-Week Extension. *Diabetes Care* 2015; 38(7): 1218-27. doi: 10.2337/dc14-0315 [published Online First: 2015/04/09]

43. Leiter LA, Cefalu WT, de Bruin TW, et al. Dapagliflozin added to usual care in individuals with type 2 diabetes mellitus with preexisting cardiovascular disease: a 24-week, multicenter, randomized, double-blind, placebo-controlled study with a 28-week extension. *J Am Geriatr Soc* 2014; 62(7): 1252-62. doi: 10.1111/jgs.12881 [published Online First: 2014/06/04]

44. Hou FF, Zhang X, Zhang GH, et al. Efficacy and safety of benazepril for advanced chronic renal insufficiency. *N Engl J Med* 2006; 354(2): 131-40. doi: 10.1056/NEJMoa053107 [published Online First: 2006/01/13]

45. Fassett RG, Robertson IK, Ball MJ, et al. Effect of atorvastatin on kidney function in chronic kidney disease: a randomised double-blind placebo-controlled trial. *Atherosclerosis* 2010; 213(1): 218-24.

46. Sakata Y, Shiba N, Takahashi J, et al. Clinical impacts of additive use of olmesartan in hypertensive patients with chronic heart failure: the supplemental benefit of an angiotensin receptor blocker in hypertensive patients with stable heart failure using olmesartan (SUPPORT) trial. *Eur Heart J* 2015; 36(15). http: //cochranelibrary-wiley.com/o/cochrane/clcentral/articles/437/CN-01255437/frame.html https: //www.ncbi.nlm.nih.gov/pmc/articles/PMC4466154/pdf/ehu504.pdf.

47. Sakata Y, Nochioka K, Miura M, et al. Supplemental benefit of an angiotensin receptor blocker in hypertensive patients with stable heart failure using olmesartan (SUPPORT) trial--rationale and

For peer review only - http://bmjopen.bmj.com/site/about/guidelines.xhtml

design. *J Cardiol* 2013; 62(1): 31-6. doi: 10.1016/j.jjcc.2013.02.011 [published Online First: 2013/06/19]

48. Kimura, Genjiro, Kasahara, et al. Effects of atorvastatin on renal function in patients with dyslipidemia and chronic kidney disease: assessment of clinical usefulness in CKD patients with atorvastatin (ASUCA) trial. *Clin Exp Nephrol* 2016; 8: 8.

49. Management of Elevated Cholesterol in the Primary Prevention Group of Adult Japanese (MEGA) Study Group. Design and baseline characteristics of a study of primary prevention of coronary events with pravastatin among Japanese with mildly elevated cholesterol levels. *Circ J* 2004; 68(9): 860-7. [published Online First: 2004/08/27]

50. Nakamura H, Mizuno K, Ohashi Y, et al. Pravastatin and cardiovascular risk in moderate chronic kidney disease. *Atherosclerosis* 2009; 206(2): 512-7.

51. Nakamura H, Arakawa K, Itakura H, et al. Primary prevention of cardiovascular disease with pravastatin in Japan (MEGA Study): a prospective randomised controlled trial. *Lancet* 2006; 368(9542): 1155-63. doi: 10.1016/s0140-6736(06)69472-5 [published Online First: 2006/10/03]

52. Torres VE, Abebe KZ, Chapman AB, et al. Angiotensin blockade in late autosomal dominant polycystic kidney disease. *N Engl J Med* 2014; 371(24): 2267-76. doi: 10.1056/NEJMoa1402686 [published Online First: 2014/11/18]

53. Lewis EJ, Hunsicker LG, Clarke WR, et al. Renoprotective effect of the angiotensin-receptor antagonist irbesartan in patients with nephropathy due to type 2 diabetes. *N Engl J Med* 2001; 345(12): 851-60. doi: 10.1056/NEJMoa011303 [published Online First: 2001/09/22]

54. Evans M, Bain SC, Hogan S, et al. Irbesartan delays progression of nephropathy as measured by estimated glomerular filtration rate: post hoc analysis of the Irbesartan Diabetic Nephropathy Trial. *Nephrol Dial Transplant* 2012; 27(6): 2255-63. doi: 10.1093/ndt/gfr696 [published Online First: 2011/12/17]

For peer review only

For peer review only - http://bmjopen.bmj.com/site/about/guidelines.xhtml

55. Berl T, Hunsicker LG, Lewis JB, et al. Cardiovascular outcomes in the Irbesartan Diabetic Nephropathy Trial of patients with type 2 diabetes and overt nephropathy. *Ann Intern Med* 2003; 138(7): 542-9. [published Online First: 2003/04/02]

56. Lambers Heerspink HJ, Weldegiorgis M, Inker LA, et al. Estimated GFR decline as a surrogate end point for kidney failure: a post hoc analysis from the Reduction of End Points in Non-Insulin-Dependent Diabetes With the Angiotensin II Antagonist Losartan (RENAAL) study and Irbesartan Diabetic Nephropathy Trial (IDNT). *Am J Kidney Dis* 2014; 63(2): 244-50. doi: 10.1053/j.ajkd.2013.09.016 [published Online First: 2013/11/12]

57. Pfeffer MA, Braunwald E, Moye LA, et al. Effect of captopril on mortality and morbidity in patients with left ventricular dysfunction after myocardial infarction. Results of the survival and ventricular enlargement trial. The SAVE Investigators. *N Engl J Med* 1992; 327(10): 669-77. doi: 10.1056/nejm199209033271001 [published Online First: 1992/09/13]

58. Downs JR, Clearfield M, Weis S, et al. Primary prevention of acute coronary events with lovastatin in men and women with average cholesterol levels: results of AFCAPS/TexCAPS. Air Force/Texas Coronary Atherosclerosis Prevention Study. *JAMA* 1998; 279(20): 1615-22. [published Online First: 1998/06/05]

59. Kendrick J, Shlipak MG, Targher G, et al. Effect of lovastatin on primary prevention of cardiovascular events in mild CKD and kidney function loss: a post hoc analysis of the Air Force/Texas Coronary Atherosclerosis Prevention Study. *Am J Kidney Dis* 2010; 55(1): 42-9.

60. Downs JR, Beere PA, Whitney E, et al. Design & rationale of the Air Force/Texas Coronary Atherosclerosis Prevention Study (AFCAPS/TexCAPS). *Am J Cardiol* 1997; 80(3): 287-93. [published Online First: 1997/08/01]

61. Robins SJ, Collins D, Wittes JT, et al. Relation of gemfibrozil treatment and lipid levels with major coronary events: VA-HIT: a randomized controlled trial. *JAMA* 2001; 285(12): 1585-91. [published Online First: 2001/04/13]

For peer review only - http://bmjopen.bmj.com/site/about/guidelines.xhtml

62. Tonelli M, Collins D, Robins S, et al. Effect of gemfibrozil on change in renal function in men with moderate chronic renal insufficiency and coronary disease. *Am J Kidney Dis* 2004; 44(5): 832-9.

63. Rubins HB, Robins SJ, Iwane MK, et al. Rationale and design of the Department of Veterans Affairs High-Density Lipoprotein Cholesterol Intervention Trial (HIT) for secondary prevention of coronary artery disease in men with low high-density lipoprotein cholesterol and desirable low-density lipoprotein cholesterol. *Am J Cardiol* 1993; 71(1): 45-52. [published Online First: 1993/01/01]

64. Goraya N, Munoz-Maldonado Y, Simoni J, et al. Treatment of metabolic acidosis in chronic kidney disease with fruits and vegetables but not sodium bicarbonate yields fewer adverse cardiovascular events after five-years follow up. *Circulation Conference: Resuscitation Science Symposium, ReSS* 2017; 136(Supplement 1)

65. Goraya N, Simoni J, Jo CH, et al. Treatment of metabolic acidosis in patients with stage 3 chronic kidney disease with fruits and vegetables or oral bicarbonate reduces urine angiotensinogen and preserves glomerular filtration rate. *Kidney Int* 2014; 86(5): 1031-8.

66. Bianchi S, Bigazzi R, Campese VM. Intensive versus conventional therapy to slow the progression of idiopathic glomerular diseases. *Am J Kidney Dis* 2010; 55(4): 671-81. doi: 10.1053/j.ajkd.2009.11.006 [published Online First: 2010/01/26]

67. Asselbergs FW, Diercks GF, Hillege HL, et al. Effects of fosinopril and pravastatin on cardiovascular events in subjects with microalbuminuria. *Circulation* 2004; 110(18): 2809-16. doi: 10.1161/01.cir.0000146378.65439.7a [published Online First: 2004/10/20]

68. Huskey J, Lindenfeld J, Cook T, et al. Effect of simvastatin on kidney function loss in patients with coronary heart disease: findings from the Scandinavian Simvastatin Survival Study (4S). *Atherosclerosis* 2009; 205(1): 202-6.

69. Pedersen TR, Wilhelmsen L, Faergeman O, et al. Follow-up study of patients randomized in the Scandinavian simvastatin survival study (4S) of cholesterol lowering. *Am J Cardiol* 2000; 86(3): 257-62. [published Online First: 2000/08/03]

For peer review only - http://bmjopen.bmj.com/site/about/guidelines.xhtml

70. Design and baseline results of the Scandinavian Simvastatin Survival Study of patients with stable angina and/or previous myocardial infarction. *The Am J Cardiol* 1993; 71(5): 393-400. [published Online First: 1993/02/15]

71. Sever PS, Dahlof B, Poulter NR, et al. Prevention of coronary and stroke events with atorvastatin in hypertensive patients who have average or lower-than-average cholesterol concentrations, in the Anglo-Scandinavian Cardiac Outcomes Trial--Lipid Lowering Arm (ASCOT-LLA): a multicentre randomised controlled trial. *Lancet* 2003; 361(9364): 1149-58. doi: 10.1016/s0140-6736(03)12948-0 [published Online First: 2003/04/11]

72. Gupta A, Chang CL, Collier D, et al. The relationship between statin therapy and progression of renal damage among 10305 hypertensive patients randomised in the ascot-Lipid-Lowering Arm (LLA). *Atherosclerosis Supplements* 2011; Conference: 79th European Atherosclerosis Society Congress, EAS 2011 Gothenburg Sweden. Conference Start: 20110626 Conference End: 20110629. Conference Publication: (var.pagings). 12 (1): 158-59.

73. Nanayakkara PWB, Guldener Cv, Wee PMt, et al. Effect of a treatment strategy consisting of pravastatin, vitamin E, and homocysteine lowering on carotid intima-media thickness, endothelial function, and renal function in patients with mild to moderate chronic kidney disease: results from the Anti-Oxidant Therapy in Chronic Renal Insufficiency (ATIC) Study. *Arch Intern Med* 2007; 167(12): 1262-70.

74. Tavazzi L, Maggioni AP, Marchioli R, et al. Effect of rosuvastatin in patients with chronic heart failure (the GISSI-HF trial): a randomised, double-blind, placebo-controlled trial. *Lancet* 2008; 372(9645): 1231-9. doi: 10.1016/s0140-6736(08)61240-4 [published Online First: 2008/09/02]

75. Tavazzi L, Tognoni G, Franzosi MG, et al. Rationale and design of the GISSI heart failure trial: a large trial to assess the effects of n-3 polyunsaturated fatty acids and rosuvastatin in symptomatic congestive heart failure. *Eur J Heart Fail* 2004; 6(5): 635-41. doi: 10.1016/j.ejheart.2004.03.001 [published Online First: 2004/08/11]

For peer review only - http://bmjopen.bmj.com/site/about/guidelines.xhtml

76. Athyros VG, Mikhailidis DP, Liberopoulos EN, et al. Effect of statin treatment on renal function and serum uric acid levels and their relation to vascular events in patients with coronary heart disease and metabolic syndrome. *Nephrol Dial Transplant* 2007; 22(1): 118-27.

77. Athyros VG, Papageorgiou AA, Mercouris BR, et al. Treatment with atorvastatin to the National Cholesterol Educational Program goal versus 'usual' care in secondary coronary heart disease prevention. The GREek Atorvastatin and Coronary-heart-disease Evaluation (GREACE) study. *Curr Med Res Opin* 2002; 18(4): 220-8. [published Online First: 2002/08/31]

78. Ford I, Bezlyak V, Stott DJ, et al. Reduced glomerular filtration rate and its association with clinical outcome in older patients at risk of vascular events: secondary analysis. *PLoS Med* 2009; 6 (1)(e16)

79. Patel A, MacMahon S, Chalmers J, et al. Effects of a fixed combination of perindopril and indapamide on macrovascular and microvascular outcomes in patients with type 2 diabetes mellitus (the ADVANCE trial): a randomised controlled trial. *Lancet* 2007; 370(9590): 829-40. doi: 10.1016/s0140-6736(07)61303-8 [published Online First: 2007/09/04]

80. Heerspink HJ, Ninomiya T, Perkovic V, et al. Effects of a fixed combination of perindopril and indapamide in patients with type 2 diabetes and chronic kidney disease. *Eur Heart J* 2010; 31(23): 2888-96. doi: 10.1093/eurheartj/ehq139 [published Online First: 2010/05/27]

81. Peters R, Beckett N, Poulter R, et al. Kidney function in the very elderly with hypertension: data from the hypertension in the very elderly (HYVET) trial. *Age Ageing* 2013; 42(2): 253-8. doi: 10.1093/ageing/afs109 [published Online First: 2012/08/23]

82. Beckett NS, Peters R, Fletcher AE, et al. Treatment of hypertension in patients 80 years of age or older. *N Engl J Med* 2008; 358(18): 1887-98. doi: 10.1056/NEJMoa0801369 [published Online First: 2008/04/02]

83. Bulpitt C, Fletcher A, Beckett N, et al. Hypertension in the Very Elderly Trial (HYVET): protocol for the main trial. *Drugs Aging* 2001; 18(3): 151-64. [published Online First: 2001/04/17]

For peer review only - http://bmjopen.bmj.com/site/about/guidelines.xhtml

84. Ting RD, Keech AC, Drury PL, et al. Benefits and safety of long-term fenofibrate therapy in people with type 2 diabetes and renal impairment: the FIELD Study. *Diabetes Care* 2012; 35(2): 218-25.

85. The need for a large-scale trial of fibrate therapy in diabetes: the rationale and design of the Fenofibrate Intervention and Event Lowering in Diabetes (FIELD) study. [ISRCTN64783481]. *Cardiovasc Diabetol* 2004; 3: 9. doi: 10.1186/1475-2840-3-9 [published Online First: 2004/12/02]

86. Keech A, Simes RJ, Barter P, et al. Effects of long-term fenofibrate therapy on cardiovascular events in 9795 people with type 2 diabetes mellitus (the FIELD study): randomised controlled trial. *Lancet* 2005; 366(9500): 1849-61. doi: 10.1016/s0140-6736(05)67667-2 [published Online First: 2005/11/29]

87. Zinman B, Wanner C, Lachin JM, et al. Empagliflozin, Cardiovascular Outcomes, and Mortality in Type 2 Diabetes. *N Engl J Med* 2015; 373(22): 2117-28. doi: 10.1056/NEJMoa1504720 [published Online First: 2015/09/18]

88. Wanner C, Inzucchi SE, Lachin JM, et al. Empagliflozin and Progression of Kidney Disease in Type 2 Diabetes. *N Engl J Med* 2016; 375(4): 323-34. doi: 10.1056/NEJMoa1515920 [published Online First: 2016/06/15]

89. Cornel JH, Bakris GL, Stevens SR, et al. Effect of Sitagliptin on Kidney Function and Respective Cardiovascular Outcomes in Type 2 Diabetes: Outcomes From TECOS. *Diabetes Care* 2016; 39(12): 2304-10. doi: 10.2337/dc16-1415 [published Online First: 2016/10/16]

90. Green JB, Bethel MA, Armstrong PW, et al. Effect of Sitagliptin on Cardiovascular Outcomes in Type 2 Diabetes. *N Engl J Med* 2015; 373(3): 232-42. doi: 10.1056/NEJMoa1501352 [published Online First: 2015/06/09]

91. Zannad F, McMurray JJ, Krum H, et al. Eplerenone in patients with systolic heart failure and mild symptoms. *N Engl J Med* 2011; 364(1): 11-21. doi: 10.1056/NEJMoa1009492 [published Online First: 2010/11/16]

92. Rossignol P, Dobre D, McMurray JJ, et al. Incidence, determinants, and prognostic significance of hyperkalemia and worsening renal function in patients with heart failure receiving the

For peer review only

For peer review only - http://bmjopen.bmj.com/site/about/guidelines.xhtml

mineralocorticoid receptor antagonist eplerenone or placebo in addition to optimal medical therapy: results from the Eplerenone in Mild Patients Hospitalization and Survival Study in Heart Failure (EMPHASIS-HF). *Circ Heart Fail* 2014; 7(1): 51-8. doi: 10.1161/circheartfailure.113.000792 [published Online First: 2013/12/04]

93. Brenner BM, Cooper ME, de Zeeuw D, et al. Effects of losartan on renal and cardiovascular outcomes in patients with type 2 diabetes and nephropathy. *N Engl J Med* 2001; 345(12): 861-9. doi: 10.1056/NEJMoa011161 [published Online First: 2001/09/22]

94. de Zeeuw D, Remuzzi G, Parving HH, et al. Proteinuria, a target for renoprotection in patients with type 2 diabetic nephropathy: lessons from RENAAL. *Kidney Int* 2004; 65(6): 2309-20. doi: 10.1111/j.1523-1755.2004.00653.x [published Online First: 2004/05/20]

95. Kowey PR, Dickson TZ, Zhang Z, et al. Losartan and end-organ protection--lessons from the RENAAL study. *Clin Cardiol* 2005; 28(3): 136-42. [published Online First: 2005/04/09]

96. Winkelmayer WC, Zhang Z, Shahinfar S, et al. Efficacy and safety of angiotensin II receptor blockade in elderly patients with diabetes. *Diabetes Care* 2006; 29(10): 2210-7. doi: 10.2337/dc06-0570 [published Online First: 2006/09/28]

97. Brenner BM, Cooper ME, de Zeeuw D, et al. The losartan renal protection study--rationale, study design and baseline characteristics of RENAAL (Reduction of Endpoints in NIDDM with the Angiotensin II Antagonist Losartan). *J Renin Angiotensin Aldosterone Syst* 2000; 1(4): 328-35. doi: 10.3317/jraas.2000.062 [published Online First: 2002/04/23]

98. Pitt B, Pfeffer MA, Assmann SF, et al. Spironolactone for heart failure with preserved ejection fraction. *N Engl J Med* 2014; 370(15): 1383-92. doi: 10.1056/NEJMoa1313731 [published Online First: 2014/04/11]

99. Crouse JR, 3rd, Raichlen JS, Riley WA, et al. Effect of rosuvastatin on progression of carotid intima-media thickness in low-risk individuals with subclinical atherosclerosis: the METEOR Trial. *JAMA* 2007; 297(12): 1344-53. doi: 10.1001/jama.297.12.1344 [published Online First: 2007/03/27]

For peer review only - http://bmjopen.bmj.com/site/about/guidelines.xhtml

100. Shepherd J, Blauw GJ, Murphy MB, et al. The design of a prospective study of Pravastatin in the Elderly at Risk (PROSPER). PROSPER Study Group. PROspective Study of Pravastatin in the Elderly at Risk. *Am J Cardiol* 1999; 84(10): 1192-7. [published Online First: 1999/11/24]

101. Shepherd J, Blauw GJ, Murphy MB, et al. Pravastatin in elderly individuals at risk of vascular disease (PROSPER): a randomised controlled trial. *Lancet* 2002; 360(9346): 1623-30. [published Online First: 2002/11/30]

102. Baigent C, Landray MJ, Reith C, et al. The effects of lowering LDL cholesterol with simvastatin plus ezetimibe in patients with chronic kidney disease (Study of Heart and Renal Protection): a randomised placebo-controlled trial. *Lancet* 2011; 377(9784): 2181-92.

103. Haynes R, Lewis D, Emberson J, et al. Effects of lowering LDL cholesterol on progression of kidney disease. *J Am Soc Nephrol* 2014; 25(8): 1825-33.

104. Sharp Collaborative G. Study of Heart and Renal Protection (SHARP): randomized trial to assess the effects of lowering low-density lipoprotein cholesterol among 9,438 patients with chronic kidney disease. *Am Heart J* 2010; 160(5): 785-94.e10. doi: 10.1016/j.ahj.2010.08.012 [published Online First: 2010/11/26]

105. Bode B, Stenlof K, Harris S, et al. Long-term efficacy and safety of canagliflozin over 104 weeks in patients aged 55-80 years with type 2 diabetes. *Diabetes Obes Metab* 2015; 17(3): 294-303. doi: 10.1111/dom.12428 [published Online First: 2014/12/17]

106. Bode B, Stenlof K, Sullivan D, et al. Efficacy and safety of canagliflozin treatment in older subjects with type 2 diabetes mellitus: a randomized trial. *Hosp Pract (1995)* 2013; 41(2): 72-84. doi: 10.3810/hp.2013.04.1020 [published Online First: 2013/05/18]

107. Kohan DE, Fioretto P, Tang W, et al. Long-term study of patients with type 2 diabetes and moderate renal impairment shows that dapagliflozin reduces weight and blood pressure but does not improve glycemic control. *Kidney Int* 2014; 85(4): 962-71. doi: 10.1038/ki.2013.356 [published Online First: 2013/09/27]

For peer review only

For peer review only - http://bmjopen.bmj.com/site/about/guidelines.xhtml

108. de Brito-Ashurst I, Varagunam M, Raftery MJ, et al. Bicarbonate supplementation slows progression of CKD and improves nutritional status. *J Am Soc Nephrol* 2009; 20(9): 2075-84. doi: 10.1681/ASN.2008111205

109. Palmer SC, Navaneethan SD, Craig JC, et al. HMG CoA reductase inhibitors (statins) for people with chronic kidney disease not requiring dialysis. *Cochrane Database Syst Rev* 2014(5): Cd007784. doi: 10.1002/14651858.CD007784.pub2 [published Online First: 2014/06/01]

110. Zhang X, Xiang C, Zhou YH, et al. Effect of statins on cardiovascular events in patients with mild to moderate chronic kidney disease: a systematic review and meta-analysis of randomized clinical trials. *BMC Cardiovasc Disord* 2014; 14: 19. doi: 10.1186/1471-2261-14-19 [published Online First: 2014/02/18]

111. Herrington WG, Emberson J, Mihaylova B, et al. Impact of renal function on the effects of LDL cholesterol lowering with statin-based regimens: a meta-analysis of individual participant data from 28 randomised trials. *Lancet Diabetes Endocrinol* 2016; 4(10): 829-39. doi: 10.1016/s2213-8587(16)30156-5 [published Online First: 2016/08/02]

For peer review only

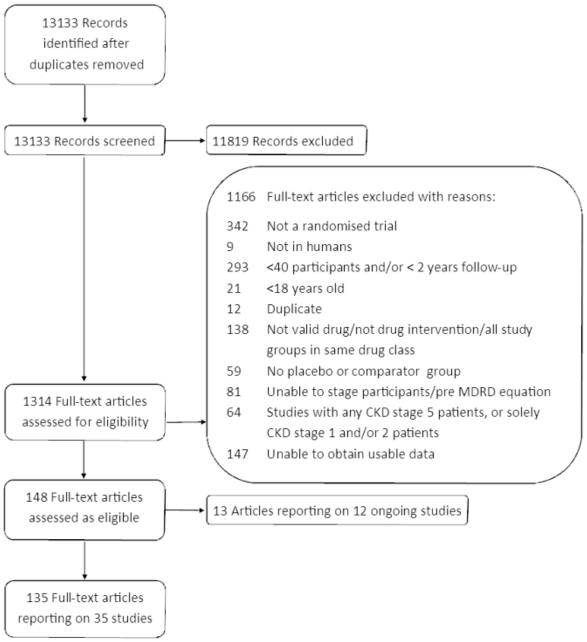

PRISMA diagram

104x148mm (300 x 300 DPI)

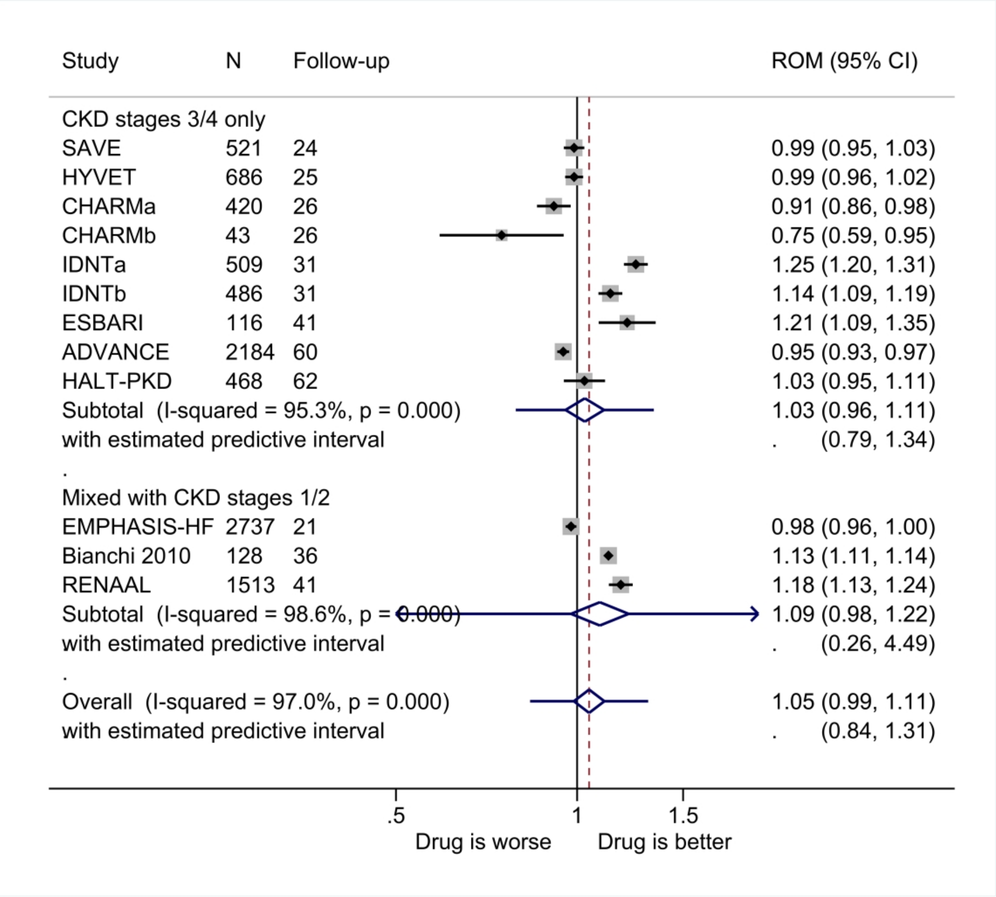

Ratio of means of estimated glomerular filtration rate at the end of the trials for antihypertensives vs comparator (boxes) and pooled estimates across studies (diamonds) calculated by the random effects DerSimonian and Laird method, split by chronic kidney disease stage
CKD – Chronic kidney disease; CHARMa and CHARMb – CKD stage 3 and 4 respectively with estimated glomerular filtration rate measured by the MDRD equation; IDNTa - Intervention is irbesartan; IDNTb - Intervention is amlodipine.

283x254mm (300 x 300 DPI)

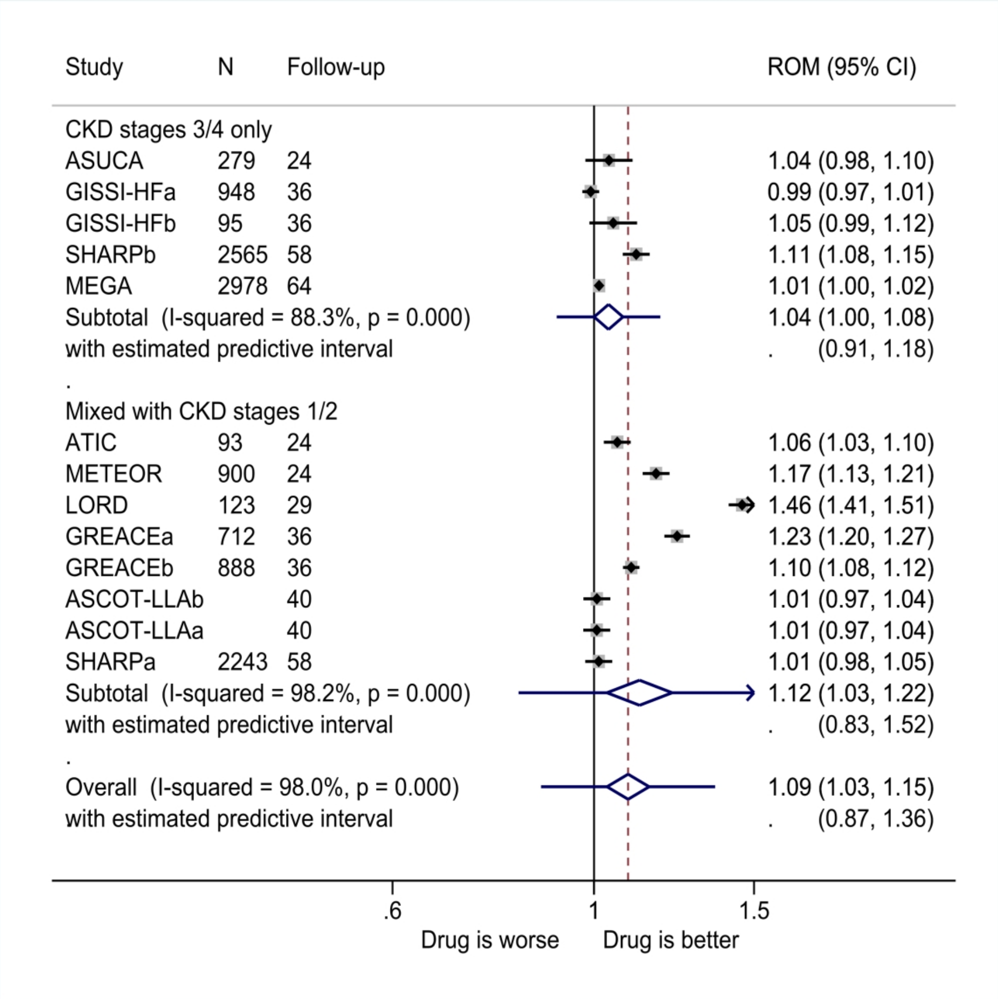

Ratio of means of estimated glomerular filtration rate at the end of the trials for lipid-modifying drugs vs comparator (boxes) and pooled estimates across studies (diamonds) calculated by the random effects DerSimonian and Laird method, split by chronic kidney disease stage
CKD – Chronic kidney disease; ASCOT-LLAa - Atorvastatin and amlodipine vs placebo and amlodipine; ASCOT-LLAb - Atorvastatin and atenolol vs placebo and atenolol; GISSI-HFa – CKD stage 3; GISSI-HFb- CKD stage 4; GREACEa – Atorvastatin vs usual care for the group with metabolic syndrome; GREACEb – Atorvastatin vs usual care for the group without metabolic syndrome; SHARPa – CKD stages 1 to 3; SHARPb – CKD stage 4.

126x126mm (600 x 600 DPI)

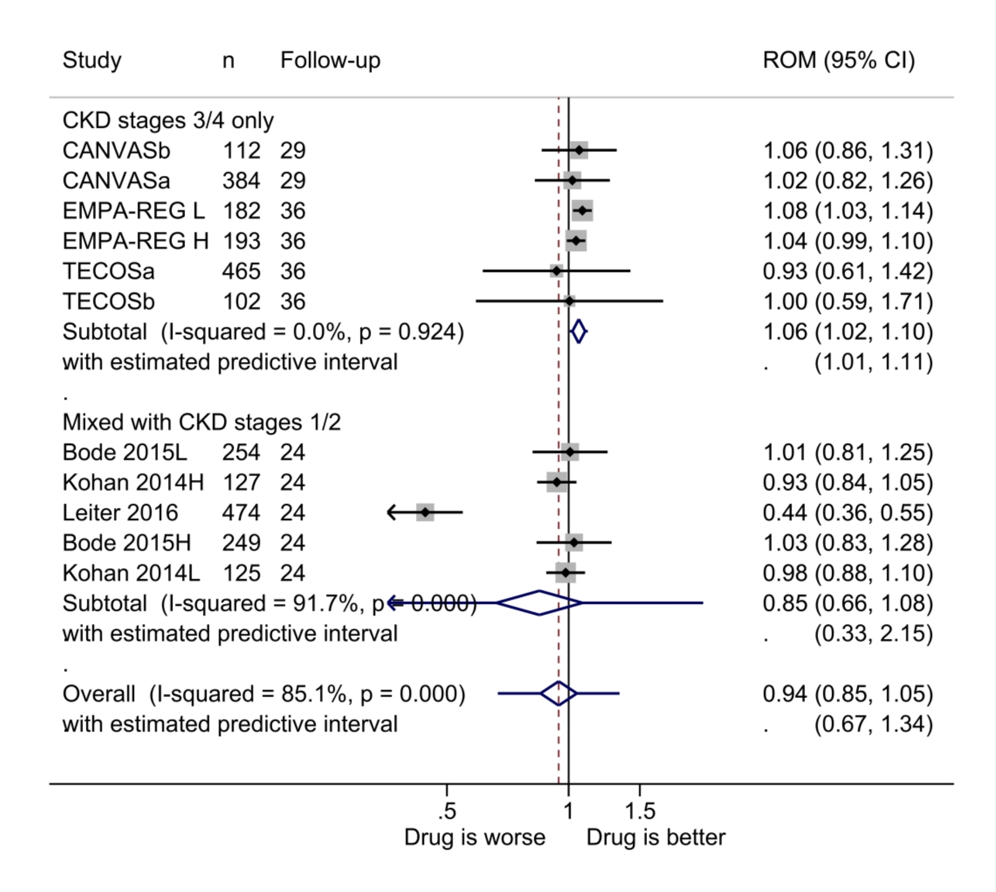

Ratio of means estimated glomerular filtration rate at the end of the trials for glycaemic control drugs vs comparator (boxes) and pooled estimates across studies (diamonds) calculated by the random effects DerSimonian and Laird method, split by chronic kidney disease stage
CKD – Chronic kidney disease; Bode 2015L – Intervention is low dose canagliflozin (100mg per day); EMPA-REG H – Intervention is high dose empagliflozin (25 mg daily); EMPA-REG H – Intervention is low dose empagliflozin (10 mg daily); Kohan 2014L – Intervention is low dose dapagliflozin (5 mg per day); CANVASa – CKD stage 3a, CANVASb – CKD stage 3b; SHARPa – CKD stages 1 to 3; SHARPb – CKD stage 4; TECOSa – CKD stage 3a; TECOSb – CKD stage 3b.

283x253mm (300 x 300 DPI)

Drug class	Studies	Population	I^2	Summary effect (95% confidence interval)
Antihypertensive	9	5433	95.3%	1.03 (0.96, 1.11)
Lipid modification	5	6865	88.3%	1.04 (1.00, 1.08)
Glycaemic control	6	1438	0%	1.06 (1.02, 1.10)
Bicarbonates	2	180	91.1%	1.08 (0.84, 1.38)

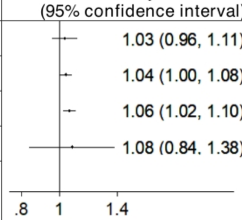

Effect of treatment on estimated glomerular filtration rate by drug class: chronic kidney disease stage 3 and 4 only
ROM>1 favoured the intervention group (higher values are better). "Studies" refers to the study of different populations. Of the 35 included studies, 15 reported renal function of patients with CKD stage 3 and/or stage 4. Of these, 9 reported renal function of a single population and 6 reported results for two CKD subgroups or dose dependent subgroups.

209x296mm (300 x 300 DPI)

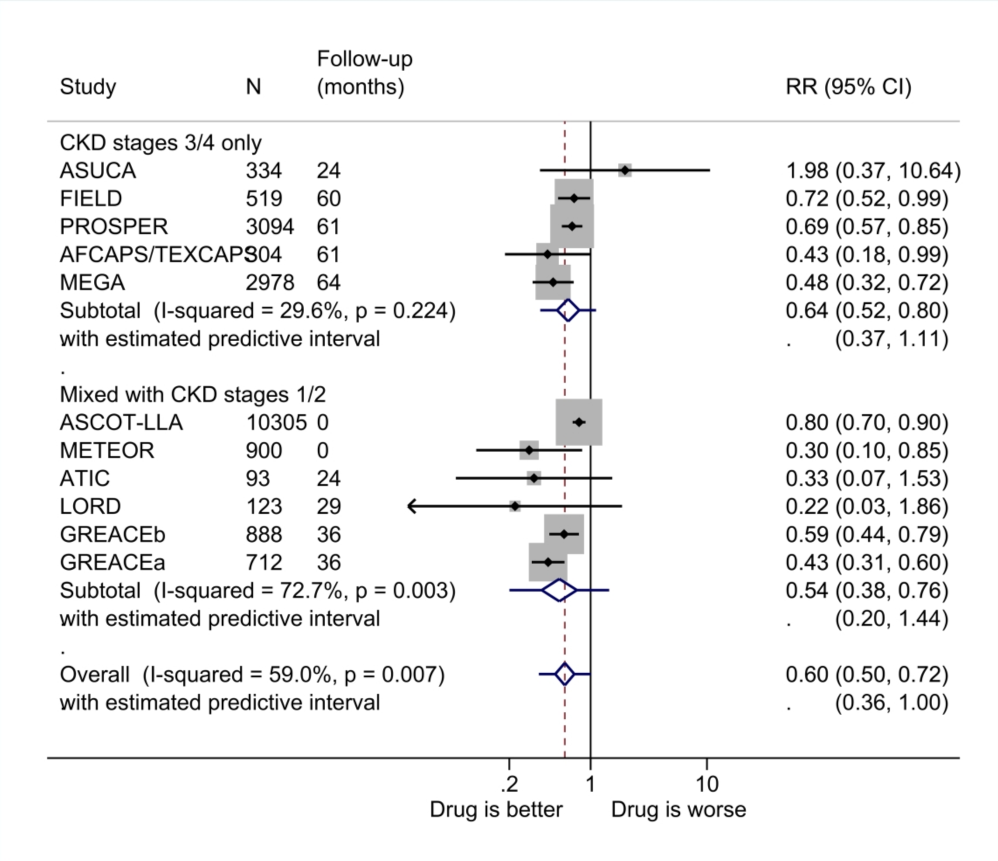

Relative risk of cardiovascular disease during trials of lipid-modifying drugs vs comparator (boxes) and pooled estimates across trials (diamonds) calculated by the random effects DerSimonian and Laird method, split by chronic kidney disease stage.
GREACEa – Atorvastatin vs usual care for the group with metabolic syndrome; GREACEb – Atorvastatin vs usual care for the group without metabolic syndrome.

281x242mm (300 x 300 DPI)

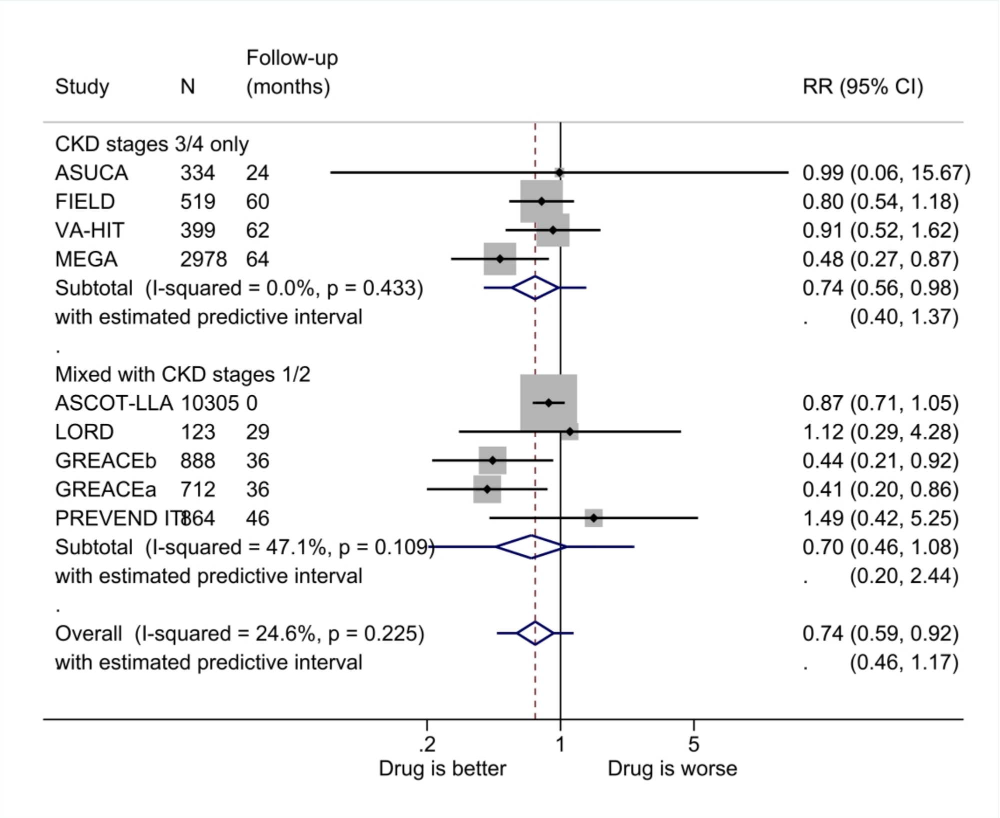

Relative risk of all-cause mortality during trials of lipid-modifying drugs vs comparator (boxes) and pooled estimates across trials (diamonds) calculated by the random effects DerSimonian and Laird method, split by chronic kidney disease stage.
GREACEa – Atorvastatin vs usual care for the group with metabolic syndrome; GREACEb – Atorvastatin vs usual care for the group without metabolic syndrome.

279x228mm (300 x 300 DPI)

SUPPLEMENTARY MATERIAL

Effects of antihypertensives, lipid-modifying drugs, glycaemic control drugs and sodium bicarbonate on the progression of stages 3 and 4 chronic kidney disease in adults: a systematic review and meta-analysis

Authors: Kathryn S Taylor, Julie McLellan, Jan Y Verbakel, Jeffrey Aronson, Daniel S Lasserson, Nicola Pidduck, Nia Roberts, Susannah Fleming, Christopher A O'Callaghan, Clare Bankhead, Amitava Banerjee, F.D. Richard Hobbs, Rafael Perera.

Corresponding author: Julie McLellan, Nuffield Department of Primary Care Health Sciences, University of Oxford, OX2 6GG, United Kingdom, julie.mclellan@phc.ox.ac.uk, 01865 289667

For peer review only

Contents

Search Strategy - MEDLINE Search Terms. 3
Supplementary Tables 7
Table 1. Kidney Disease Outcomes Quality Initiative classification (Levy 2003) 7
Table 2. Included drug list 8
Table 3. Characteristics of studies 12
Table 4. Interventions of studies 16
Table 5. Baseline characteristics 19
Table 6. Results of analyses 21
Table 7. GRADE assessments of primary outcome for each drug group 25
Supplementary Figures 27
Figure 1. Kidney Disease Improving Global outcomes (KDIGO) 2012 27
Figure 2. Cochrane Collaboration's Risk of bias 28
Figure 3. Funnel plots and contour enhanced funnel plots, for estimated glomerular filtration rate data, split by drug group 29
Figure 4. Ratio of means of estimated glomerular filtration rate at the end of the trials for anti-hypertensives vs comparator (boxes) and pooled estimates across studies (diamonds) calculated by the random effects DerSimonian and Laird method, for chronic kidney disease stages 3 and 4 and split by drug class 30
Figure 5. Ratio of means of proteinuria at the end of the trials for anti-hypertensives vs comparator (boxes) and pooled estimates across studies (diamonds) calculated by the random effects DerSimonian and Laird method, split by chronic kidney disease stage 31

For peer review only

For peer review only - http://bmjopen.bmj.com/site/about/guidelines.xhtml

Search Strategy - MEDLINE Search Terms.

# ▲	Searches
1	renal insufficiency/ or renal insufficiency, chronic/ or exp kidney failure, chronic/
2	(chronic adj (renal or kidney*) adj2 (fail* or insufficien* or disease? or disorder? or condition?)).ti,ab.
3	(late stage adj5 (kidney* or renal)).ti,ab.
4	((stage 3 or stage 3a or stage 3b or stage iii or stage iiia or stage iiib or stage 4 or stage iv) adj5 (kidney or renal)).ti,ab.
5	1 or 2 or 3 or 4
6	exp Angiotensin-Converting Enzyme Inhibitors/
7	((angiotensin adj2 converting enzyme inhibitor?) or ace inhibitor?).ti,ab.
8	(captopril or cilazapril or enalapril or fosinopril or imidapril or lisinopril or moexipril or perindopril or quinapril or ramipril or trandolapril).ti,ab.
9	exp Angiotensin Receptor Antagonists/
10	((angiotensin adj2 receptor antagonist?) or (angiotensin adj2 receptor blocker?) or sartans).ti,ab.
11	(ace or ace-i or ace-ii or acei or aceii or arb).ti,ab.
12	(azilsartan or candesartan or eprosartan or irbesartan or losartan or olmesartan or telmisartan or valsartan).ti,ab.
13	exp Thiazides/
14	thiazide*.ti,ab.
15	(Bendroflumethiazide or Chlortalidone or chlorthalidone or Cyclopenthiazide or Indapamide or Metolazone or Xipamide or Chlorothiazide or Hydrochlorothiazide).ti,ab.
16	exp Mineralocorticoid Receptor Antagonists/
17	aldosterone antagonist?.ti,ab.
18	(eplerenone or spironolactone).ti,ab.
19	exp Adrenergic beta-Antagonists/
20	(beta* blocker? or b-blocker? or beta* adrenoceptor antagonist? or b-adrenoceptor antagonist? or beta* adrenoceptor block* or b-adrenoceptor block* or beta* adrenergic block* or b adrenergic block*).ti,ab.
21	(propranolol or acebutolol or atenolol or bisoprolol or carvedilol or celiprolol or esmolol or labetalol or metropolol or nadolol or nebivolol or oxprenolol or pindolol or sotalol or timolol).ti,ab.
22	exp Calcium Channel Blockers/
23	(calcium channel block* or calcium antagonist?).ti,ab.
24	(amlodipine or diltiazem or felodipine or isradipine or lacidipine or lercanidipine or nicardipine or nifedipine or verapamil or nimodipine or nisoldipine).ti,ab.
25	exp Adrenergic alpha-Antagonists/
26	(alpha* blocker? or a-blocker? or alpha* adrenoceptor antagonist? or a-adrenoceptor antagonist? or alpha* adrenoceptor block* or a-adrenoceptor block* or postsynaptic alpha* block* or postsynaptic a-block*).ti,ab.
27	(doxazosin or indoramin or prazosin or terazosin).ti,ab.
28	Imidazoline Receptors/ag [Agonists]
29	(imidazoline receptor agonist? or centrally acting hypertensive*).ti,ab.

For peer review only - http://bmjopen.bmj.com/site/about/guidelines.xhtml

30	moxonidine.ti,ab.
31	(clonidine or methyldopa).ti,ab.
32	exp Vasodilator Agents/
33	vasodilator?.ti,ab.
34	(hydralazine or minoxidil).ti,ab.
35	Antihypertensive Agents/
36	(antihypertensive? or anti-hypertensive? or ((antihypertensive or anti-hypertensive) adj3 (drug? or agent? or therap* or treatment))).ti.
37	((blood pressure or bp) adj3 (drug? or agent? or therap* or treatment)).ti.
38	exp Insulins/
39	insulin?.ti,ab.
40	exp Sulfonylurea Compounds/
41	sulfonylurea?.ti,ab.
42	(Glibenclamide or gliclazide or glimepiride or glipizide or tolbutamide or chlorpropamide or glibornuride).ti,ab.
43	biguanides/ or metformin/
44	(biguanide? or metformin).ti,ab.
45	exp Glycoside Hydrolase Inhibitors/
46	(alpha glucosidase inhibitor? or acarbose).ti,ab.
47	Dipeptidyl-Peptidase IV Inhibitors/
48	(Dipeptidyl-Peptidase IV Inhibitor? or Dipeptidyl-Peptidase 4 Inhibitor? or DipeptidylPeptidase IV Inhibitor? or DipeptidylPeptidase 4 Inhibitor? or Dipeptidyl-Peptidase type IV Inhibitor? or Dipeptidyl-Peptidase type 4 Inhibitor? or DipeptidylPeptidase type IV Inhibitor? or DipeptidylPeptidase type 4 Inhibitor? or gliptin?).ti,ab.
49	(alogliptin or linagliptin or saxagliptin or sitagliptin or vildagliptin).ti,ab.
50	Thiazolidinediones/
51	(Thiazolidinedione? or glitazone?).ti,ab.
52	(pioglitazone or rosiglitazone).ti,ab.
53	(meglitinides? or nateglinide or repaglinide).ti,ab.
54	(Incretin mimetic? or glucagon-like peptide-1 activator? or GLP-1 activator?).ti,ab.
55	(exenatide or liraglutide or lixisenatide).ti,ab.
56	Sodium-Glucose Transporter 2/ai [Antagonists & Inhibitors]
57	(Sodium-glucose co-transporter 2 inhibitor? or SGLT2 inhibitor?).ti,ab.
58	(canagliflozin or dapagliflozin).ti,ab.
59	Hypoglycemic Agents/
60	(glyc?emic control adj3 (drug? or agent? or therap* or treatment)).ti.
61	exp Hydroxymethylglutaryl-CoA Reductase Inhibitors/
62	((hmg coa adj2 inhibitor?) or (Hydroxymethylglutaryl-CoA adj2 Inhibitors) or statin?).ti,ab.
63	(atorvastatin or fluvastatin or pravastatin or rosuvastatin or simvastatin or cerivastatin).ti,ab.
64	bile acid sequestran?.ti,ab.
65	(colesevelam or colestyramine or colestipol).ti,ab.
66	exp Fibric Acids/
67	(fibric acid? or fibrate?).ti,ab.

For peer review only - http://bmjopen.bmj.com/site/about/guidelines.xhtml

68	(bezafibrate or ciprofibrate or fenofibrate or gemfibrozil or clofibrate).ti,ab.
69	Nicotinic Acids/
70	(nicotinic acid? or acipimox).ti,ab.
71	exp *Fatty Acids, Omega-3/
72	(omega 3 fatty acid* or omega 3 marine triglyceride*).ti,ab.
73	(ezetimibe or lomitapide).ti,ab.
74	Hypolipidemic Agents/
75	((lipid lower* or hypolipidem* or cholesterol lower* or hypocholesterol*) adj2 (drug? or agent? or therap* or treatment)).ti,ab.
76	sodium bicarbonate/
77	sodium bicarbonate.ti,ab.
78	(Chortalidone or chorthalidone or chorpropamide or canaglifozin or dapaglifozin or canaglifloxin or dapaglifloxin or canaglifoxin or dapaglifoxin or giptin? or alogiptin or linagiptin or saxagiptin or sitagiptin or vildagiptin or Eposartan or Metopolol or popranolol or popranolole or propanalol or propanalole or propanolol or propranolole).ti,ab.
79	6 or 7 or 8 or 9 or 10 or 11 or 12 or 13 or 14 or 15 or 16 or 17 or 18 or 19 or 20 or 21 or 22 or 23 or 24 or 25 or 26 or 27 or 28 or 29 or 30 or 31 or 32 or 33 or 34 or 35 or 36 or 37 or 38 or 39 or 40 or 41 or 42 or 43 or 44 or 45 or 46 or 47 or 48 or 49 or 50 or 51 or 52 or 53 or 54 or 55 or 56 or 57 or 58 or 59 or 60 or 61 or 62 or 63 or 64 or 65 or 66 or 67 or 68 or 69 or 70 or 71 or 72 or 73 or 74 or 75 or 76 or 77 or 78
80	5 and 79
81	limit 80 to "reviews (maximizes specificity)"
82	randomized controlled trial.pt.
83	controlled clinical trial.pt.
84	randomized.ab.
85	placebo.ab.
86	drug therapy.fs.
87	randomly.ab.
88	trial.ab.
89	groups.ab.
90	82 or 83 or 84 or 85 or 86 or 87 or 88 or 89
91	exp animals/ not humans.sh.
92	90 not 91
93	80 and 92
94	limit 93 to yr="1999 -Current"
95	randomized controlled trial.pt.
96	controlled clinical trial.pt.
97	randomized.ab.
98	placebo.ab.
99	clinical trials as topic.sh.
100	randomly.ab.
101	trial.ti.
102	95 or 96 or 97 or 98 or 99 or 100 or 101
103	exp animals/ not humans.sh.
104	102 not 103

For peer review only - http://bmjopen.bmj.com/site/about/guidelines.xhtml

105	80 and 104
106	limit 105 to yr="1999 -Current"

For peer review only

For peer review only - http://bmjopen.bmj.com/site/about/guidelines.xhtml

Supplementary Tables

Table 1. Kidney Disease Outcomes Quality Initiative classification (Levy 2003)

Stages of CKD	eGFR (ml/min/1.73m^2)	Description
1	≥ 90	Normal or increased GFR, with other evidence of kidney damage
2	60–89	Slight decrease in GFR, with other evidence of kidney damage
3A	45–59	Moderate decrease in GFR, with or without other evidence of kidney damage
3B	30–44	
4	15–29	Severe decrease in GFR, with or without other evidence of kidney damage
5	< 15	Established renal failure

CKD – Chronic kidney disease; GFR – Glomerular filtration rate.

For peer review only

For peer review only - http://bmjopen.bmj.com/site/about/guidelines.xhtml

Table 2. Included drug list

Blood pressure lowering and/or Antihypertensive drugs	Angiotension-converting enzyme inhibitors and/or ACE inhibitors and/or ACEi	Benazepril
		Captopril
		Cilazapril
		Enalapril maleate
		Fosinopril sodium
		Imidapril hydrochlorine
		Lisinopril
		Moexipril hydrochoride
		Perindopril erbumine
		Perindopril arginine
		Quinapril
		Ramipril
		Trandolapril
	Angiotension-II receptor antagonists and/or ARBs and/or angiotension receptor blockers and/or sartans	Azilsartan medoxomil
		Candesartan
		Eprosartan
		Irbesartan
		Losartan potassium
		Olmesartan medoxomil
		Telmisartan
		Valsartan
	Thiazides and/or thiazide diuretics and/or thiazide-like diuretics	Bendroflumethiazide
		Chlortalidone or chlorthalidone
		Cyclopenthiazide
		Indapamide
		Metolazone
		Xipamide
		Chlorothiazide
		Hydrochlorothiazide
	Aldosterone antagonists	Eplerenone
		Spironolactone
	Beta-adrenoceptor antagonists and /or beta-blockers and/or beta-adrenoceptor blocking drugs and/or beta-adrenergic blockers	Propranolol hydrochloride
		Acebutolol
		Atenolol
		Bisoprolol flumarate
		Carvedilol
		Celiprolol hydrochloride
		Esmolol hydrochloride
		Labetalol hydrochloride
		Metoprolol tartrate
		Nadolol

For peer review only - http://bmjopen.bmj.com/site/about/guidelines.xhtml

		Nebivolol
		Oxprenolol hydrochloride
		Pindolol
		Sotalol hydrochloride
		Timolol Maleate
	Calcium channel blockers and/or calcium channel blocking drugs and/or calcium antagonists	Amlodipine
		Diltiazem hydrochloride
		Felodipine
		Isradipine
		Lacidipine
		Lercanidipine hydrochloride
		Nicardipine
		Nifedipine
		Verapamil hydrochloride
		Nimodipine
		Nisoldipine
	Alpha adrenoceptor antagonists and/or alpha-adrenoceptor blocking drugs and/or alpha blockers and/or alpha1 blockers and/or postsynaptic alpha blockers	Doxazosin
		Indoramin
		Prazosin
		Terazosin
	Alpha-2-adrenoceptor agonists and/or centrally acting antihypertensive drugs	Clonidine
		Methyldopa
	Imidazoline receptor antagonists and/or centrally acting antihypertensive drugs	Moxonidine
	Vasodilators	Hydralazine hydrochloride
		Minoxidil
Glycaemic control	Insulin	Insulin
		Insulin aspart
		Insulin lispro
		Insulin degludec
		Insulin detemir
		Insulin glargine
		Insulin zinc suspension
		Isophane insulin
		Protamine zinc insulin
		Biphasic insulin aspart
		Biphasic insulin lispro
		Biphasic isophane insulin
	Sulfonylureas	Glibenclamide
		Gliclazide
		Glimepiride

For peer review only

		Glipizide
		Tolbutamide
		Chlorpropamide
		Glibornuride
	Biguanides	Metformin hydrochloride
	Alpha glucosidase inhitors	Acarbose
	Dipeptidylpeptidase type 4 inhibitors and/or gliptins	Alogliptin
		Linagliptin
		Saxagliptin
		Sitagliptin
		Vildagliptin
	Thiazolidinediones and/or glitazones	Pioglitazone
		Rosiglitazone
	Meglitinides	Nateglinide
		Repaglinide
	Incretin mimetics and/or glucagon-like peptide-1 activators and/or GLP-1 activators	Exenatide
		Liraglutide
		Lixisenatide
	Sodium-glucose co-transporter 2 inhibitors and/or SGLT2 inhibitors	Canagliflozin
		Dapagliflozin
Lipid regulating drugs	Statins	Atorvastatin
		Fluvastatin
		Pravastatin
		Rosuvastatin
		Simvastatin
		Cerivastatin
	Bile acid sequestrants	Colesevelam hydrochloride
		Colestyramine
		Colestipol hydrochloride
	Fibrates	Bezafibrate
		Ciprofibrate
		Fenofibrate
		Gemfibrozil
		Clofibrate
	Nicotinic acid derivatives	Acipimox
		Nicotinic acid
	Omega 3 fatty acid compounds	Omega-3-acid ethyl esters
		Omega-3-marine triglycerides
	Miscellanous	Ezetimibe
		Lomitapide
Bicarbonate(s)	Sodium bicarbonate	

For peer review only

For peer review only - http://bmjopen.bmj.com/site/about/guidelines.xhtml

Reduction/Lowering of proteinuria	No specific drugs - usually additional response to blood pressure and glycaemic control drugs	

For peer review only

For peer review only - http://bmjopen.bmj.com/site/about/guidelines.xhtml

Table 3. Characteristics of studies

Study (trial name/author)	Year	Drug class	Trial N*	Analysis N*	Follow up (mths)**	CKD stages†	Population that we studied [method of obtaining data on subgroup for analysis]	Country
ADVANCE[79 80] §	2010 2012	A	10640	2184	60	3 and 4	Type II diabetes and, most likely, CKD stages 1 to 5. [Authors gave data for stages 3 and 4]	Multinational
Bianchi 2010[66]	2010	A	128	128	36	1 to 3	Idiopathic chronic glomerulonephritis and CKD stages 1 to 3	Italy
CHARM[38-40] (3 trials) §	2003 2006 2016	A	7599	463/845	26	3 and 4	Symptomatic chronic heart failure and, most likely, CKD stages 1 to 5. [Authors gave data for stages 3 and 4]	Multinational
EMPHASIS-HF[91 92]	2001 2013	A	2737	2737	21	1 to 3	Systolic heart failure and CKD stages 1, 2 and 3a.	Multinational
ESBARI[44] §	2010	A	224	116	41	4	Advanced chronic renal insufficiency without diabetes and CKD stages 4 and 5. [Authors gave data for stage 4]	China
HALT-PKD[52] §	2001	A	486	468	24	3 and 4	Moderately advanced autosomal dominant polycystic kidney disease and CKD stages 3 and 4. [Authors gave further data]	USA
HYVET[81-83] §	2001 2008 2012	A	3845	686	25	3	Very elderly hypertensive population and, most likely, CKD stages 1 to 5. [Authors gave data for stage 3]	Multinational
IDNT[53-56]	1992 2003 2011 2014	A	1715	995	36	3	Hypertensive and with diabetic nephropathy due to type II diabetes and CKD stages 1 to 5 [Stage 3 data was published]	USA
PREVEND IT[67]	2009	A/L‡	864	864	46	1 to 3	Microalbuminuria and CKD stages 1 to 3.	Netherlands
RENAAL[93-97]	2002 2004 2005 2006	A	1513	1513	41	2 to 4	Type II diabetic nephropathy and CKD stages 2 to 4.	Multinational

For peer review only - http://bmjopen.bmj.com/site/about/guidelines.xhtml

	2014							
SAVE[57] §	1998	A	2231	521	36	3a	Left ventricular dysfunction after M. CKD stages 1 to 5. [Authors gave data for stage 3a]	USA/Canada
SUPPORT[46 47]	2013 2016	A	1147	1147	53	1 to 4	Hypertensive with heart failure. CKD stages 1 to 4.	Japan
TOPCAT[98]	2007	A	3445	3445	40	1 to 3	Symptomatic heart failure and preserved ejection fraction.	Multinational
4S[68-70]	1993 2000 2003	L	4444	409	66	3	Coronary heart disease and elevated LDL and, most likely, CKD stages 1 to 5. [Stage 3 data was published]	Europe
AFCAPS/ TEXCAPS[58-60]	1997 2001 2010	L	6605	304	61	2 to 4	With CKD without significant diabetes or cardiovascular disease [Stage 3 and 4 data was published]	USA
ASCOT-LLA[71 72] §	2011 2007	L‡	10305	10305	40	1 to 3	Hypertension and, most likely, CKD stages 1 to 5. [Stage 1 to 3 data was published and authors gave further data]	Europe
ASUCA[48] §	2004	L	349	279/334	24	3 and 4	Dyslipidemia. [Stage 3 and 4 data was published and authors gave further data]	Japan
ATIC[73]	2008	L	93	93	24	2 to 4	Mild/moderate CKD.	Netherlands
FIELD[84-86]	2004 2005 2015	L	9795	519	60	3	Type II diabetes and renal impairment. CKD stages 1 to 3 [Stage 3 data was published]	Multinational
GISSI-HF[74 75] §	2004 2007	L	4631	1043	36	3 and 4	Chronic heart failure. [Obtained data on stages 3 and 4 from the company that owns the data]	Italy
GREACE[76 77]	2002 2009	L	1600	1600	36	1 to 3	Coronary heart disease, and CKD stages 1 to 3. [Authors reported a post-hoc analysis on those with and without metabolic syndrome]	Greece
LORD[45]	2015	L	123	123	29	2 to 4	CKD stages 2 to 4.	Tasmania

For peer review only - http://bmjopen.bmj.com/site/about/guidelines.xhtml

MEGA[49-51]	2006 2009 2014	L	7832	2978	64	3	Hypercholesterolemia and normal kidney function. CKD stages 1 to 3. [Stage 3 data was published]	Japan
METEOR[99]	1999	L	984	984	24	1 to 3	Asymptomatic individuals with moderately elevated cholesterol and low risk of cardiovascular disease.	Multinational
PROSPER[78 100 101]	2002 2007 2011	L	5796	3094	24	3 and 4	With pre-existing risk of vascular disease or risk of developing this condition. Most likely with CKD stages 1 to 5. [Stage 3 and 4 data was published]	Europe
SHARP[102-104]	2010 2014	L	9270	4808/4720	58	1 to 4	CKD stages 1 to 5. [Stage 1 to 4 data was published with graphical data separating Stages 1 to 3 with Stage 4 data]	Multinational
VA-HIT[61-63]	1993 2004 2017	L	399	399	24	3	Coronary disease and low HDL. Most likely CKD stages 1 to 5. [Stage 3 data was published]	USA
Bode 2015[105 106]	2013	G	714	503	24	1 to 3	Type II diabetes and CKD.	Multinational
CANVAS Program[19 20] (2 trials) §	2017 2018	G	10142	496¶	29	3	Two trials of populations with type II diabetes and high cardiovascular risk with CKD stages 1 to 4. [Stage 3a and 3b data was published]	Multinational
EMPA-REG OUTCOME[87 88]	2016	G	7020††	1819/7020	36	3 §§	Type II diabetes and CKD stages 1 to 3. [Stage 3 data was published]	Multinational
Kohan 2014[107]	2009	G	252	252	24	2 to 4	Type II diabetes and moderate renal impairment with CKD stages 2 to 4.	Multinational
Leiter 2016[41-43] (2 trials)	2006 2014 2015	G	1887	474	24	1 to 3	Two trials of populations with type II diabetes and cardiovascular disease with CKD stages 1 to 3. One of the trials also included participants with hypertension.	Multinational
TECOS[89 90]	2010 2015	G	14671	567/3321	36	3	Type II diabetes and cardiovascular disease and CKD stages 1 to 3. [Stage 3 data was published]	Multinational

For peer review only - http://bmjopen.bmj.com/site/about/guidelines.xhtml

Brito-Ashurst 2009[13]	2009	B	134	134	24	4	Stage 4 chronic CKD (creatinine clearance 15 to 30 ml/min per 1.73 m^2 and metabolic acidosis (serum bicarbonate 16 to 20 mmol/l)	UK
Goraya 2017[64 65]	2010 2014	B	108	108	60	3	Stage 3 CKD and metabolic acidosis (plasma TCO_2 22 to 24 mmol/l)	USA

* Trial population size (single or combined trials) and analysis population (if two numbers are given, the first refers to the primary outcome); ** mean or median; † CKD stages are given for the analysis population; †† 7028 patients were randomised; ‡ 2X2 factorial trial; §Unpublished data was provided by the authors; §§ - Used data for CKD stage 3 in the main analysis and data for CKD stages 1 to 3 as a sensitivity analysis; ¶- Included eGFR data from time before longest follow-up. CKD – chronic kidney disease. Drug class: A - antihypertensive; L – lipid-modifying; G – glycaemic control; B- sodium bicarbonate.

For peer review only

Table 4. Interventions of studies

Study	Year	Drug class	Drug type(s)	Interventions (dose and frequency, if stated)
ADVANCE[79 80]	2010<br>2012	A	ACE + thiazide	Perindopril (2 to 4 mg daily) + indapamide (0.625 to 1.25 mg daily) vs placebo
Bianchi 2010[66]	2010	A	ACE+ ARB+ aldost. antag. vs Thiazide+BB+ARB+CCA	Ramipril (initially 10 mg daily) + irbesartan (300 mg daily) + atorvastatin (20 mg daily) + spironolactone (25 mg daily) vs ramipril (10 mg daily) + atorvastatin (10 mg daily), possibly + hydrochlorothiazide or furosemide, calcium antagonists, beta-blockers or alpha-receptor antagonists.
CHARM[38-40]	2003<br>2006<br>2016	A	ARB	Candesartan (4 or 8 mg to 32 mg daily) vs placebo
EMPHASIS-HF[91 92]	2001<br>2013	A	Aldost. antag.	Eplerenone (25 to 50 mg daily) vs placebo
ESBARI[44]	2010	A	ACE	Benazepril (20 mg twice a day) vs placebo
HALT-PKD[52]	2001	A	ARB	Telmisartan (40 to 80 mg daily) vs placebo
HYVET[81-83]	2001<br>2008<br>2012	A	ACE + thiazide	Indapamide initially (1.5 to 2 or 4 mg daily) and possibly + perindopril later to achieve target blood pressure vs placebo
IDNT[53-56]	1992<br>2003<br>2011<br>2014	A	ARB<br>CCA	Irbesartan (75 to 100 mg daily) vs amlodipine (2.5 to 10 mg daily) vs placebo
PREVEND IT[67]	2009	A/L	Anti-hypertensive: ACE<br>Lipid modifying: Statin	2x2 factorial design: fosinopril (20 mg daily) or matching placebo and pravastatin (40 mg daily) or matching placebo<br>Data for antihypertensive analysis: (fosinopril+pravastatin or fosinopril+placebo) vs (placebo only or placebo + pravastatin)<br>Data for lipid-modifying analysis: (fosinopril+pravastatin or pravastatin+placebo) vs (placebo only or placebo + fosinopril)
RENAAL[93-97]	2002<br>2004<br>2005	A	ARB	Losartan (5 to 100 mg daily) vs placebo

For peer review only - http://bmjopen.bmj.com/site/about/guidelines.xhtml

	2006<br>2014			
SAVE[57]	1998	A	ACE	Captopril (6.5 to 50 mg 3 times a day) vs placebo
SUPPORT[46 47]	2013<br>2016	A	ARB	Olmesartan (5-10mg and increased to 40mg daily) vs placebo
TOPCAT[98]	2007	A	Aldost. antag.	Spironolactone (15-45 mg daily) vs placebo
4S[68-70]	1993<br>2000<br>2003	L	Statin	Simvastatin (20 to 40 mg daily) vs placebo
AFCAPS/TEXCAPS[58-60]	1997<br>2001<br>2010	L	Statin	Lovastatin (20 mg daily) vs placebo
ASCOT-LLA[71 72]	2011<br>2007	L	Statin	2x2 factorial design: atorvastatin (10 mg daily) + amlodipine (5 to 10 mg daily) vs atorvastatin (10 mg daily) + atenolol (50 to 100mg daily) vs placebo + amlodipine (5 to 10 mg daily) vs placebo + atenolol (50 to 100 mg daily)<br>Data for atenolol analysis: (atorvastatin + atenolol) vs (placebo + atenolol)<br>Data for amlodipine analysis: (atorvastatin + amlodipine) vs (placebo + amlodipine)
ASUCA[48]	2004	L	Statin	Atorvastatin (10 to 5-20 mg daily) vs diet
ATIC[73]	2008	L	Statin+ other	Pravastatin (40 mg daily) + vitamin E (300 mg daily) + folic acid (15 mg daily) + pyridoxine hydrochloride (100 mg daily) + cyanacobalamin (1 mg daily) vs placebo
FIELD[84-86]	2004<br>2005<br>2015	L	Fibrate	Fenofibrate (200 mg daily) vs placebo
GISSI-HF[74 75]	2004<br>2007	L	Statin	Rosuvastatin (10mg) daily vs placebo
GREACE[76 77]	2002<br>2009	L	Statin	Atorvastatin (10 to 80 mg daily) vs usual care
LORD[45]	2015	L	Statin	Atorvastatin (10 mg daily) vs placebo
MEGA[49-51]	2006<br>2009	L	Statin	Pravastatin (10 mg initially) + diet vs diet

For peer review only - http://bmjopen.bmj.com/site/about/guidelines.xhtml

	2014			
METEOR[99]	1999	L	Statin	Rosuvastatin (40 mg daily) vs placebo
PROSPER[78 100 101]	2002<br>2007<br>2011	L	Statin	Pravastatin (40 mg daily) vs placebo
SHARP[102-104]	2010<br>2014	L	Statin	Simvastatin (20 mg daily) + ezetimibe (10 mg daily) vs placebo
VA-HIT[61-63]	1993<br>2004<br>2017	L	Fibrate	Gemfibrozil (1200 mg daily) vs placebo
Bode 2015[105 106]	2013	G	Flozin	Canagliflozin (100 mg daily) vs canagliflozin (300 mg daily) vs placebo
CANVAS Program[19 20]	2017<br>2018	G	Flozin	Canagliflozin (100mg-300mg) vs placebo
EMPA-REG OUTCOME[87 88]	2016	G	Flozin	Empagliflozin (25mg daily) vs empagliflozin (10mg daily) vs placebo
Kohan 2014[107]	2009	G	Flozin	Dapagliflozin (10 mg daily) vs dapagliflozin (50 mg daily) vs placebo
Leiter 2016[41-43]*	2006<br>2014<br>2015	G	Flozin	Dapagliflozin (10 mg daily) vs placebo
TECOS[89 90]	2010<br>2015	G	Gliptin	Sitagliptin (100 mg daily) vs placebo
Brito-Ashurst 2009[13]	2009	B	Sodium bicarbonate	Oral sodium bicarbonate (600 mg 3 times a day) vs routine standard care
Goraya 2017[64 65]	2010<br>2014	B	Sodium bicarbonate	Oral sodium bicarbonate (> 22 mq/L) vs routine care

* Reported in Leiter 2014 and Cefalu 2015. Drug class: A – antihypertensive; L – lipid-modifying; G – glycaemic control; B – sodium bicarbonate. Drug subclass: ACE – angiotension converting enzyme inhibitor; ARB - angiotensin II receptor blockers; Aldost. antag. - aldosterone antagonists; BB – beta-blocker; CCA – calcium channel antagonist.

For peer review only - http://bmjopen.bmj.com/site/about/guidelines.xhtml

For peer review only

Table 5. Baseline characteristics

Trial	Trial N	Baseline N	Analysis N	CKD stages*	Drug class	Age Mean (SD)	Male (%)	Non-white ethnicity (%)	Current Smoker (%)	Body mass index Mean (SD)	Systolic blood pressure Mean (SD)	Diastolic blood pressure Mean (SD)
Trials for which baseline data was provided for all patients included in the analysis												
ADVANCE[79 80]	10640	2184	2184/ 2033	3 to 4	A	68.2 (6.4)	43.1	NS	10.4	28.7 (5.2)	147.0 (23.3)	79.8 (11.3)
Bianchi 2010[66]	128	128	128	1 to 3	A	53.2 (0.9)	64.1	NS	28.9	25.1 (0.3)	156.1 (1.3)	93.7 (0.7)
EMPHASIS-HF[91 92]	2737	2737	2737	1 to 3	A	68.6 (7.6)	77.5	17.1	NS	27.5 (4.9)	124.0 (17.0)	75.0 (10.0)
PREVEND IT[67] (A)	864	864	864	1 to 3	A	51.3 (11.8)	64.9	3.9	39.9	26.0 (4.5)	130.0 (17.5)	76.0 (10.0)
PREVEND IT[67] (L)	864	864	864	1 to 3	L	51.3 (11.8)	65.0	3.9	38.2	26.0 (4.0)	130.5 (17.5)	76.5 (10.0)
RENAAL[93-97]	1513	1513	1513	2 to 4	A	60.0 (7.0)	63.2	51.3	18.3	29.5 (6.0)	152.5 (19.5)	82.0 (10.5)
TOPCAT[98]	3445	3445	3445	1 to 3	A	68.7 (11.2)	48.5	11.1	10.5	31 (6.7)	130 (14.5)	80.0 (7.4)
4S[68-70]	4444	409	409	3	L	62.2 (5.0)	54.5	NS	16.1	25.9 (3.4)	143.1 (21.4)	83.7 (9.6)
AFCAPS/ TEXCAPS[58-60]	6605	304	304	2 to 4	L	62.0 (7.5)	78.3	1.0	7.9	26.5 (3.0)	141.6 (18.0)	79.0 (10.0)
ATIC[73]	93	93	93	2 to 4	L	53.0 (12.0)	56.9	NS	35.5	26.5 (4.5)	135.0 (20.9)	78.5 (12.0)
FIELD[84-86]	9795	519	519	3	L	66.5 (5.9)	41.2	NS	6.9	NS	NS	NS
GREACE[76 77]	1600	1600	1600	1 to 3	L	58.5 (7.5)	79.4	NS	NS	25.8 (4.4)	129.0 (16.9)	77.5 (7.9)
LORD[45]	123	123	123	2 to 4	L	60.2 (15.1)	65.0	0	NS	28.5 (6.0)	143.4 (19.8)	80.7 (12.6)
MEGA[49-51]	7832	2978	2978	3	L	60.0	24.3	NS	12.5	NS	132.9	NS
PROSPER[78 100 101]	5796	3094	3094	3	L	75.5 (3.4)	42.0	NS	22.9	27.0 (4.3)	154.7 (21.6)	83.7 (11.3)
VA-HIT[61-63]	2531	399	399	3	L	67.3 (5.2)	100	9.5	14.0	NS	134.0 (18.3)	77.2 (9.8)
Kohan 2014[107]	252	252	252	2 to 4	G	67.0 (8.1)	65.1	16.3	NS	NS	132.1 (16.4)	73.3 (9.3)
EMPA-REG OUTCOME[87 88]	7020†	1819	1819	3	G	67.1 (7.8)	67.8	NS	NS	31.0 (5.5)	136.2 (18.2)	74.5 (10.0)
Brito-Ashurst 2009[13]	134	134	134	4	B	54.8 (2.4)	51.5	48.0	NS	NS	123.9 (1.3)	75.8 (1.7)
Goraya 2017[64 65]	108	108	108	3	B	53.7 (5.1)	44.0	NS	NS	NS	162.3 (11.1)	NS

For peer review only - http://bmjopen.bmj.com/site/about/guidelines.xhtml

Trials for which baseline data was not provided for all patients included in the analysis but for a different subgroup of the trial population or the whole trial population(s)												
CHARM[38-40] (3 trials)	7599	2680	463/845	3 to 4	A	65.3 (11.6)	66.6	NS	14.2	NS	128.2 (18.7)	73.6 (10.7)
ESBARI[44]	224	224	116/224	4	A	44.7 (15.5)	49.5	NS	NS	22.8 (5.0)	152.4 (23.1)	85.9 (10.4)
HYVET[81-83]	3845	1194	686	3	A	83.8 (3.3)	31.0	NS	NS	25.0 (3.8)	173.7 (8.9)	NS
HALT-PKD[52]	486	486	468	3 to 4	A	48.7 (8.3)	48.3	6.5	NS	28.0 (5.2)	121.5 (15.6)	75.9 (10.9)
IDNT[53-56]	1715	1715	995	3	A	58.9 (7.8)	66.3	27.5	NS	30.8 (5.8)	159.0 (19.7)	87.0 (11.0)
SAVE[57]	2231	2231	521	3a	A	59.4	82.5	NS	NS	NS	112.5	70.0
SUPPORT[46 47]	1147	1147	1139	1 to 4	A	65.7 (10.2)	74.7	NS	NS	24.4 (4.1)	127.9 (18.1)	74.4 (12.0)
ASCOT-LLA[71 72]	10305	10305	10305	1 to 3	L	63.1 (8.5)	81.2	NS	32.7	23.6 (6.9)	164.2 (17.8)	95.0 (10.3)
ASUCA[48]	349	334	279/334	3 to 4	L	63.2 (8.1)	63.8	NS	14.1	25.6 (3.6)	133.2 (16.3)	76.8 (10.6)
GISSI-HF[74 75]	4631	4574	1043	3 to 4	L	68.0 (11.0)	77.4	NS	14.0	27.1 (4.5)	127.0 (18.0)	77.0 (10.0)
METEOR[99]	984	984	900	1 to 3	L	57.0 (6.0)	59.7	15.7	4.0	27.2 (4.0)	139.0 (21.0)	80.0 (12.0)
SHARP[102-104]	9270	6245	4808/ 4720	1 to 4	L	63.0 (12.0)	62.5	28.5	12.0	27.4 (5.5)	139.0 (21.0)	80.0 (12.0)
Bode 2015[105 106]	714	714	503	1 to 3	G	63.6 (6.2)	55.5	23.1	NS	31.6 (4.6)	NS	NS
CANVAS Program[19 20] (2 trials)	10142	10142	496	3	G	63.3 (8.3)	64.2	21.7	17.8	32.0 (5.9)	136.6 (15.8)	77.7 (9.7)
Leiter 2016[41-43] (2 trials)	1887	568	474/568	1 to 3	G	62.9 (7.3)	93.9	NS	NS	32.7 (5.5)	133.8 (13.5)	77.6 (8.5)
TECOS[89 90]	14671	3321	567/332 1	3	G	68.3 (7.8)	63.6	68.7	7.4	30.5 (5.8)	136.0 (18.0)	76.7 (10.7)

NS – not stated; * CKD stages are given for the analysis population; Drug class: A – anti-hypertensive; L – lipid modifying; G – glycaemic control; B- sodium bicarbonate.

For peer review only

For peer review only - http://bmjopen.bmj.com/site/about/guidelines.xhtml

Table 6. Results of analyses

	Effect measure (95% CI)	No of studies or populations*	I^2
**1. Estimated Glomerular Filtration Rate**			
Antihypertensive drugs			
Random effects, DerSimonian and Laird method (Base Case)	1.03 (0.96 to 1.11)	9	95.3%
Fixed effects, inverse-variance method	1.01 (0.99 to 1.02)	9	95.3%
Hartung-Knapp-Sidik-Jonkman method	1.03 (0.93 to 1.14)	9	95.3%
Excluding studies with estimates	0.98 (0.94 to 1.03)	7	81.0%
Excluding studies with high risk of attrition or allocation bias	1.03 (0.95 to 1.13)	8	95.8%
Using CKD EPI data for CHARM trial	1.03 (0.96 to 1.11)	9	95.1%
More conservative imputation, using a larger SE	1.03 (0.97 to 1.10)	9	92.5%
Adding studies with CKD stage 1/2 patients	1.05 (0.99 to 1.11)	12	97.0%
Adding studies with CKD stage 1/2 patients and with placebo controls only	1.04 (0.98 to 1.10)	11	96.0%
Hartung-Knapp-Sidik-Jonkman method and studies with CKD stage 1/2 patients	1.05 (0.97 to 1.13)	12	97.0%
Lipid modifying drugs			
Random effects, DerSimonian and Laird method (Base Case)	1.04 (1.0001 to 1.08)	5	88.3%
Fixed effects, inverse-variance method	1.02 (1.01 to 1.03)	5	88.3%
Hartung-Knapp-Sidik-Jonkman method	1.04 (0.98 to 1.10)	5	88.3%
Excluding studies with non-placebo controls	1.05 (0.97 to 1.14)	3	93.8%
Excluding studies with estimates (SHARPb)	1.01 (0.99 to 1.03)	4	45.3%
Excluding studies with high risk of attrition or allocation bias	1.01 (1.003 to 1.02)	2	0%
More conservative imputation, using a larger SE	1.03 (0.999 to 1.06)	5	73.9%
Adding studies with CKD stage 1/2 patients	1.09 (1.03 to 1.15)	13	98.0%
Adding studies with CKD stage 1/2 patients and excluding studies with non-placebo controls	1.09 (1.004 to 1.18)	9	98.1%
Hartung-Knapp-Sidik-Jonkman method and adding studies with CKD stage 1/2 patients	1.09 (1.02 to 1.16)	13	98.0%
Glycaemic control drugs			
Random effects, DerSimonian and Laird method (Base Case)	1.06 (1.02 to 1.10)	6	0%
Fixed effects, inverse-variance method	No change		

For peer review only - http://bmjopen.bmj.com/site/about/guidelines.xhtml

Hartung-Knapp-Sidik-Jonkman method	1.06 (1.01 to 1.11)	6	0%
Excluding studies with estimates	0.96 (0.69 to 1.34)	2	0%
Excluding studies with high risk of attrition or allocation bias	No change		
More conservative imputation, using a larger SE	No change		
Adding studies with CKD stage 1/2 patients	0.94 (0.85 to 1.05)	11	85.1%
Hartung-Knapp-Sidik-Jonkman method and studies with CKD stage 1/2 patients	0.94 (0.80 to 1.12)	11	85.1%
CKD stage 1 to 3 data for EMPA-REG and adding studies with stage 1/2 patients	0.93 (0.82 to 1.04)	11	81.4%
Excluding Leiter 2016 (outlier) and Adding studies with CKD stage 1/2 patients	1.04 (1.01 to 1.07)	10	0%
Sodium bicarbonate			
Random effects, DerSimonian and Laird method	1.08 (0.84 to 1.38)	2	91.1%
**2. Proteinuria**			
Antihypertensive drugs			
Random effects, DerSimonian and Laird method (Base Case)	0.91 (0.82 to 1.01)	3	41.3%
Fixed effects to inverse-variance method	No change		
Excluding studies with estimates	0.80 (0.67 to 0.95)	1	-
Excluding studies of PER and PCR	0.98 (0.86 to 1.12)	2	0%
Excluding studies with high risk of attrition or allocation bias	No change		
More conservative imputation to using a larger SE	No change		
Adding studies with CKD stage 1/2 patients	0.74 (0.53 to 1.03)	5	94.5%
**3. Adverse events**			
Anti-hypertensive drugs			
HALT-PKD, CKD stages 3-4	0.89 (0.74 to 1.07)	1	-
Adding studies with CKD stage 1/2 patients	0.94 (0.83 to 1.05)	4	58.0%
Lipid modifying drugs			
Random effects, DerSimonian and Laird method (Base Case)	0.91 (0.78 to 1.06)	3	17.8%
Fixed effects to Mantel–Haenszel method	0.92 (0.86 to 0.98)	3	17.8%
Excluding studies with high risk of attrition or allocation bias	0.96 (0.52 to 1.79)	2	55.8%
Adding studies with CKD stage 1/2 patients	1.02 (0.79 to 1.32	5	55.3%
Glycaemic control drugs			

For peer review only - http://bmjopen.bmj.com/site/about/guidelines.xhtml

EMPA-REG	0.96 (0.94 to 0.99)	1	-
Adding studies with CKD stage 1/2 patients	1.01 (0.96 to 1.06)	6	62.7%
**4. Combined maintenance dialysis and kidney transplantation**			
Anti-hypertensive drugs			
HALT-PKD	0.69 (0.50 to 0.96)	1	-
Adding RENAAL (CKD stages 2 to 4)	0.75 (0.64 to 0.88)	2	0%
Lipid modifying drugs			
Random effects, DerSimonian and Laird method (Base Case)	0.97 (0.88 to 1.08)	3	0%
Fixed effects to Mantel–Haenszel method	No change		
Excluding studies with high risk of attrition or allocation bias	1.20 (0.34 to 4.25)	2	0%
Adding studies with stages 1-4 CKD	0.97 (0.88 to 1.07)	5	7.8%
Glycaemic control drugs			
EMPA-REG	0.78 (0.61 to 1.01)	1	-
Adding studies with CKD stage 1/2 patients	0.64 (0.27 to 1.53)	4	38.2%
**5. Cardiovascular events**			
Antihypertensive drugs			
Random effects, DerSimonian and Laird method (Base Case)	0.66 (0.33 to 1.34)	2 (with 1 excluded)	-
Fixed effects to Mantel–Haenszel method	No change		
Excluding studies with high risk of attrition or allocation bias	No change		
Adding studies with CKD stage 1/2 patients	0.92 (0.80 to 1.06)	4 (1 excluded)	0%
Lipid modifying drugs			
Random effects, DerSimonian and Laird method (Base Case)	0.64 (0.52 to 0.80)	5	29.6%
Fixed effects to Mantel–Haenszel method	0.65 (0.56 to 0.76)	5	29.6%
Excluding studies with high risk of attrition or allocation bias	0.66 (0.52 to 0.83)	4	35.1%
Excluding non-ITT study (AFCAPS/TEXCAPS)	0.66 (0.52 to 0.83)	4	35.1%
Adding studies with CKD stage 1/2 patients	0.60 (0.50 to 0.72)	11	59.0%
Glycaemic control drugs			
TECOS (CKD stage 3)	0.92 (0.79 to 1.08)	1	-
**6. Cardiovascular mortality**			
Antihypertensive drugs			
Random effects, DerSimonian and Laird method (Base Case)	0.79 (0.58 to 1.07)	2	0%
Fixed effects to Mantel–Haenszel method	0.78 (0.58 to 1.07)	2	0%

For peer review only

Excluding studies with high risk of attrition or allocation bias	No change		
Adding studies with CKD stage 1/2 patients	0.93 (0.80 to 1.08)	7	30.0%
Lipid modifying drugs			
Random effects, DerSimonian and Laird method (Base Case)	0.52 (0.30 to 0.92)	2	0%
Fixed effects to Mantel–Haenszel method	0.52 (0.29 to 0.91)	2	0%
Excluding studies with high risk of attrition or allocation bias	0.53 (0.30 to 0.93)	1	-
Excluding non-ITT study (AFCAPS/TEXCAPS)	0.53 (0.30 to 0.93)	1	-
Adding studies with CKD stage 1/2 patients	0.58 (0.35 to 0.92)	5	0%
Glycaemic control drugs			
Random effects, DerSimonian and Laird method (Base Case)	0.74 (0.53 to 1.02)	2	77.8%
Fixed effects to Mantel–Haenszel method	0.74 (0.63 to 0.86)	2	77.8%
Excluding studies with high risk of attrition or allocation bias	No change		
Adding studies with CKD stage 1/2 patients	0.73 (0.57 to 0.93)	4	37.2%
**7. All-cause mortality**			
Antihypertensive drugs			
Random effects, DerSimonian and Laird method (Base Case)	0.93 (0.81 to 1.08)	3	0%
Fixed effects to Mantel–Haenszel method	0.92 (0.80 to 1.07)	3	0%
Excluding studies with high risk of attrition or allocation bias	No change		
Adding studies with CKD stage 1/2 patients	0.94 (0.87 to 1.02)	8	31.0%
Lipid modifying drugs			
Random effects, DerSimonian and Laird method (Base Case)	0.74 (0.56 to 0.98)	4	0%
Fixed effects to Mantel–Haenszel method	0.72 (0.54 to 0.95)	4	0%
Excluding studies with high risk of attrition or allocation bias	No change		
Adding studies with CKD stage 1/2 patients	0.74 (0.59 to 0.92)	9	24.6%
Glycaemic control drugs			
Random effects, DerSimonian and Laird method (Base Case)	0.88 (0.74 to 1.04)	2	32.2%
Fixed effects to Mantel–Haenszel method	No change		
Excluding studies with high risk of attrition or allocation bias	No change		
Adding studies with CKD stage 1/2 patients	0.86 (0.73 to 1.02)	7	0%

For peer review only - http://bmjopen.bmj.com/site/about/guidelines.xhtml

Table 7. GRADE assessments of primary outcome for each drug group

Effects of antihypertensives. lipid-modifying drugs, glacaemic control drugs and sodium bicarbonate on the progression of stages 3 and 4 chronic kidney disease in adults

Patient or population: patients with chronic kidney disease (CKD)
Settings: primary care and shared care with specialist nephrology services
Intervention: drug
Comparison: placebo drug, no drug intervention, or a comparator drug from the three other classes

Outcomes	Illustrative comparative risks* (95% CI)		Relative effect (95% CI)	No of Participants (studies)	Quality of the evidence (GRADE)	Comments
	Assumed risk	Corresponding risk				
	**Placebo drug, no drug intervention, or a comparator drug from the three other classes**	**Drug**				
**eGFR (antihypertensives)** Follow-up: 24 to 62 months	See comment	See comment		5433 (7 studies[1])	⊕⊖⊖⊖ **very low**[2,3,4,5,6]	Ratio of means 1.03, 95% CI 0.96 to 1.11[7]
**eGFR (lipid-modifying drugs)** Follow-up: 24 to 64 months	See comment	See comment		6865 (4 studies[8])	⊕⊖⊖⊖ **very low**[4,6,9,10]	Ratio of means 1.04, 95% CI 1.00 to 1.08[7,11]
**eGFR (glycaemic control drugs)** Follow-up: 29 to 36 months	See comment	See comment		1438 (3 studies[12])	⊕⊕⊖⊖ **low**[4,13,14]	Ratio of means 1.06, 95% CI 1.02 to 1.10 [7]
**eGFR (sodium bicarbonate)** Follow-up: 24 to 36 months	See comment	See comment		180 (2 studies)	⊕⊖⊖⊖ **very low**[4,14,15,16,17]	Ratio of means 1.0, 95% CI 0.84 to 1.38 [7,18]

*The basis for the **assumed risk** (e.g. the median control group risk across studies) is provided in footnotes. The **corresponding risk** (and its 95% confidence interval) is based on the assumed risk in the comparison group and the **relative effect** of the intervention (and its 95% CI).

CI: Confidence interval;

GRADE Working Group grades of evidence
High quality: Further research is very unlikely to change our confidence in the estimate of effect.
Moderate quality: Further research is likely to have an important impact on our confidence in the estimate of effect and may change the estimate.
Low quality: Further research is very likely to have an important impact on our confidence in the estimate of effect and is likely to change the estimate.
Very low quality: We are very uncertain about the estimate.

[1] 9 datasets
[2] Risk of bias: Only one study was high risk both for incomplete outcome data and selective reporting. For all other risk of bias categories studies were assessed as low or unclear risk of bias.
[3] Inconsistency: there is a wide variation in study and participant characteristics. Point estimates for studies both indicated drug increasing and decreasing progression of CKD. Minimal overlap of confidence intervals. Substantial heterogeneity (I-squared 95.3%, p-value 0.000).
[4] Indirectness: Effect of drug on CKD was secondary to specified study outcomes.
[5] Imprecision: 95% confidence interval includes no effect
[6] Publication bias: few studies in the area of non-significance (contour enhanced funnel plots). Note: funnel plots also included studies which had populations mixed with stage 1 and 2 CKD.
[7] Weighted average of the mean eGFR in the intervention group divided by the mean eGFR in the control group. Ratio of means >1 favoured the intervention group for eGFR.

For peer review only - http://bmjopen.bmj.com/site/about/guidelines.xhtml

[8] 5 datasets
[9] Risk of bias: one study was high risk for blinding of participants, personnel and outcome assessment, One study (two datasets) was high risk of bias for incomplete outcome data. For all other risk of bias categories studies were assessed as low or unclear.
[10] Inconsistency: there a wide variation in study and participant characteristics. Point estimates, bar one study, show drug decreasing the progression of CKD. Substantial heterogeneity (I-squared 88.3%, p-value 0.000).
[11] STATA's metaninf command produced output that indicated that the lower confidence bound was 1.0001 to 4 d.p.
[12] 6 datasets
[13] Risk of bias: one study was high risk for selective reporting. For all other risk of bias categories studies were assessed predominantly as low.
[14] Publication bias: insufficient data to assess.
[15] Risk of bias: both studies were high risk for blinding, plus one study for allocation concealment. For all other risk of bias categories studies were assessed as low or unclear.
[16] Inconsistency: there is wide variation in study and participant characteristics. The point estimates of the two studies were either side of the line of no effect. No overlap of confidence intervals. Substantial heterogeneity (I-squared 91.1%, p-value 0.000).
[17] Imprecision: the total sample size is less than 300. 95% confidence interval includes no effect.
[18] Data pooled, though sparse, to provide an overall indication of treatment effect and allow comparison between drug classes

For peer review only

For peer review only - http://bmjopen.bmj.com/site/about/guidelines.xhtml

Supplementary Figures

Figure 1. Kidney Disease Improving Global outcomes (KDIGO) 2012

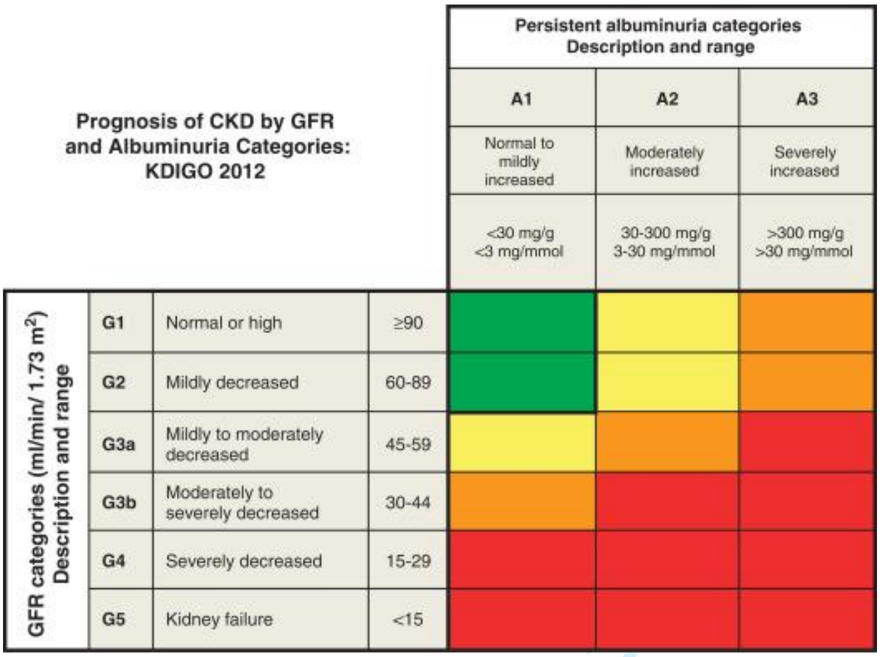

Albuminuria = proteinuria. Green: low risk (if no other markers of disease, no CKD); yellow moderately increased risk; orange: high risk; red: very high risk

(Reproduced with permission)

view only

For peer review only - http://bmjopen.bmj.com/site/about/guidelines.xhtml

Figure 2. Cochrane Collaboration's Risk of bias

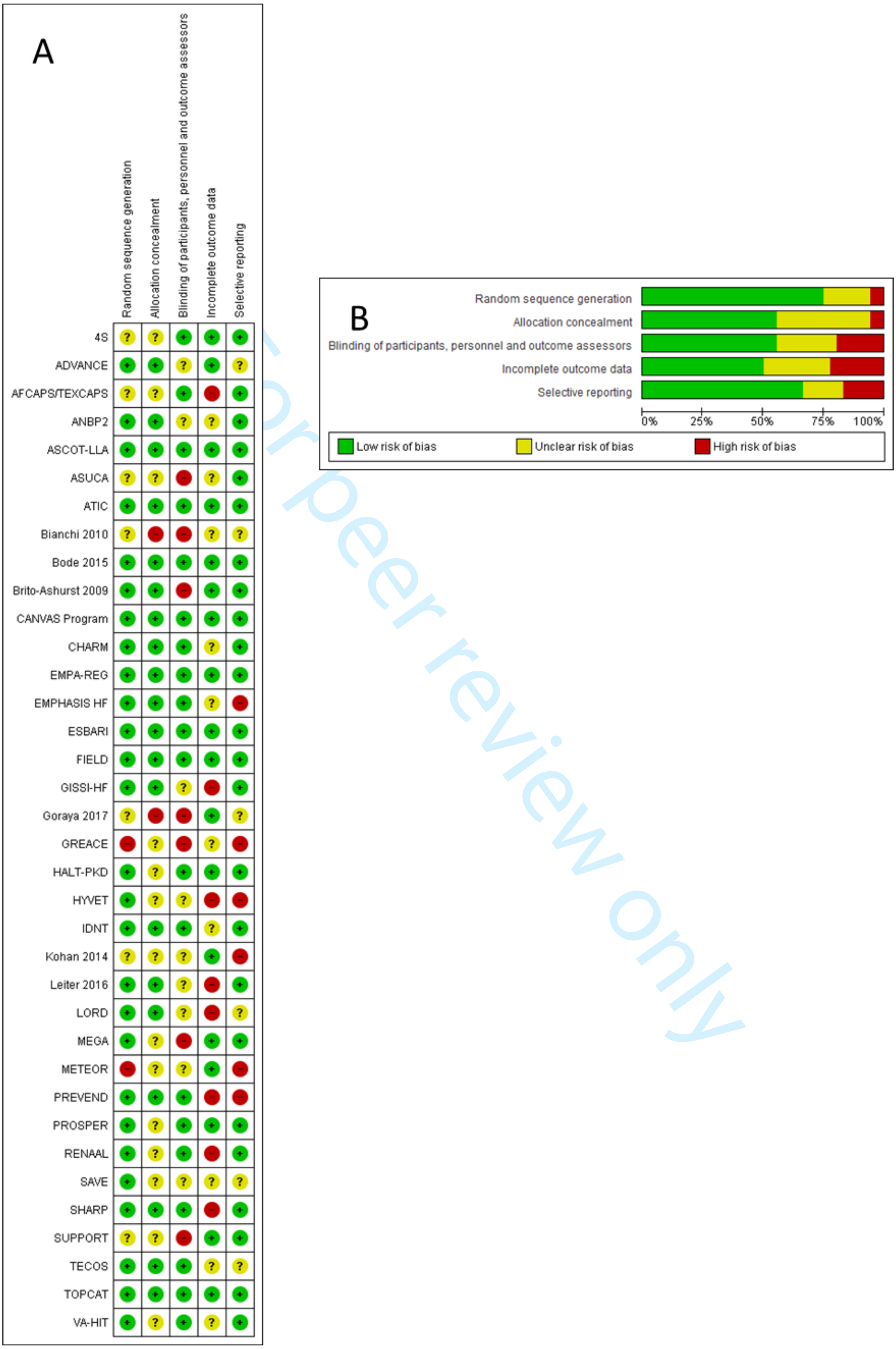

For peer review only

Figure 3. Funnel plots and contour enhanced funnel plots, for estimated glomerular filtration rate data, split by drug group

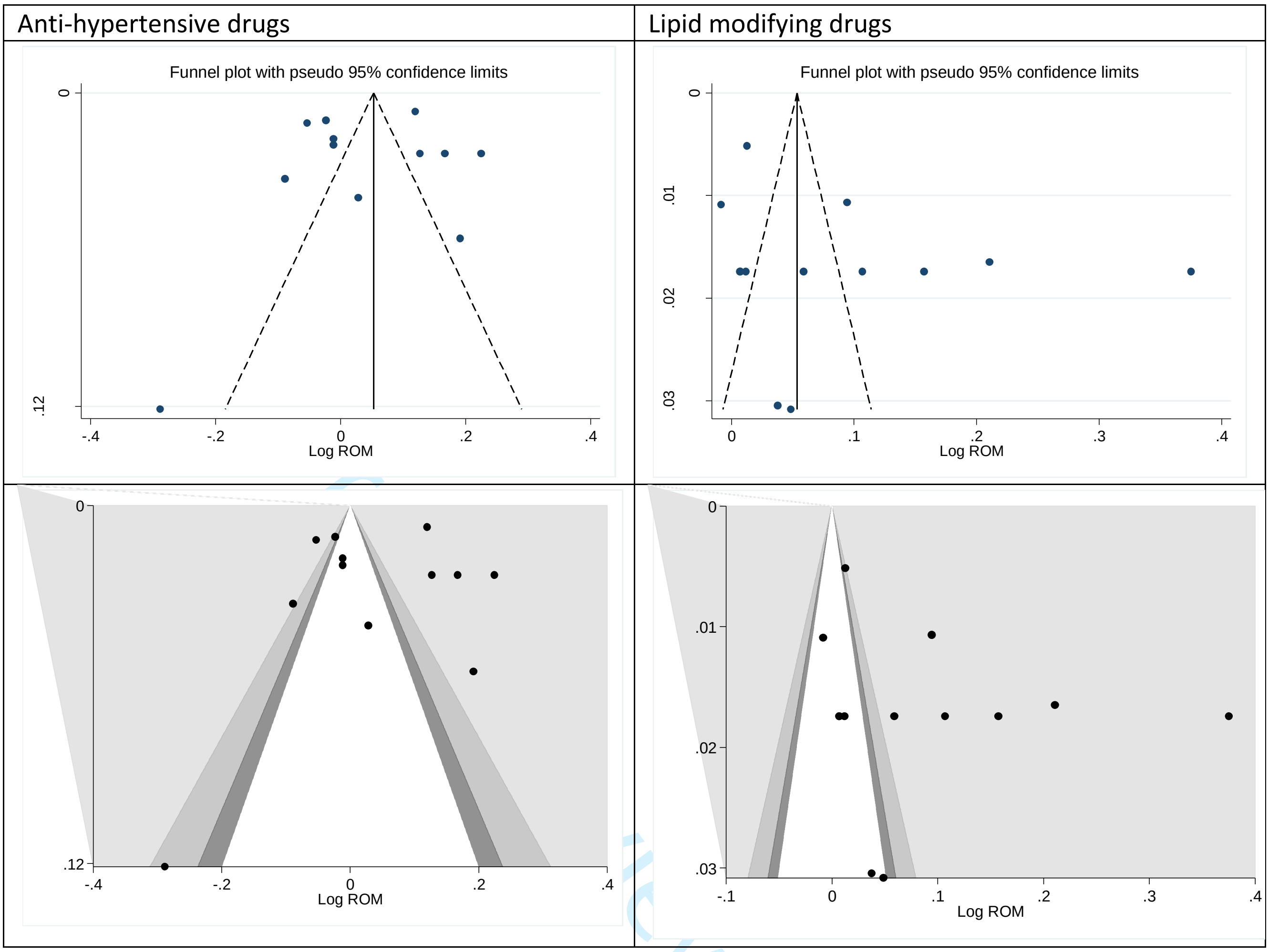

A positive Log ROM favours the intervention drug. Log refers to the natural logarithm.

Contour plots shading: light grey (p<1%); medium grey (1%<p<5%); dark grey (5%<p<10%); white (p>10%)

For peer review only - http://bmjopen.bmj.com/site/about/guidelines.xhtml

Figure 4. Ratio of means of estimated glomerular filtration rate at the end of the trials for anti-hypertensives vs comparator (boxes) and pooled estimates across studies (diamonds) calculated by the random effects DerSimonian and Laird method, for chronic kidney disease stages 3 and 4 and split by drug class

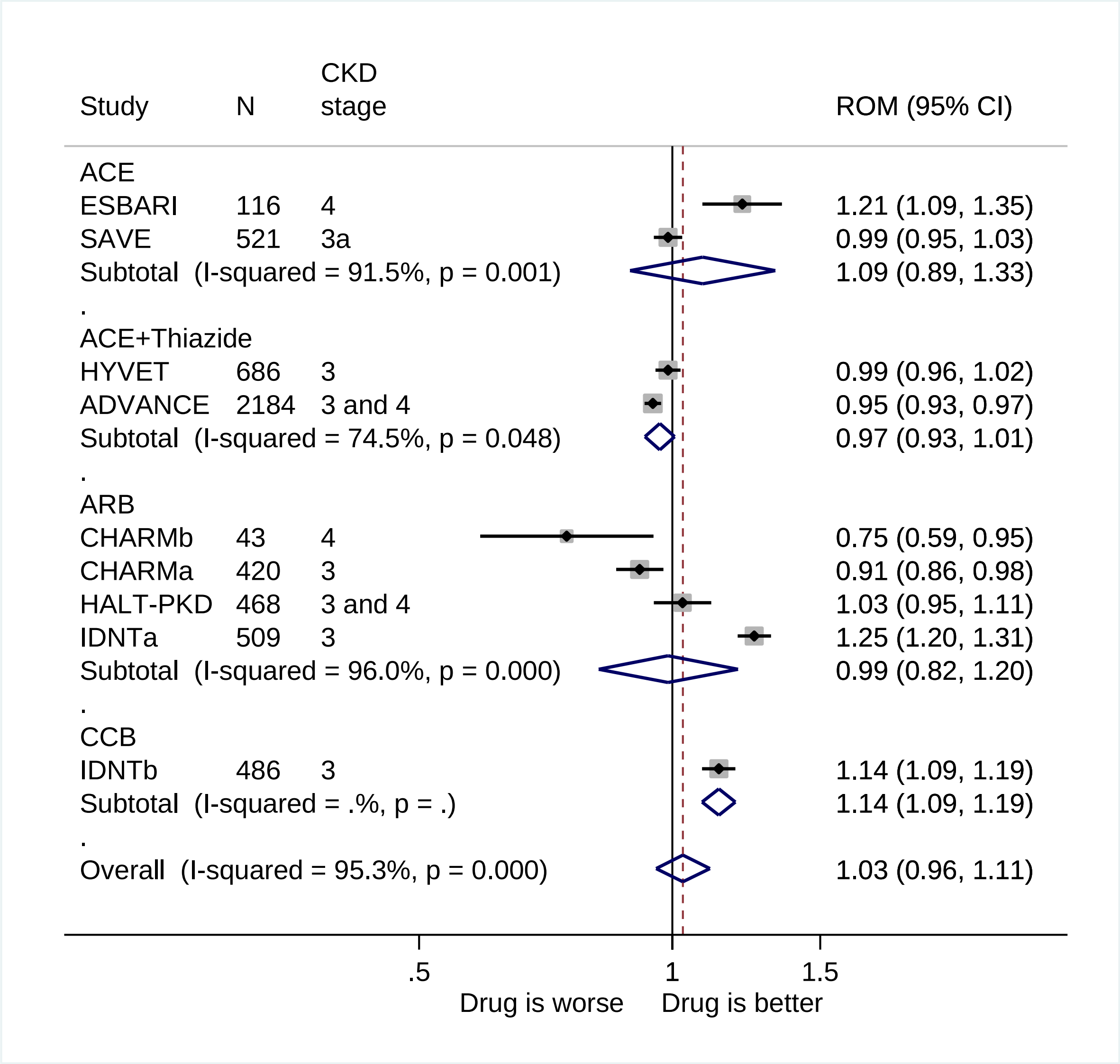

CKD – chronic kidney disease; CHARMa and CHARMb – CKD stage 3 and 4 respectively with eGFR measured by MDRD equation; IDNTa - intervention is Irbesartan; IDNTb - intervention is Amlodipine.

For peer review only - http://bmjopen.bmj.com/site/about/guidelines.xhtml

Figure 5. Ratio of means of proteinuria at the end of the trials for anti-hypertensives vs comparator (boxes) and pooled estimates across studies (diamonds) calculated by the random effects DerSimonian and Laird method, split by chronic kidney disease stage

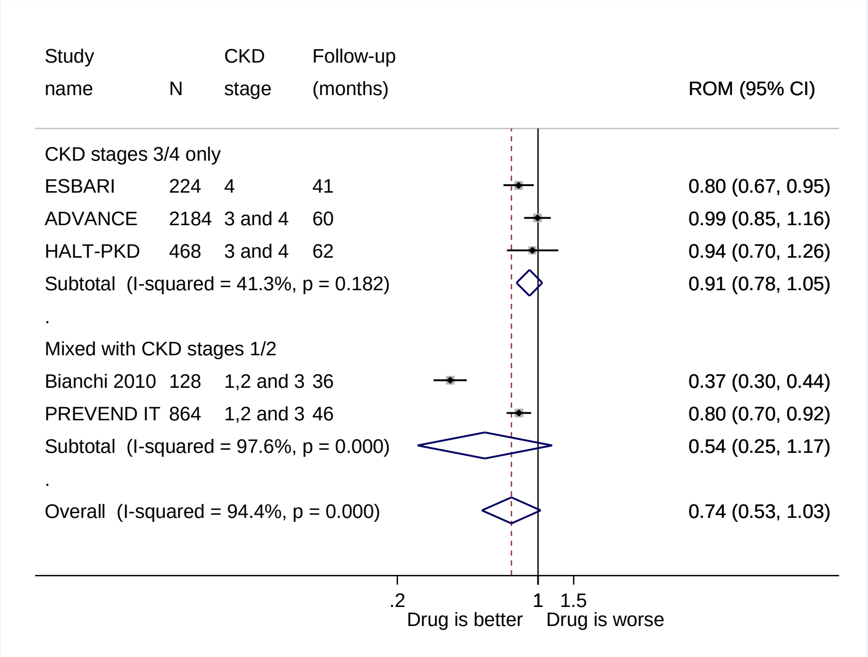

CKD – chronic kidney disease. Prediction interval for overall (0.20 to 2.70).

review only

For peer review only - http://bmjopen.bmj.com/site/about/guidelines.xhtml

PRISMA 2009 Checklist

Section/topic	#	Checklist item	Reported on page #
**TITLE**			
Title	1	Identify the report as a systematic review, meta-analysis, or both.	1
**ABSTRACT**			
Structured summary	2	Provide a structured summary including, as applicable: background; objectives; data sources; study eligibility criteria, participants, and interventions; study appraisal and synthesis methods; results; limitations; conclusions and implications of key findings; systematic review registration number.	3-4
**INTRODUCTION**			
Rationale	3	Describe the rationale for the review in the context of what is already known.	5-6
Objectives	4	Provide an explicit statement of questions being addressed with reference to participants, interventions, comparisons, outcomes, and study design (PICOS).	6
**METHODS**			
Protocol and registration	5	Indicate if a review protocol exists, if and where it can be accessed (e.g., Web address), and, if available, provide registration information including registration number.	4
Eligibility criteria	6	Specify study characteristics (e.g., PICOS, length of follow-up) and report characteristics (e.g., years considered, language, publication status) used as criteria for eligibility, giving rationale.	7-8
Information sources	7	Describe all information sources (e.g., databases with dates of coverage, contact with study authors to identify additional studies) in the search and date last searched.	6-7
Search	8	Present full electronic search strategy for at least one database, including any limits used, such that it could be repeated.	5, Suppl. File 1
Study selection	9	State the process for selecting studies (i.e., screening, eligibility, included in systematic review, and, if applicable, included in the meta-analysis).	7
Data collection process	10	Describe method of data extraction from reports (e.g., piloted forms, independently, in duplicate) and any processes for obtaining and confirming data from investigators.	7
Data items	11	List and define all variables for which data were sought (e.g., PICOS, funding sources) and any assumptions and simplifications made.	8-9
Risk of bias in individual studies	12	Describe methods used for assessing risk of bias of individual studies (including specification of whether this was done at the study or outcome level), and how this information is to be used in any data synthesis.	9
Summary measures	13	State the principal summary measures (e.g., risk ratio, difference in means).	9-10
Synthesis of results	14	Describe the methods of handling data and combining results of studies, if done, including measures of consistency (e.g., I^2) for each meta-analysis.	9-11

For peer review only

PRISMA 2009 Checklist

Section/topic	#	Checklist item	Reported on page #
Risk of bias across studies	15	Specify any assessment of risk of bias that may affect the cumulative evidence (e.g., publication bias, selective reporting within studies).	11
Additional analyses	16	Describe methods of additional analyses (e.g., sensitivity or subgroup analyses, meta-regression), if done, indicating which were pre-specified.	10-11
**RESULTS**			
Study selection	17	Give numbers of studies screened, assessed for eligibility, and included in the review, with reasons for exclusions at each stage, ideally with a flow diagram.	12-13, Figure 1
Study characteristics	18	For each study, present characteristics for which data were extracted (e.g., study size, PICOS, follow-up period) and provide the citations.	13, Suppl. Tables 2, 3, 4
Risk of bias within studies	19	Present data on risk of bias of each study and, if available, any outcome level assessment (see item 12).	14, Suppl. Figure 2
Results of individual studies	20	For all outcomes considered (benefits or harms), present, for each study: (a) simple summary data for each intervention group (b) effect estimates and confidence intervals, ideally with a forest plot.	14-19, Figures 2-7, Suppl. Table 5
Synthesis of results	21	Present results of each meta-analysis done, including confidence intervals and measures of consistency.	14-19, Figures 2-7, Suppl. Table 5
Risk of bias across studies	22	Present results of any assessment of risk of bias across studies (see Item 15).	14, Suppl. Figure 2
Additional analysis	23	Give results of additional analyses, if done (e.g., sensitivity or subgroup analyses, meta-regression [see Item 16]).	14-19, Figures 2-7, Suppl. Table 5
**DISCUSSION**			
Summary of evidence	24	Summarize the main findings including the strength of evidence for each main outcome; consider their relevance to key groups (e.g., healthcare providers, users, and policy makers).	19, 21
Limitations	25	Discuss limitations at study and outcome level (e.g., risk of bias), and at review-level (e.g., incomplete retrieval of identified research, reporting bias).	20-21
Conclusions	26	Provide a general interpretation of the results in the context of other evidence, and implications for future research.	19-21
**FUNDING**			
Funding	27	Describe sources of funding for the systematic review and other support (e.g., supply of data); role of funders for the systematic review.	22-23

For peer review only

PRISMA 2009 Checklist

From: Moher D, Liberati A, Tetzlaff J, Altman DG, The PRISMA Group (2009). Preferred Reporting Items for Systematic Reviews and Meta-Analyses: The PRISMA Statement. PLoS Med 6(7): e1000097. doi:10.1371/journal.pmed1000097

For more information, visit: **www.prisma-statement.org**.

For peer review only

For peer review only - http://bmjopen.bmj.com/site/about/guidelines.xhtml
